# Synthesis of Sulfur-Substituted Bicyclo[1.1.1]pentanes by Iodo-Sulfenylation of [1.1.1]Propellane

**DOI:** 10.1021/acs.orglett.2c02875

**Published:** 2022-09-21

**Authors:** Sarah Livesley, Bethany Trueman, Craig M. Robertson, William R. F. Goundry, James A. Morris, Christophe Aïssa

**Affiliations:** † Department of Chemistry, University of Liverpool, Crown Street, Liverpool L69 7ZD, United Kingdom; ‡ Early Chemical Development, Pharmaceutical Sciences, R&D, AstraZeneca, Macclesfield SK10 2NA, United Kingdom; § Syngenta, International Research Centre, Bracknell, Berkshire RG42 6EY, United Kingdom

## Abstract

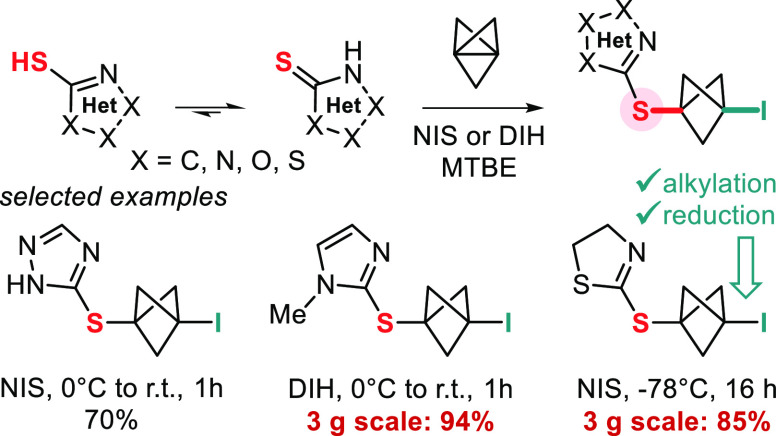

Thiols easily react with [1.1.1]propellane to give sulfur-substituted bicyclo[1.1.1]pentanes in radical reactions, but this reactivity is not replicated in the case of heterocyclic thiols. Herein, we address this issue by electrophilically activating [1.1.1]propellane to promote its iodo-sulfenylation with 10 classes of heterocyclic thiols in two protocols that can be conducted on a multigram scale without exclusion of air or moisture.

Bicyclo[1.1.1]pentanes (BCPs) often improve the potency, metabolic stability, and water solubility of bioactive compounds.^1^ These valuable properties have spurred the recent emergence of numerous methods for the synthesis of BCPs from [1.1.1]propellane.^2,3^ Although sulfur is the third most abundant heteroelement in drugs after nitrogen and oxygen,^4^ sulfur-substituted BCPs (S-BCPs) are strikingly scarce in the patent literature.^5^ The synthesis of S-BCPs has been reported by radical reactions of [1.1.1]propellane **1** with thiols,^6^ disulfides,^7^ xanthates,^8^ thiosulfonates,^9^ or sulfones (Figure 1a).^10^ Moreover, BCP sulfones and sulfonamides can be accessed from BCP sulfinates.^11^ However, although the addition of aromatic thiols to **1** has been known for several decades to be facile at room temperature, their heterocyclic counterparts **2–4** fail to react with **1** under the same conditions.^12^ These limitations restrict the exploration of the potential benefits of S-BCPs as bioisosteric replacements of *para*-substituted benzene rings and *tert*-butyl group in bioactive compounds, as for example antifungal **5**(13) and biocide **6** (Figure 1b).^14^

**Figure 1:**
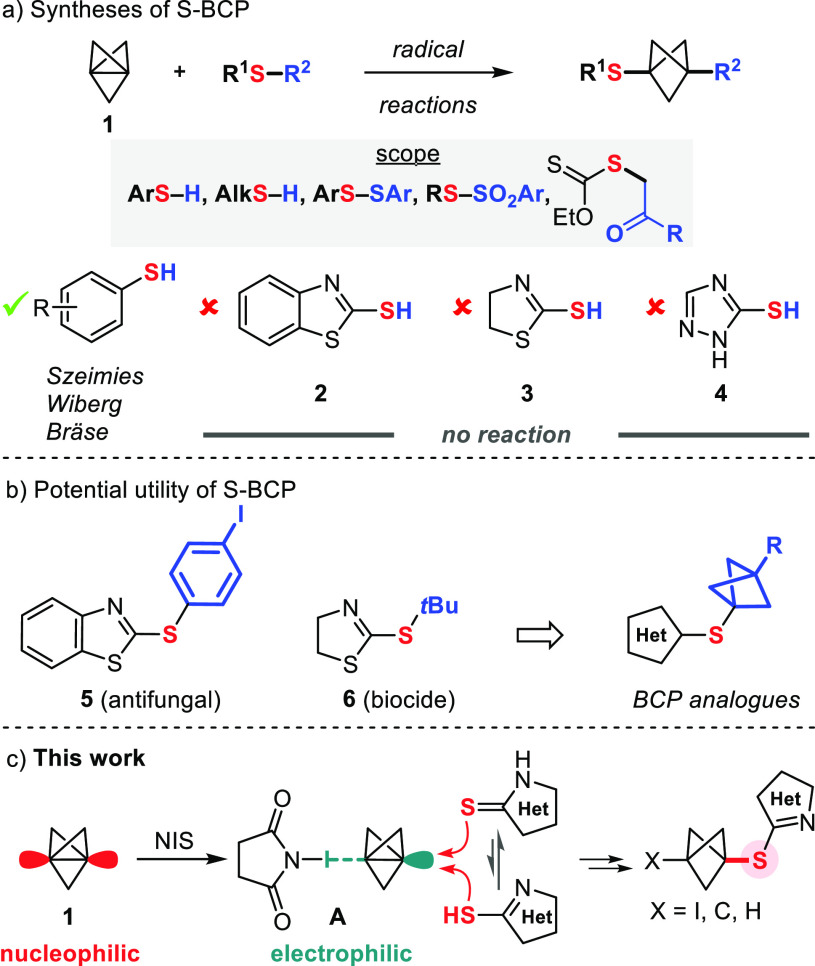
Sulfur-substituted bicyclo[1.1.1]pentanes (S-BCPs). (a) Previous syntheses of S-BCPs and failure of 2-mercapto-azoles and thiazoline. (b) Potential S-BCP analogues of bioactive compounds. (c) Iodo-sulfenylation of [1.1.1]propellane (this work).

The reaction of thiols with **1** has been suggested to proceed by the reversible addition of a thiyl radical and the transfer of a hydrogen atom to the resulting bicyclo[1.1.1]pentyl radical.^15^ The reported rates of addition of thiyl radicals to olefins suggest that the apparent lack of reactivity of **2–4** with **1** in radical reactions is unlikely due to a slower addition of those thiyl radicals to **1**(16) or differences in bond dissociation energies.^16c^ Instead, it might be imputable to a polarity mismatch in the hydrogen atom transfer between heterocyclic thiol and the bicyclo[1.1.1]pentyl radical intermediate,^17^ because heterocyclic thiols are less hydridic than aryl or alkyl thiols.^18^ Alternatively, or in addition to this reasoning, the low concentration of heterocyclic thiols in solution created by the predominance of the thione tautomer^19^ would decrease the rates of addition of the thiyl radical to **1** and of the transfer of a hydrogen atom to the bicyclo[1.1.1]pentyl radical.

Previously, we established in collaboration with the Duarte group that electrophilic activation of **1** in halogen bond complex **A** (Figure 1c),^20^ formed between propellane **1** and electrophilic reagents such as *N*-iodosuccinimide (NIS), is a viable method for promoting reactions of the interbridgehead bond of **1** with weak nucleophiles. We therefore wondered whether a similar strategy, which does not rely on a radical mechanism, could be applicable to heterocyclic thiols and thus overturn their lack of reactivity with **1** in radical reactions. Herein, we describe the successful deployment of this strategy for the iodo-sulfenylation of **1** with 10 classes of heterocyclic thiols under conditions that do not require dry reagents and solvents or an inert atmosphere (Figure 1c).

Following our previous report on the reaction of anilines with propellane **1** and NIS in acetone,^20^ we examined these conditions with **2** (Table 1, entry 1). The desired adduct **7a**, a direct bioisosteric analogue of antifungal **5**,^13^ was obtained as a bench-stable solid, and its structure was also confirmed by X-ray crystallography. However, we were surprised to observe the formation of 1,3-bis-iodo-BCP **8** in large amounts. Among the solvents examined (entries 1–6), ethers (entries 5 and 6) were best for keeping the **7a**/**8** ratio at an optimal level. Decreasing the stoichiometry of propellane **1** and NIS further decreased the amount of unwanted **8** (entries 8 and 9). Conversely, the extent of formation of **8** was increased when molecular iodine was used instead of NIS (entry 10). Similarly, the conditions previously reported by Zarate and co-workers for the attack of **1** by 4-iodo-pyrazole in the presence of I_2_ and Cs_2_CO_3_ in MeCN^21^ led to unfavorable **7a**/**8** ratios when applied to **2** (Table S1). Finally, attempts to extend this electrophilic activation with *N*-bromo- and *N*-chlorosuccinimide did not afford the expected BCP products.

**Table 1:**
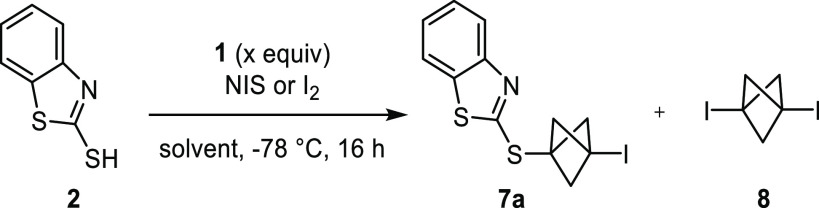
Optimization of the Reaction Conditions^a^,^b^

run	*x*^c^	iodination reagent	solvent	yield of **7a** (%)	yield of **8** (%)
1	1.5	NIS (1.5 equiv)	acetone	80	28
2	1.5	NIS (1.5 equiv)	CH_2_Cl_2_	77	11
3	1.5	NIS (1.5 equiv)	EtOAc	80	18
4	1.5	NIS (1.5 equiv)	toluene	0	0
5	1.5	NIS (1.5 equiv)	Et_2_O	98	10
6	1.5	NIS (1.5 equiv)	MTBE	99	12
7	1.5	NIS (1.1 equiv)	MTBE	99	7
8	1.1	NIS (1.1 equiv)	MTBE	99	2
9	1.1	NIS (1.0 equiv)	MTBE	99	2
10	1.5	I_2_ (0.75 equiv)	MTBE	36	42

a Reactions conducted with 0.2 mmol of **2** (0.2 M) and using a 0.85–1.10 M solution of **1** in Et_2_O.

b Yields determined by ^1^H NMR with CH_2_Cl_2_ as the internal standard. MTBE denotes methyl *tert*-butyl ether.

c Number of equivalents of **1**.

With the optimized conditions in hands, we examined the generality of the reaction with a set of diverse mercapto reagents and were delighted to obtain **7a–n** in 11–94% yields as air-stable compounds (Figure 2).^22^ Hence, mercapto reagents **2–4**, which previously failed to react with propellane **1** without an electrophilic activating reagent,^12^ gave **7a**, **7j**, and **7k**, respectively, readily in the presence of NIS. It is noteworthy that the reaction does not require any dry reagents or solvents. In the case of **7g**, it was necessary to use 1,3-diiodo-hydantoin (DIH) instead of NIS for ease of purification, and the reaction was conducted at room temperature after adding the reagents at −10 °C because of the poor solubility of the starting material at −78 °C. These conditions and the conditions optimized in entry 9 of Table 1 were compatible with reactions conducted on a multigram scale, as shown by the excellent yields of **7g** (94%) and **7k** (85%) thus obtained. It is also noteworthy that the clean conversion of the starting materials to these compounds allowed for purification by simple filtration of the crude material over a short pad of silica gel. The stoichiometry of the mercapto reagent in the reaction leading to **7j** was slightly increased compared to that under the optimized conditions due to the poor solubility of this starting material.

**Figure 2:**
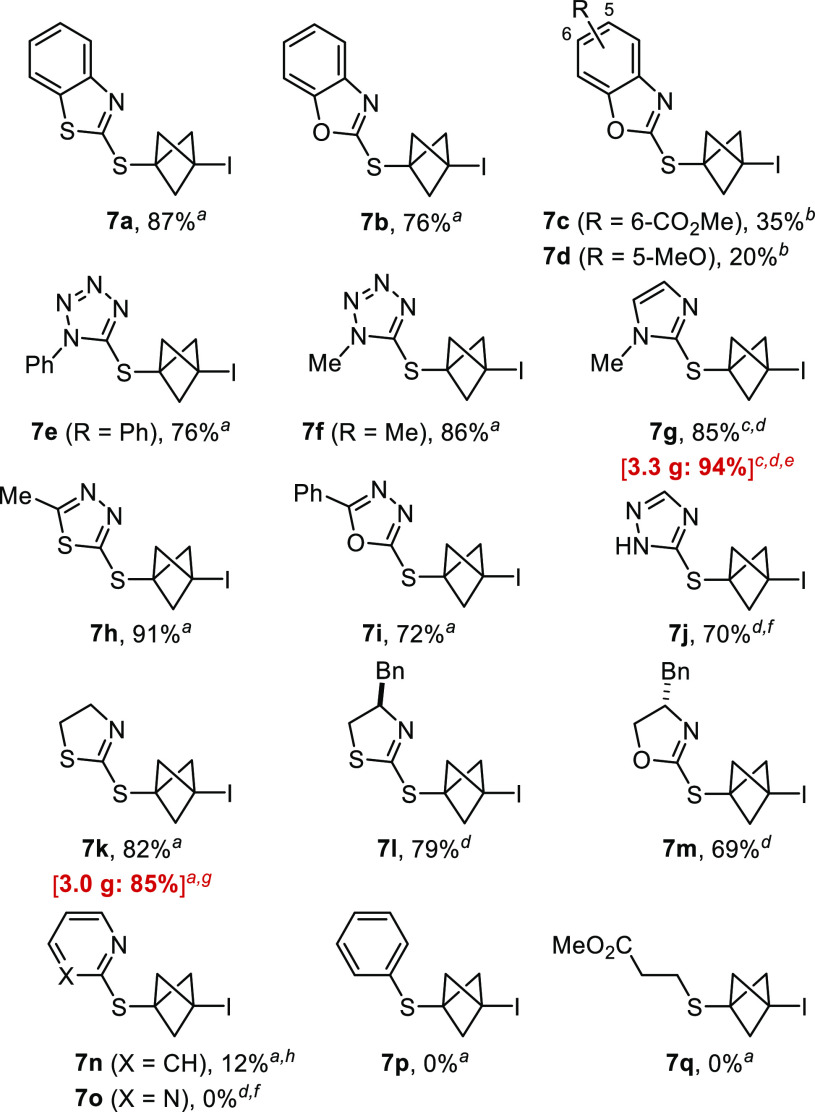
Iodo-sulfenylation of propellane **1**. Yields of pure isolated products. ^*a*^Same reaction conditions as in entry 9 of Table 1, except as otherwise noted. ^*b*^In acetone. ^*c*^DIH (0.50 equiv) instead of NIS. ^*d*^At −10 °C for 10 min and then room temperature for 1 h. ^*e*^On 11.4 mmol of mercapto reagent. ^*f*^Mercapto reagent (1.5 equiv), NIS (1.1 equiv), and **1** (1.0 equiv). ^*g*^On 11.2 mmol of mercapto reagent. ^*h*^Mercapto reagent (1.0 equiv), NIS (1.0 equiv), and **1** (2.0 equiv).

In contrast to the 10 classes of heterocyclic thiols that showed the desired reactivity to give **7a**, **7b**, and **7e–m**, electronic variation of the benzo[*d*]oxazole ring led to decreased yields in the case of **7c** and **7d** (Scheme 1). In these two cases, the solubility of the starting thiols was low in MTBE and we switched the solvent to acetone. However, the solubility remained problematic, which led to incomplete conversion and the isolation of 1,3-bisiodo-BCP **8** as a side product in 27% and 29% yields. Moreover, 2-mercaptopyridine gave **7n** in only low yield, whereas 2-mercaptopyrimidine, thiophenol, and an alkyl thiol failed to give **7o–q** entirely. The disulfides resulting from the oxidation of the thiols were the major components of the crude mixtures in these four cases.

**Scheme 1:**
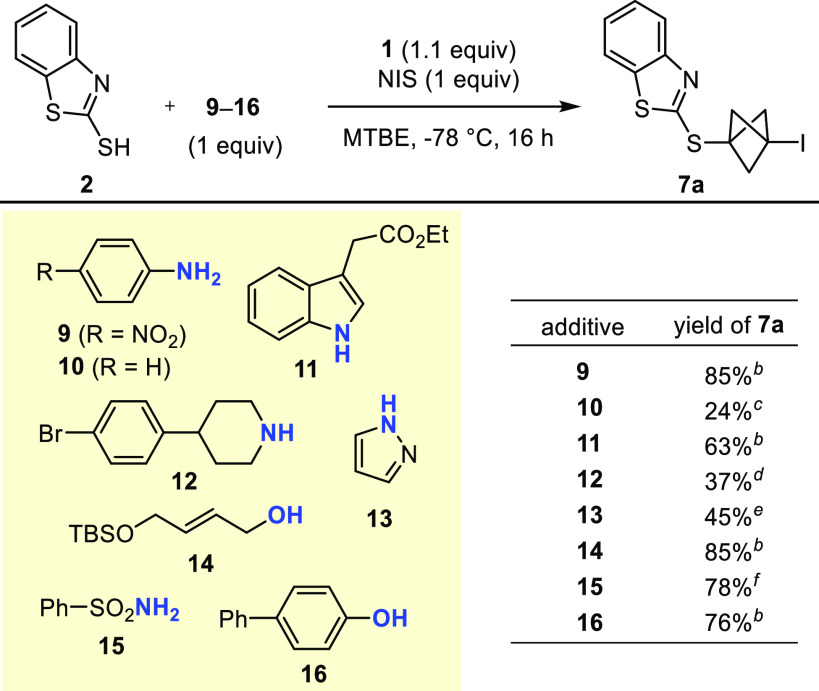
Functional Group Tolerance Yields of isolated products. Additive recovered in >80% yield (see the Supporting Information). With **8** (31%). At a 4/1 **7a**/**8** ratio (crude ^1^H NMR). With **8** (23%). Recovery of **15** not attempted.

The functional group tolerance of the reaction was evaluated with 2-mercaptobenzothiazole **2** in the presence of nucleophilic additives **9–16** (Scheme 2). The expected BCP **7a** was obtained in all cases, albeit in varied yields. Importantly, no BCP adduct was formed from **9–16** in those reactions, even in cases in which the yield of isolated **7a** was lower than in the absence of those additives. Thus, whereas electron-poor aniline **9** reacted smoothly with propellane **1** and NIS at −78 °C to give a stable iodinated BCP when no other nucleophile was present,^20^ treating an equimolar mixture of **2** and **9** under similar conditions left **9** intact and gave **7a** exclusively. Other nucleophiles, i.e., indole **11**, alcohol **14**, sulfonamide **15**, and phenol **16**, were also perfectly well tolerated to give good to high yields of **7a**. In contrast, adding electron-neutral aniline **10**, amine **12**, and pyrazole **13** led to a decreased yield of **7a** and a sizable amount of 1,3-bisiodo-BCP **8**.

**Scheme 2:**
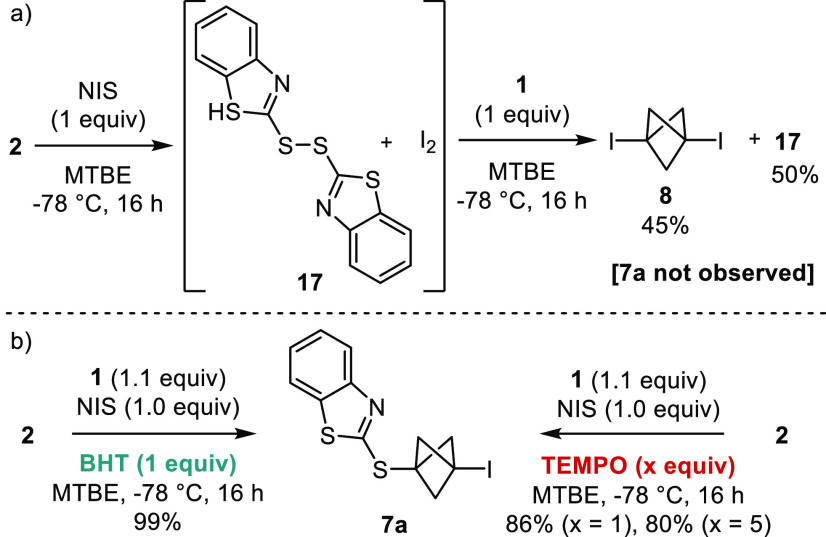
Control Reactions (a) Reaction of 2-mercaptobenzothiazole with NIS and treatment of the crude thus obtained with [1.1.1]propellane and (b) reactions in the presence of radical inhibitors. All yields determined by ^1^H NMR with an internal standard. BHT denotes 2,6-bis(*tert*-butyl)-4-methylphenol, and TEMPO 2,2,5,5-tetramethyl-4-piperidin-1-oxyl.

To gain insight into the mechanism of this reaction, we treated 2-mercaptobenzothiazole **2** with NIS in the absence of propellane **1**, which led to a mixture of disulfide **17** and molecular iodine (Scheme 2a). Importantly, when this crude mixture was treated with **1**, only 1,3-bis-iodo-BCP **8** (45%) and **17** (50%) were obtained, whereas S-BCP **7a** was not observed. In addition, treating **8** with **2** did not lead to the formation of **7a** (see the Supporting Information). These results suggest that a hypoiodothioite intermediate, or a S···I bond complex^23^ formed between NIS and the thione tautomer of the mercapto reagent, is not involved in the formation of S-BCPs **7a–m**. Moreover, the reactions of **1** with **2** and NIS under the optimized conditions but in the presence of radical inhibitors BHT and TEMPO led to the formation of the expected S-BCP **7a** in excellent to quantitative yields (Scheme 2b). Taken together, these results make a radical mechanism for the iodo-sulfenylation of **1** with 2-mercapto-azoles and NIS less likely.

Accordingly, we propose that the formation of S-BCPs **7a–m** proceeds by the electrophilic activation of propellane **1** in halogen bond complex **A** formed with the electrophilic *N*-iodo reagent (Scheme 3). As previously established,^20^ the analysis of Fukui’s dual descriptor^24^ indicates that the nucleophilic interbridgehead bond of propellane **1** is rendered electrophilic in **A**, which is a true minimum with a binding energy of −4.5 kcal mol^–1^. The high yields of formation of **7a–m** contrast with the absence of S-BCPs **7o** and **7p** when model aryl and alkyl thiols were used. These opposite results might be explained by the predominance of the thione tautomer of the 2-mercapto-azoles in solution.^19^ Thus, the low concentration of the thiol tautomer of the 2-mercapto-azoles would contribute to the high yields of **7a–m** as it would favor the selective reaction of NIS with **1** to give **A** over the reaction of NIS with the thiol. The latter pathway leads to the formation of disulfides and molecular iodine, and eventually 1,3-bis-iodo-BCP **8**, and is therefore detrimental to the formation of **7a–m**. This unproductive pathway was followed by aryl and alkyl thiols that failed to give **7p** and **7q** because a tautomeric equilibrium toward a thione is not possible. In agreement with this interpretation, treating an equimolar mixture of 2-mercapto-benzothiazole **2** and thiophenol under the optimized conditions led to the quantitative formation of phenyl disulfide and the recovery of **2** in 68% yield, whereas S-BCP **7a** was not formed. Once **A** is formed selectively, it is not certain which of the thione or thiol tautomers of the 2-mercapto-azoles reacts with this intermediate to give **7a–m**. In the case of 2-mercaptopyridine and 2-mercaptopyrimidine, we assume that the efficient formation of **7n** and **7o** could be hampered by either (i) lower oxidation potentials compared to those of the other 2-mercapto-azoles,^25^ (ii) greater aromatic character in both of its tautomeric forms that would decrease nucleophilicity,^26^ or (iii) a combination of the two.

**Scheme 3:**
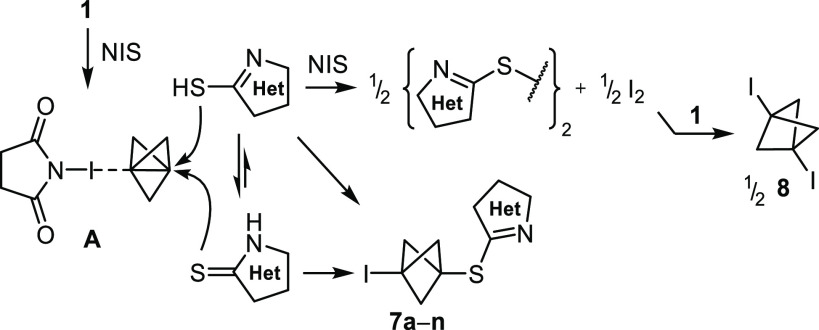
Plausible Mechanism

Finally, the conversion of the C–I bond of the S-BCP into other bonds under radical conditions proved to be challenging. Thus, for model substrates **7a**, **7e**, and **7g**, attempts to reduce the C–I bond or to engage these compounds into a Giese reaction led to decomposition by cleavage of the C(sp^3^)–S bond of the starting material. However, thiazoline derivative **7k** was more stable under the same reaction conditions (Scheme 4), and we could obtain the reduced S-BCP **18** in excellent yield. It is noteworthy that **18** is a direct bioisosteric analogue of biocide **6**. Similarly, compound **19** was obtained after Giese reaction under the conditions recently described by Anderson and co-workers.^3n^ The moderate yield of **19** is due to the need to perform a purification by preparative TLC of the material obtained after a first purification by flash chromatography.

**Scheme 4:**
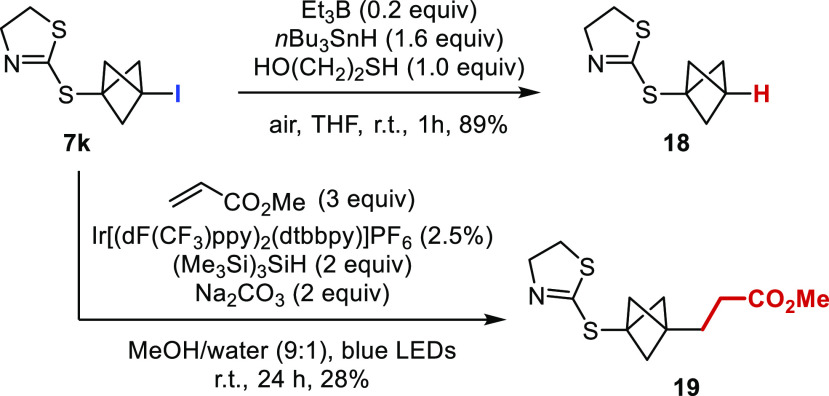
Conversion of the C–I Bond Yields of isolated product.

In conclusion, we have demonstrated that the electrophilic activation of [1.1.1]propellane with NIS or DIH can address the lack of reactivity of heterocyclic thiols for the synthesis of sulfur-substituted bicyclo[1.1.1]pentanes. The procedure can be conducted on a multigram scale and does not require exclusion of air or moisture. We anticipate that this method could benefit the future exploration of the potential benefits of S-BCPs in the optimization of the bioactivity of drugs and agrochemicals.

## Supporting Information Available

Experimental details for the synthesis of **7a–n**, control experiments, DSC data (**7e**), and characterization data of new compounds (PDF)
