# Supplementary material for: Synthesis of Sulfur-Substituted Bicyclo[1.1.1]pentanes by Iodo-Sulfenylation of [1.1.1]Propellane
Source: Org Lett. 2022 Sep 21;24(38):7015–20. doi: 10.1021/acs.orglett.2c02875 (PMC9531248; doi:10.1021/acs.orglett.2c02875)
Supplement: Supplementary file 1 — ol2c02875_si_001.pdf [file ol2c02875_si_001.pdf]

Supporting Information

Synthesis of Sulfur-Substituted Bicyclo[1.1.1]pentanes by Iodo-Sulfenylation of [1.1.1]Propellane

Sarah Livesley,† Bethany Trueman,† Craig M. Robertson,† William R. F. Goundry,§ James A. Morris,‡ and Christophe Aïssa*,†

† Department of Chemistry, University of Liverpool, Crown Street, L69 7ZD, United Kingdom.

‡ Syngenta, International research Centre, Bracknell, Berkshire, RG42 6EY, United Kingdom.

§ Early Chemical Development, Pharmaceutical Sciences, R&D, AstraZeneca, Macclesfield, SK10 2NA, United Kingdom.

*E-mail: aissa@liverpool.ac.uk

Table S1 – Unfavorable conditions for the selective formation of **7a** S2

Synthesis and spectroscopy data of compounds **7a**–**7n** S3

Functional group tolerance (with preparation of **14**; isolation of **8**) S11

Control reactions S12

Functionalization of the C–I bond (synthesis and spectroscopic data of **18** and **19**) S15

References S17

Copies of NMR spectra of compounds **7a**–**7n**, **8, 14, 17 (crude), 18** and **19** S18

HMBC of compound **18** S50

HMBC of compound **19** S51

Crystal data and structure refinement for compound **7a** S50

Crystal data and structure refinement for compound **7e** S51

DSC for compound **7e** S52

General

All thiols were purchased from commercial supplier and were used as received. [1.1.1]Propellane (**1**) was prepared as a stock solution in Et_2O according to the procedure reported by Baran.[1] Light-promoted alkylation: Kessil® lamps (Tuna-blue, A160WE, 40W). Flash chromatography: Merck silica gel 60 (230-400 mesh). NMR: the ^1H and $^{13}C\{^1H\}$ spectra were recorded on a Bruker DRX 500 or on a Bruker Avance 400 spectrometer. Chemical shifts (δ) are given in ppm. The solvent signals were used as references for ^1H and $^{13}C\{^1H\}$ spectra ($CDCl_3$: $\delta_H = 7.26$, $\delta_C = 77.0$; DMSO-d_6: $\delta_H = 2.50$, $\delta_C = 39.5$). IR spectra were recorded on a PerkinElmer Spectrum 100 FT-IR spectrometer, and the wavenumbers ($\tilde{\nu}$) are given in cm^{-1}. HRMS determined at the University of Liverpool on Agilent 6540A Accurate-Mass Q-ToF MS with Agilent Jetstream Source (ESI); *m/z* values were calculated using the software Agilent MassHunter Qualitative Analysis Navigator. Melting points were measured on a Griffin melting point apparatus (not corrected). Elemental analyses: Elementar Vario Micro Cube instrument at University of Liverpool. Optical rotation: Bellingham Stanley ADP440+ (c are given in g/100 mL).

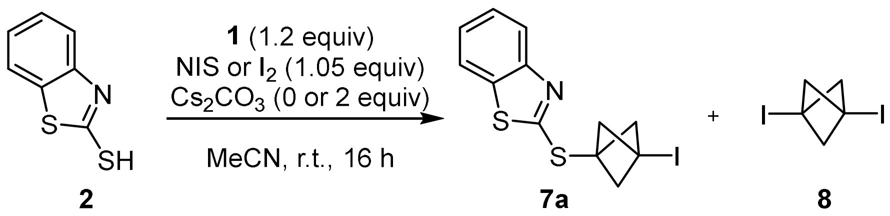

Run	Electrophile	Cs_2CO_3	7a	8
1	NIS	None	79%	28%
2	I_2	None	37%	16%
3	NIS	2 equiv	69%	28%
4	I_2	2 equiv	25%	14%

Table S1. Unfavorable conditions[2] for the selective formation of **7a**. Yields determined by ^{1}H NMR with CH_2Cl_2 as internal standard

Synthesis and spectroscopy data of compounds 7a–7n

Representative procedure for the iodo-sulfenylation of [1.1.1]propellane. A test tube equipped with a stirring bar was charged under air with the relevant thiol (0.2 mmol, 1.0 equiv) and MTBE (1 mL, 0.2 M), then cooled to -78 °C in a cryogenic bath fitted with a cold finger. [1.1.1]Propellane (**1**) (0.22 mmol, 1.1 equiv, 0.85–1.10 M stock solution in Et_2O) and NIS (45 mg, 0.2 mmol, 1.0 equiv) were added in succession. The reaction vessel was sealed with a septum and the mixture was stirred at -78 °C for 16 h. The tube was then taken out of the cold bath and its content transferred to a round-bottom flask by rinsing with CH_2Cl_2 twice. Silica was added and the volatiles were removed *in vacuo*. The crude reaction mixture thus loaded on silica was purified by flash column chromatography on silica gel using the conditions specified below.

2-((3-iodobicyclo[1.1.1]pentan-1-yl)thio)benzo[*d*]thiazole (7a). Obtained from 2-mercaptobenzothiazole (33 mg, 0.2 mmol) following the representative procedure and after purification by flash chromatography (hexanes, then ethyl acetate/hexanes (1:99)): 63 mg, 87%. White solid; m.p.: 72–73 °C. ^{1}H NMR (500 MHz, $CDCl_3$): δ 7.92 (d, J = 8.3 Hz, 1H), 7.78 (d, J = 8.0 Hz, 1H), 7.46–7.43 (m, 1H), 7.36–7.33 (m, 1H), 2.73 (s, 6H). ^{13}C{^{1}H} NMR (125 MHz, $CDCl_3$): δ 163.0, 153.2, 135.7, 126.3, 124.9, 122.3, 121.0, 63.6 (3C), 44.0, 2.4. IR (neat): $\tilde{\nu}$ = 3061 (w), 3018 (w), 2977 (w), 2922 (w), 2880 (w), 1592 (w), 1496 (m), 1459 (m), 1416 (m), 1387 (m), 1240 (m), 1192 (s), 1092 (m), 1015 (m), 991 (m), 973 (m), 919 (w), 901 (m), 860 (m), 844 (m), 761 (s), 693 (m), 684 (m). HRMS (ESI$^+$): *m/z* calculated for $C_{12}H_{10}INS_2$ $[M+H]^+$: 359.9372; found: 397.9371. Elemental analysis (%) calculated for $C_{12}H_{10}INS_2$: C 40.12, H 2.81, N 3.90, S 17.85; found: C 40.13, H 3.27, N 4.17, S 18.07.

2-((3-iodobicyclo[1.1.1]pentan-1-yl)thio)benzo[*d*]oxazole (7b). Obtained from 2-mercaptobenzoxazole (30 mg, 0.2 mmol) following the representative procedure and after purification by flash chromatography (hexanes, then ethyl acetate/hexanes (2:98)): 52 mg, 76%. White solid; m.p.: 61–64 °C. ^{1}H NMR (500 MHz, $CDCl_3$): δ 7.62–7.61 (m, 1H), 7.46–7.45 (m, 1H), 7.32–7.27 (m, 2H), 2.77 (s, 6H). ^{13}C{^{1}H} NMR (125 MHz, $CDCl_3$): δ 161.9, 151.5, 141.8, 124.4, 124.3, 119.0, 109.9, 63.4 (3C), 42.0, 2.0. IR (neat): $\tilde{\nu}$ = 3208 (w), 3011 (w), 2979 (w), 2922 (w), 2880 (w), 1499 (s), 1452 (s), 1238 (m), 1211 (m), 1195 (s), 1134 (s), 1125 (s), 1095 (s), 903 (m), 846 (s), 807 (m), 751 (m), 746 (m), 735 (s). HRMS (ESI$^+$): *m/z* calculated for $C_{12}H_{10}INOS$

[M+H]$^+$: 343.9601; found: 343.9597. Elemental analysis (%) calculated for $C_{12}H_{10}INOS$: C 42.00, H 2.94, N 4.08, S 9.34; found: C 42.06, H 3.16, N 4.13, S 9.03.

Methyl 2-((3-iodobicyclo[1.1.1]pentan-1-yl)thio)benzo[d]oxazole-6-carboxylate (7c). Obtained from methyl 2-mercaptobenzo[d]oxazole-6-carboxylate (31.4 mg, 0.15 mmol) following the representative procedure except that acetone was used as solvent and after purification by flash chromatography (ethyl acetate/hexanes (2:98 to 5:95)): 21 mg, 35%. White solid; m.p.: 103–106 °C. ^{1}H NMR (500 MHz, $CDCl_3$): δ 8.13 (d, J = 1.4 Hz, 1H), 8.04 (dd, J = 8.4, 1.5 Hz, 1H), 7.62 (d, J = 8.4 Hz, 1H), 3.95 (s, 3H), 2.79 (s, 6H). ^{13}C{^{1}H} NMR (125 MHz, $CDCl_3$): δ 166.5, 165.6, 151.0, 145.7, 126.4, 126.3, 118.3, 63.4 (3C), 52.4, 41.7, 1.8. IR (neat): $\tilde{\nu}$ = 3012 (w), 2957 (w), 2919 (w), 1713 (s), 1622 (m), 1595 (w), 1490 (m), 1429 (m), 1343 (m), 1288 (m), 1262 (m), 1223 (m), 1197 (s), 1130 (m), 1107 (m), 1073 (m), 1032 (m), 966 (m), 923 (w), 899 (m), 883 (m), 859 (m), 837 (m), 800 (m), 767 (s), 739 (m). HRMS (ESI$^+$): *m/z* calculated for $C_{14}H_{12}INO_3S$ [M+H]$^+$: 401.9655; found: 401.9655. Elemental analysis (%) calculated for $C_{14}H_{12}INO_3S$: C 41.91, H 3.01, N 3.49, S 7.99; found: C 42.51, H 3.34, N 3.45, S 7.69. Note: 1,3-diiodobicyclo[1.1.1]pentane (**8**) was also isolated from this reaction (14 mg, 27%). ^{1}H NMR (500 MHz, $CDCl_3$): δ 2.68 (s, 6H); the data is in agreement with the literature.[4]

2-((3-iodobicyclo[1.1.1]pentan-1-yl)thio)-5-methoxybenzo[d]oxazole (7d). Obtained from methyl 5-methoxybenzo[d]oxazole-2-thiol (27.2 mg, 0.15 mmol) following the representative procedure except that acetone was used as solvent and after purification by flash chromatography (ethyl acetate/hexanes (2:98 to 5:95)): 11 mg, 20%. Colorless oil. ^{1}H NMR (500 MHz, $CDCl_3$): δ 7.33 (d, J = 8.8 Hz, 1H), 7.12 (d, J = 2.6 Hz, 1H), 6.86 (dd, J = 8.9, 2.5 Hz, 1H), 3.85 (s, 3H), 2.76 (s, 6H). ^{13}C{^{1}H} NMR (125 MHz, $CDCl_3$): δ 162.4, 157.3, 146.1, 142.5, 112.6, 110.0, 102.3, 63.5 (3C), 56.0, 42.0, 2.0. IR (neat): $\tilde{\nu}$ = 3003 (w), 2974 (w), 2920 (w), 2832 (w), 2344 (w), 2044 (w), 1612 (m), 1496 (m), 1477 (s), 1435 (s), 1336 (w), 1282 (m), 1260 (w), 1218 (w), 1194 (s), 1147 (s), 1133 (s), 1097 (m), 1024 (s), 945 (m), 905 (m), 858 (s), 831 (s), 801 (m), 764 (w), 755 (m). HRMS (ESI$^+$): *m/z* calculated for $C_{13}H_{12}INO_2S$ [M+H]$^+$: 373.9706; found: 373.9705. Note: 1,3-diiodobicyclo[1.1.1]pentane (**8**) was also isolated from this reaction (15 mg, 29%).

5-((3-iodobicyclo[1.1.1]pentan-1-yl)thio)-1-phenyl-1*H*-tetrazole (7e). Obtained from 1-phenyl-1*H*-tetrazole-5-thiol (36 mg, 0.2 mmol) following the representative procedure and after purification by flash chromatography (ethyl acetate/hexanes (5:95 to 10:90)): 56 mg, 76%. White solid; m.p: 137–138 °C. ^{1}H NMR (500 MHz, $CDCl_3$): δ 7.54–7.58 (m, 3H), 7.49-7.51 (m, 2H), 2.75 (s, 6H). ^{13}C{^{1}H} NMR (125 MHz, $CDCl_3$): δ 152.4, 133.5, 130.4, 129.8 (2C), 124.0 (2C), 63.3 (3C), 42.2, 1.5. IR (neat): $\tilde{\nu}$ = 3061 (w), 3018 (w), 2978 (w), 2922 (w), 2851 (w), 1592 (w), 1496 (m), 1459 (w), 1416 (m), 1387 (m), 1314 (w), 1296 (w), 1276 (w), 1240 (w), 1192 (s), 1144 (w), 1092 (m), 1072 (w), 1015 (m), 972 (w), 919 (w), 900 (m), 860 (s), 844 (m), 762 (s), 693 (s), 684 (s). HRMS (ESI$^+$): *m/z* calculated for $C_{12}H_{11}IN_4S$ $[M+H]^+$: 370.9822; found: 370.9820. Elemental analysis (%) calculated for $C_{12}H_{11}IN_4S$: C 38.93, H 3.00, N 15.13, S 8.66; found: C 39.34, H 3.42, N 15.05, S 8.56. **CAUTION**: a DSC test shows the material is a potential explosive and has a high risk of being shock sensitive.

5-((3-iodobicyclo[1.1.1]pentan-1-yl)thio)-1-methyl-1*H*-tetrazole (7f). Obtained from 1-methyl-1*H*-tetrazole-5-thiol (23.2 mg, 0.2 mmol) following the representative procedure and after purification by flash chromatography (ethyl acetate/hexanes (5:95 to 15:85)): 53 mg, 86%. White solid; m.p: 77–80 °C. ^{1}H NMR (500 MHz, $CDCl_3$): δ 3.92 (s, 3H), 2.70 (s, 6H). ^{13}C{^{1}H} NMR (125 MHz, $CDCl_3$): δ 161.9, 63.3 (3C), 42.1, 33.6, 0.9. IR (neat): $\tilde{\nu}$ = 3008 (w), 2975 (w), 2918 (w), 1450 (m), 1409 (m), 1392 (m), 1273 (m), 1225 (w), 1191 (s), 1170 (m), 1140 (m), 1077 (m), 1037 (w), 1024 (m), 970 (m), 906 (m), 893 (w), 852 (s), 767 (w), 718 (w), 702 (m), 683 (m), 576 (w), 563 (w). HRMS (ESI$^+$): *m/z* calculated for $C_7H_9IN_4S$ $[M+H]^+$: 308.9665; found: 308.9661. Elemental analysis (%) calculated for $C_7H_9IN_4S$: C 27.29, H 2.94, N 18.18, S 10.40; found: C 27.66, H 3.24, N 17.74, S 9.97.

2-((3-iodobicyclo[1.1.1]pentan-1-yl)thio)-1-methyl-1*H*-imidazole (7g). A 250 mL round-bottom flask equipped with a stirring bar was charged with 2-mercapto-*N*-methylimidazole (1.3 g, 11.4 mmol, 1 equiv), MTBE (57 ml, 0.2 M), then cooled to -10 °C. [1.1.1]Propellane (16.7 mL, 12.5 mmol, 1.1 equiv, 0.75 M in Et_2O) was added followed by 1,3-diiodo-5,5-dimethylhydantoin (2.2 g, 5.7 mmol, 0.5 equiv) in one portion. The reaction vessel was maintained at -10 °C for 10 minutes before being warmed to room temperature and stirred for an additional hour. The reaction crude was loaded directly onto silica and added to a column loaded

with silica gel (40 g) prepared with CH_2Cl_2. The pad of silica was washed with 50 mL of CH_2Cl_2, and the eluted CH_2Cl_2 was discarded. The pad of silica was then washed with a gradient of ethyl acetate/dichloromethane (10:90 to 14:86, 750 mL) directly into a round-bottom flask (Figure S1). The solvent was removed *in vacuo* to yield **7g** (3.28 g, 94%). White solid; melting point: 70–71 °C. ^{1}H NMR (500 MHz, $CDCl_3$): δ 7.10 (d, J = 0.9 Hz, 1H), 6.99 (d, J = 1.0 Hz, 1H), 3.68 (s, 3H), 2.41 (s, 6H). $^{13}C\{^{1}H\}$ NMR (125 MHz, $CDCl_3$): δ 137.9, 129.7, 123.2, 63.4 (3C), 45.0, 33.9, 1.8. IR (neat): $\tilde{\nu}$ = 3101 (w), 3009 (w), 2992 (w), 2969 (w), 2917 (m), 2878 (w), 2851 (w), 1510 (w), 1450 (m), 1409 (w), 1337 (w), 1280 (s), 1197 (s). 1160 (w), 1144 (m), 1130 (s), 1122 (s), 1079 (w), 1033 (w), 913 (m), 905 (m), 850 (s), 753 (s), 685 (s). HRMS (ESI^+): *m/z* calculated for $C_9H_{11}IN_2S$ $[M+H]^+$: 306.9760; found: 306.9760. Elemental analysis (%) calculated for $C_9H_{11}IN_2S$: C 35.31, H 3.62, N 9.15, S 10.47; found: C 35.26, H 3.77, N 9.16, S 10.31.

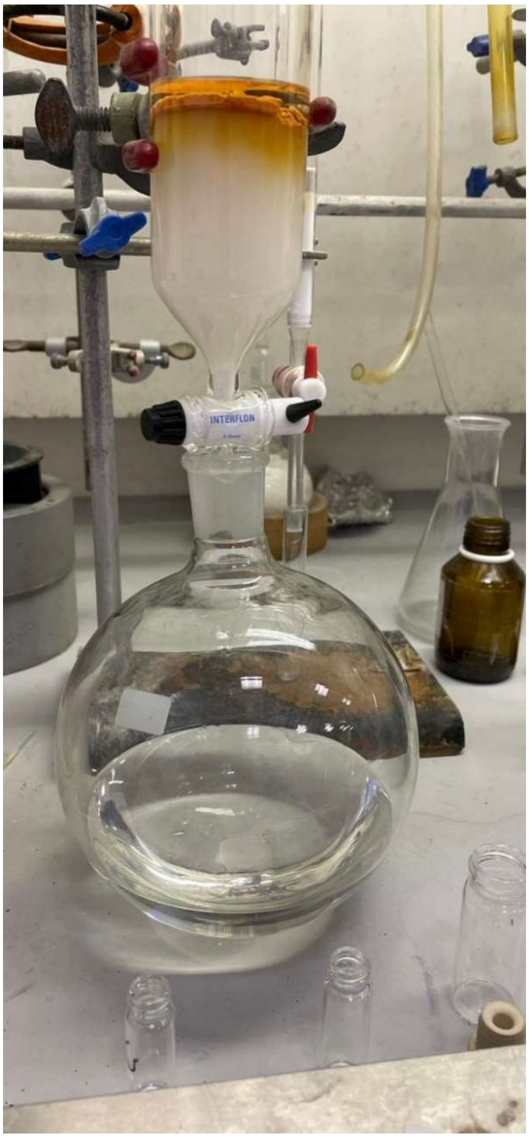

Figure S1. Multi-gram scale filtration to purify compound **7g**.

2-((3-iodobicyclo[1.1.1]pentan-1-yl)thio)-5-methyl-1,3,4-thiadiazole (7h). Obtained from 5-methyl-1,3,4-thiadiazole-2-thiol (36 mg, 0.2 mmol) following the representative procedure and after purification by flash chromatography (ethyl acetate/hexanes (5:95 to 12:88)): 59 mg, 91%. White solid; m.p: 77–91 °C. ^{1}H NMR (500 MHz, $CDCl_3$): δ 2.75 (s, 3H), 2.64 (s, 6H). $^{13}C\{^{1}H\}$ NMR (125 MHz, $CDCl_3$): δ 166.4, 161.9, 63.3 (3C), 44.0, 15.7, 1.7. IR (neat): $\tilde{\nu}$ = 3032 (w), 3016 (w), 2982 (w), 1499 (m), 1454 (m), 1241 (w), 1195 (s), 1136 (s), 1126 (m), 1097 (m), 905 (w), 846 (s), 810 (w), 753 (m), 736 (s), 720 (m), 655 (w). HRMS (ESI^+): *m/z* calculated for $C_8H_9IN_2S_2$ $[M+H]^+$: 324.9325; found: 324.9326. Elemental analysis (%) calculated for $C_8H_9IN_2S_2$: C 29.64, H 2.80, N 8.64, S 19.78; found: C 29.79, H 3.07, N 8.93, S 20.20.

2-((3-iodobicyclo[1.1.1]pentan-1-yl)thio)-5-phenyl-1,3,4-oxadiazole (7i). Obtained from 5-phenyl-1,3,4-oxadiazole-2-thiol (36 mg, 0.2 mmol) following the representative procedure and after purification by flash chromatography (hexanes to ethyl acetate/hexanes (5:95)): 54 mg, 72%. White solid; m.p: 69–72 °C. ^{1}H NMR (500 MHz, $CDCl_3$): δ 8.03–7.99 (m, 2H), 7.58–7.49 (m, 3H), 2.74 (s, 6H). $^{13}C\{^{1}H\}$ NMR (125 MHz, $CDCl_3$): δ 165.9, 161.7, 131.9, 129.1 (2C), 126.7 (2C), 123.4, 63.4 (3C), 41.9, 1.1. IR (neat): $\tilde{\nu}$ = 3065 (w), 3010 (w), 2970 (w), 2916 (w), 2878 (w), 1607 (w), 1587 (w), 1552 (m), 1471 (s), 1449 (m), 1197 (s), 1171 (m), 1132 (m), 1082 (m), 1068 (m), 1026 (w), 996 (w), 958 (w), 900 (m), 852 (s), 779 (m), 710 (s), 691 (s). HRMS (ESI^+): *m/z* calculated for $C_{13}H_{11}IN_2OS$ $[M+H]^+$: 370.9710; found: 370.9703. Elemental analysis (%) calculated for $C_{13}H_{11}IN_2OS$: C 42.18, H 3.00, N 7.57, S 8.06; found: C 42.37, H 3.16, N 7.71, S 8.59.

5-((3-iodobicyclo[1.1.1]pentan-1-yl)thio)-1*H*-1,2,4-triazole (7j). A test tube equipped with a stirring bar was charged under air with 1*H*-1,2,4-triazole-5-thiol (30.3 mg, 0.30 mmol, 1.5 equiv) and MTBE (1.0 mL, 0.2 M), then cooled to -10 °C. [1.1.1]Propellane (0.25 mL, 0.2 mmol, 1.0 equiv (0.80 M stock solution in Et_2O)) and NIS (50 mg, 0.22 mmol, 1.1 equiv) were added in succession. After stirring at -10 °C for 10 minutes and then for an additional 1 hour at room temperature, the crude reaction mixture was loaded directly onto silica and purified by flash column chromatography on silica gel (ethyl acetate/hexanes (20:80 to 40:60)) to give **7j**: 41 mg, 70%. White solid; m.p: 127–130 °C. ^{1}H NMR (500 MHz, DMSO-d_6): δ 14.36–14.10 (br s, 1H (NH)), 8.74–8.41 (br s, 1H), 2.57 (s, 6H). $^{13}C\{^{1}H\}$ NMR (125 MHz, DMSO-d_6): δ 156.6, 144.8, 62.8 (3C),

43.0, 3.7. IR (neat): $\tilde{\nu}$ = 3150–2100 (br), 1548 (w), 1515 (w), 1470 (m), 1446 (w), 1360 (w), 1278 (m), 1264 (m), 1243 (m), 1195 (s), 1177 (s), 1132 (m), 1102 (m), 1082 (m), 1001 (m), 966 (m), 901 (m), 853(s), 647 (m). HRMS (ESI$^+$): *m/z* calculated for $C_7H_8IN_3S$ $[M+H]^+$: 293.9556; found: 293.9560. Elemental analysis (%) calculated for $C_7H_8IN_3S$: C 28.68, H 2.75, N 14.34, S 10.94; found: C 28.89, H 2.98, N 14.50, S 11.08.

2-((3-iodobicyclo[1.1.1]pentan-1-yl)thio)-4,5-dihydrothiazole (7k). A 250 mL round-bottom flask equipped with a stirring bar was charged with 2-mercaptothiazoline (1.3 g, 11.2 mmol, 1 equiv), MTBE (56 mL, 0.2 M), and [1.1.1]propellane (16.4 mL, 12.3 mmol, 1.1 equiv, 0.75 M in Et_2O). The flask was cooled to -78 °C and *N*-iodosuccinimide (2.5 g, 11.2 mmol, 1 equiv) was added in one portion and the reaction was stirred at -78 °C overnight. Following completion, the crude mixture was concentrated *in vacuo* and pipetted directly onto a pad of silica gel (40 g) prepared with hexanes. The pad was washed with hexanes (50 mL), and the hexane thus eluted was discarded. The pad of silica was then washed with ethyl acetate/hexanes (1:4, 500 mL) directly into a round-bottom flask. The solvent was removed in vacuo to give **7k** (2.96 g, 85%). Pale-yellow solid; m.p.: 40–41 °C. ^{1}H NMR (500 MHz, $CDCl_3$): δ 4.20 (t, J = 8.0 Hz, 2H), 3.33 (t, J = 8.0 Hz, 2H), 2.64 (s, 6H). $^{13}C\{^1H\}$ NMR (125 MHz, $CDCl_3$): δ 163.0, 64.5, 63.5 (3C), 43.3, 35.0, 3.1. IR (neat): $\tilde{\nu}$ = 3009 (w), 2973 (w), 2918 (m), 2879 (w), 2326 (w), 1567 (m), 1479 (m), 1452 (m), 1427 (m), 1302 (m), 1195 (s), 1136 (m), 1120 (m), 1102 (w), 1082 (w), 1054 (w), 1030 (w), 1018 (w), 995 (m), 956 (m), 921 (m), 904 (s), 861 (m), 846 (s), 762 (m), 727 (w), 701 (w), 678 (w), 665 (w), 639 (m), 620 (w), 616 (w), 611 (w), 607 (w). Elemental analysis (%) calculated for $C_8H_{10}INS_2$: C 30.88, H 3.24, N 4.50, S 20.60; found: C 31.27, H 3.48, N 4.88, S 20.67.

(R)-4-benzyl-2-((3-iodobicyclo[1.1.1]pentan-1-yl)thio)-4,5-dihydrothiazole (7l). A test tube equipped with a stirring bar was charged under air with (R)-4-benzylthiazolidine-2-thione (31.4 mg, 0.15 mmol, 1.0 equiv) and MTBE (0.75 mL, 0.2 M), then cooled to -10 °C. [1.1.1]Propellane (0.28 mL, 0.165 mmol, 1.1 equiv (0.60 M stock solution in Et_2O)) and NIS (34 mg, 0.15 mmol, 1.0 equiv) were added in succession. After stirring at -10 °C for 10 minutes and then for an additional 1 hour at room temperature, the crude reaction mixture was loaded directly onto silica and purified by flash column chromatography on silica gel (hexanes then ethyl acetate/hexanes (2:98)) to give **7l**: 48 mg, 79%. Colorless oil. ^{1}H NMR (500 MHz, $CDCl_3$): δ 7.35–7.30 (m,

2H), 7.27–7.22 (m, 3H), 4.72–4.65 (m, 1H), 3.30 (dd, J = 10.9, 7.9 Hz, 1H), 3.13 (dd, J = 13.7, 5.7 Hz, 1H), 3.10 (dd, J = 11.0, 7.1 Hz, 1H), 2.80 (dd, J = 13.7, 8.5 Hz, 1H), 2.66–2.60 (m, 6H). $^{13}C\{^1H\}$ NMR (125 MHz, $CDCl_3$): δ 163.5, 138.1, 129.2 (2C), 128.5 (2C), 126.6, 77.6, 63.5 (3C), 43.3, 40.0, 38.6, 3.0. IR (neat): $\tilde{\nu}$= 3023 (w), 2972 (w), 2918 (m), 2852 (w), 1562 (s), 1495 (m), 1452 (m), 1433 (m), 1338 (w), 1304 (w), 1266 (w), 1238 (w), 1193 (s), 1132 (m), 1097 (w), 1075 (w), 1031 (m), 998 (m), 940 (s), 904 (s), 851 (s), 739 (s), 698 (s). HRMS (ESI): *m/z* calcd for $C_{15}H_{16}INS_2$ $[M + H]^+$: 401.9842; found: 401.9833. $[\alpha]^{23}_{D}$ +8.3° (c = 0.55, $CHCl_3$).

(S)-4-benzyl-2-((3-iodobicyclo[1.1.1]pentan-1-yl)thio)-4,5-dihydrooxazole (7m). A test tube equipped with a stirring bar was charged under air with (S)-4-benzyloxazolidine-2-thione (29 mg, 0.15 mmol, 1.0 equiv) and MTBE (0.75 mL, 0.2 M), then cooled to -10 °C. [1.1.1]Propellane (0.28 mL, 0.165 mmol, 1.1 equiv (0.60 M stock solution in Et_2O)) and NIS (34 mg, 0.15 mmol, 1.0 equiv) were added in succession. After stirring at -10 °C for 10 minutes and then for an additional 1 hour at room temperature, the crude reaction mixture was loaded directly onto silica and purified by flash column chromatography on silica gel (hexanes then ethyl acetate/hexanes (2:98)) to give **7m**: 40 mg, 69%. Colorless oil. ^1H NMR (500 MHz, $CDCl_3$): δ 7.33–7.28 (m, 2H), 7.26–7.18 (m, 3H), 4.45–4.37 (m, 1H), 4.22 (t_{app}, J = 8.7 Hz, 1H), 4.01 (dd, J = 8.2, 7.1 Hz, 1H), 3.05 (dd, J = 13.8, 5.7 Hz, 1H), 2.68 (dd, J = 13.8, 8.1 Hz, 1H), 2.65–2.57 (m, 6H). $^{13}C\{^1H\}$ NMR (125 MHz, $CDCl_3$): δ 163.1, 137.5, 129.2 (2C), 128.5 (2C), 126.5, 72.5, 67.9, 63.2 (3C), 41.8, 41.4, 2.8. IR (neat): $\tilde{\nu}$ = 3062 (w), 3025 (w), 2975 (w), 2919 (w), 2894 (w), 1602 (s), 1496 (w), 1471 (w), 1453 (m), 1336 (w), 1305 (w), 1263 (w), 1196 (s), 1142 (s), 1092 (m), 1066 (m), 1030 (m), 954 (m), 907 (s), 853 (s). HRMS (ESI): *m/z* calcd for $C_{15}H_{16}INOS$ $[M + H]^+$: 386.0070; found: 386.0069. $[\alpha]^{23}_{D}$ -4.4° (c = 1.25, $CHCl_3$).

2-((3-iodobicyclo[1.1.1]pentan-1-yl)thio)pyridine (7n). Obtained from pyridine-2-thiol (33 mg, 0.3 mmol, 1 equiv) following the representative procedure except that NIS (68 mg, 0.3 mmol, 1.0 equiv) and [1.1.1]propellane (**1**) (1 mL, 0.6 mmol, 2.0 equiv, 0.60 M in Et_2O) were used. Purification by flash chromatography (hexanes to ethyl acetate/hexanes (5:95) gave **7n** (10 mg, 11%). Yellow solid; m.p.: 39–42 °C; ^1H NMR (500 MHz, $CDCl_3$): δ 8.50–8.44 (m, 1H), 7.56 (dt, J = 7.7, 1.9 Hz, 1H), 7.23 (d, J = 8.0 Hz, 1H), 7.09 (ddd, J = 7.4, 5.0, 0.9 Hz, 1H), 2.67 (s, 6H). $^{13}C\{^1H\}$ NMR (125 MHz, $CDCl_3$): δ 157.7, 149.2, 136.8,

124.1, 120.7, 63.5 (3C), 43.8, 3.8. IR (neat): $\widetilde{\nu}$= 3011 (w), 2978 (w), 2920 (w), 1576 (s), 1552 (s), 1458 (s), 1189 (s), 1131 (s), 1122 (s), 1041 (m), 1031 (m), 984 (m), 914 (m), 839 (s), 759 (s), 721 (s). HRMS (ESI): *m/z* calcd for $C_{10}H_{10}INS$ $[M + H]^+$: 303.9651; found: 303.9649.

Failed attempts

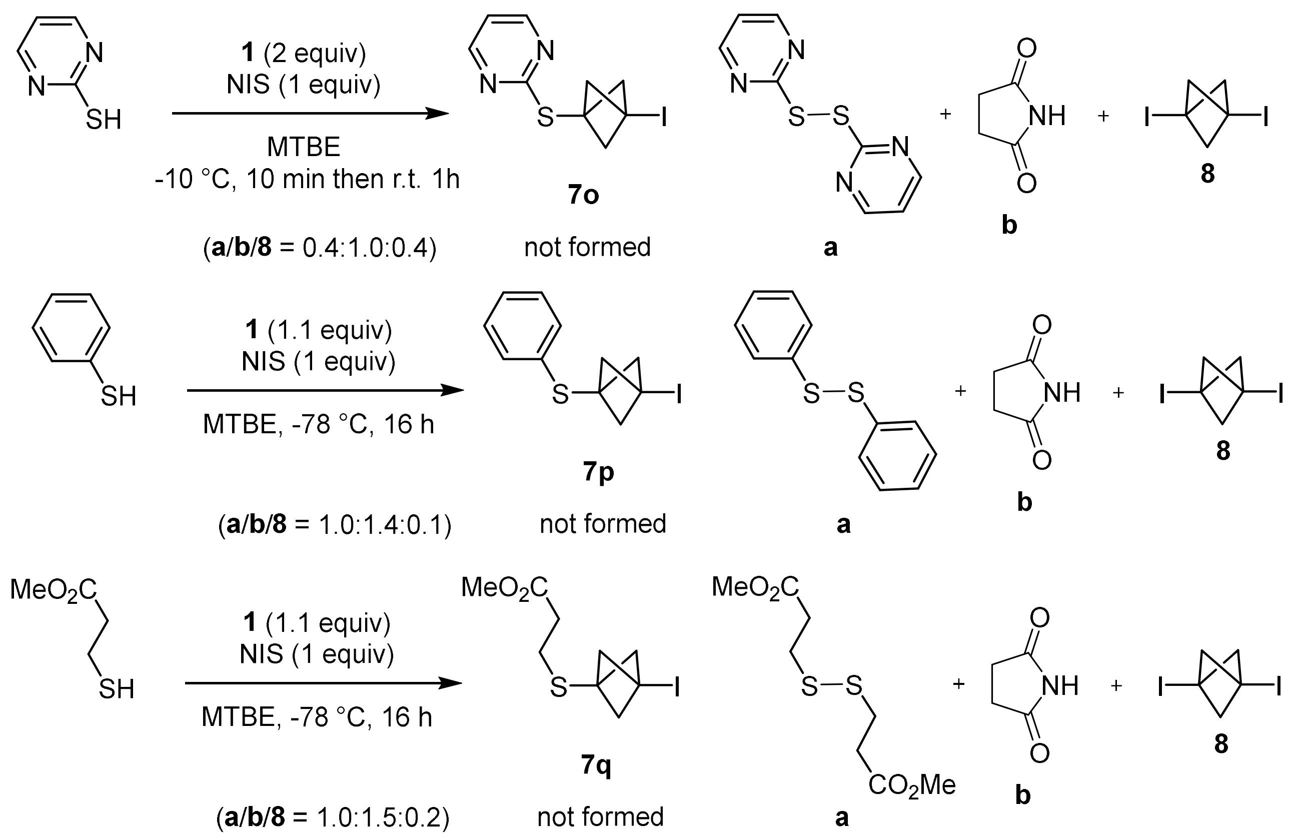

Functional group tolerance

Representative procedure for the iodo-sulfenylation of [1.1.1]propellane in the presence of nucleophilic additive. A test tube equipped with a stirring bar was charged under air with the 2-mercaptobenzothiazole (0.15 mmol, 25 mg, 1.0 equiv), one of **9**–**16** (0.15 mmol, 1 equiv) and MTBE (0.75 mL, 0.2 M), then cooled to -78 °C in a cryogenic bath fitted with a cold finger. [1.1.1]Propellane (**1**) (0.165 mmol, 0.28 mL, 1.1 equiv, 0.60 M stock solution in Et_2O) was then added followed by NIS (34 mg, 0.15 mmol, 1.0 equiv). The reaction vessel was sealed with a septum and the mixture was stirred at -78 °C for 16 h. The tube was then taken out of the cold bath and its content transferred to a round-bottom flask by rinsing with CH_2Cl_2 twice. Silica was added and the volatiles were removed *in vacuo*. The crude reaction mixture thus loaded on silica was purified by flash column chromatography on silica gel (EtOAc/hexanes = 2:98) to give **7a** in the amounts specified below. Note, all of **9**–**16** were purchased, except **14** (prepared as described on the next page).

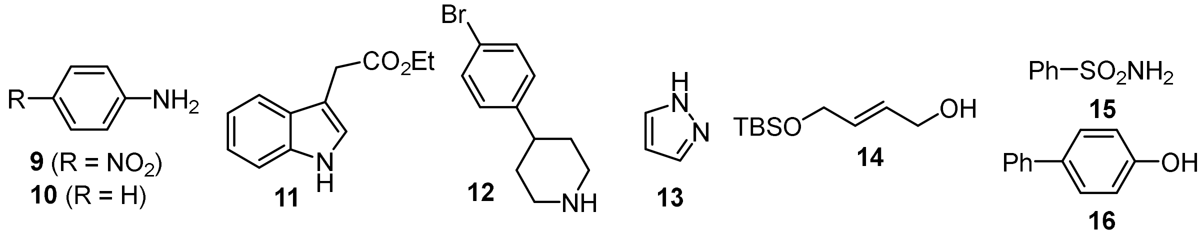

nucleophile	**7a** (isolated)	nucleophile recovered	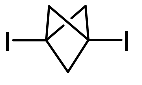 **8**
**9** (25 mg)	46 mg, 85%	19 mg, 90%	trace
**10** (14 µL)	13 mg, 24%	not attempted	15 mg, 31%
**11** (30 mg)	34 mg, 63%	30 mg, 100%	trace
**12** (36 mg)	20 mg, 37%	not attempted	**7a**/**8** = 4:1 (^{1}H NMR)
**13** (10.2 mg)	24 mg, 45%	not attempted	10.7 mg, 23%
**14** (30 mg)	46 mg, 85%	25 mg, 82%	trace
**15** (24 mg)	42 mg, 78%	not attempted	trace
**16** (26 mg)	41 mg, 76 %	24 mg, 94%	trace

***(E)*-4-((tert-butyldimethylsilyl)oxy)but-2-en-1-ol (14)**. A solution of but-2-yne-1,4-diol (1.50 g, 17.4 mmol, 1 equiv) in THF (20 mL) was added under argon to a suspension of $LiAlH_4$ (800 mg, 21 mmol, 1.2 equiv) in THF (35 mL) at 0 °C. After heating at reflux for 12 h, the mixture was cooled to 0 °C and quenched by the sequential addition of water (2 mL), a 15% aqueous solution of NaOH (2 mL) and additional water (6 mL). The mixture was extracted with ether (3 × 15 mL), and the combined organic layers were then washed with brine and dried over $MgSO_4$, filtered and concentrated under vacuum. The crude material thus obtained (1.45 g, 94%) was diluted in THF (9 mL) under argon and added dropwise to a suspension of NaH (60% in mineral oil, 760 mg, 19.2 mmol, 1.1 equiv) in THF (38 mL) at 0 °C. After stirring at room temperature for 1 h, TBDMSCl (2.03 g, 19.2 mmol, 1.1 equiv) in THF (7 mL) was added slowly over 10 min. After stirring at room temperature for 12 h, the reaction mixture was quenched with an aqueous saturated NH_4Cl solution. The organic layer was separated and the aqueous layer was extracted with diethyl ether (3 × 10 mL). The combined organic layers were washed with brine (30 mL), dried over $MgSO_4$ and the solvent was removed under vacuum. The crude residue was purified by silica gel column chromatography (10 to 20% EtOAc in hexane) to afford mono-protected alcohol **14** (1.88 g, 27%). ^1H NMR (500 MHz, $CDCl_3$): δ 5.92–5.84 (m, 1H), 5.83–5.76 (m, 1H), 4.21–4.18 (m, 2H), 4.18–4.14 (m, 2H), 0.91 (s, 9H), 0.08 (s, 6H); the data is in agreement with the literature.[5]

Control reactions

1. Reactions of 2-mercapto azoles with propellane (**1**) in the absence of NIS

Following the procedure reported by Bräse,[3] a solution of thiol **2** (0.2 mmol, 33 mg, 1 equiv) or **3** (0.2 mmol, 24 mg, 1 equiv) in Et_2O (0.5 mL) under argon was added via canula to a solution of [1.1.1]propellane (0.2 mmol, 0.17 mL, 1 equiv, 0.85 M in Et_2O). After stirring under argon for 15 minutes at room temperature, TLC indicated no conversion. The TLC remained unchanged after 1 h stirring.

2. Sequential reaction of 2-mercaptobenzothiazole (**2**) with NIS and propellane (**1**)

2-Mercaptobenzothiazole **2** (0.2 mmol, 33 mg, 1 equiv) was dissolved in MTBE (1 mL) and cooled to at -78 °C before adding N-iodosuccinimide (0.2 mmol, 45 mg, 1 equiv). After stirring for 16 h, evaporation of the pink solution to dryness and ^{1}H NMR with CH_2Cl_2 as internal standard showed quantitative conversion to disulfide **17** {^{1}H NMR (500 MHz, $CDCl_3$): δ 7.95 (d, J = 7.9 Hz, 2H), 7.78 (d, J = 7.9 Hz, 2H), 7.47 (t, J = 7.3 Hz, 2H), 7.36 (t, J = 7.6 Hz, 2H); HRMS (ESI$^+$): *m/z* calculated for $C_{14}H_8N_2S_4$ $[M+H]^+$: 332.9643, found: 332.9639}; the data is in agreement with the literature.[6] The reaction was repeated and TLC indicated complete conversion of **2** into **17**, at which point [1.1.1]propellane (0.3 mL, 0.22 mmol, 0.76 M in Et_2O) was added and the reaction mixture was stirred at -78 °C for an additional 16 hours. Evaporation of the crude to dryness and ^{1}H NMR with CH_2Cl_2 as internal standard showed the formation of 1,3-bisiodobicyclo[1.1.1]pentane (**8**) in 45% besides 50% of **17** remaining. Compound **7a** is absent from this crude mixture.

3. Reactions in the presence of radical inhibitors.

The representative procedure for the formation of **7a** was conducted on **2** (0.2 mmol, 33 mg) in the presence a radical inhibitor (either BHT (0.2 mmol, 44 mg, 1 equiv), TEMPO (0.2 mmol, 31 mg, 1 equiv), or TEMPO (1.0 mmol, 156 mg, 5 equiv) which was added before NIS (0.2 mmol, 45 mg, 1 equiv). After 16 h stirring at -78 °C, the crude was evaporated to dryness and the yields were determined by ^{1}H NMR using CH_2Cl_2 as internal standard (99%, 86%, and 80%, respectively).

4. Reaction of 1,3-bisiodobicyclo[1.1.1]pentane (**8**) with 2-mercaptobenzothiazole (**2**).

1,3-Bisiodobicyclo[1.1.1]pentane (**8**) (14 mg, 0.044 mmol, 1 equiv) was dissolved in MTBE (0.5 mL) at -78 °C before adding 2-mercaptobenzothiazole (**2**) (7 mg, 0.044 mmol, 1 equiv). After stirring for 16 h, TLC (ethyl acetate/hexanes = 1:4) showed no conversion of the starting materials and the absence of compound **7a**.

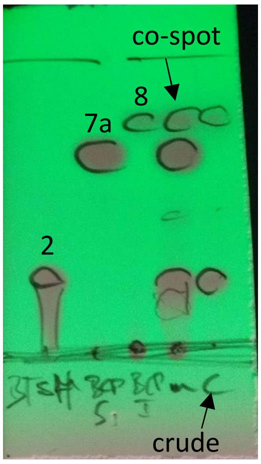

Functionalisation of the C–I bond

2-(Bicyclo[1.1.1]pentan-1-ylthio)-4,5-dihydrothiazole (18). A test tube was charged with 2-((3-iodobicyclo[1.1.1]pentan-1-yl)thio)-4,5-dihydrothiazole (**7g**) (62 mg, 0.2 mmol, 1.0 equiv) and THF (1 mL, 0.2 M) under air. Then, 2-mercaptoethanol (14 µL, 0.2 mmol, 1.0 equiv), tri-*n*-butyl tinhydride (86 µL, 0.32 mmol, 1.6 equiv) and triethyl borane (40 µL, 0.04 mmol, 0.2 equiv, 1.0 M in hexanes) were added in succession and 2 mL of air was slowly bubbled through the solution. The mixture was stirred at room temperature for 1 hour before KF (0.75 mL, 1.7 M in methanol) was added and the mixture was stirred for an additional 3 hours. The volatiles were removed *in vacuo* and the crude salts were thoroughly triturated with pentane (50 mL) and filtered. The pentane was collected and the volatiles removed *in vacuo* to yield a white solid which was triturated further with dichloromethane (25 mL). Following filtration, the dichloromethane was removed *in vacuo* and the crude oil thus obtained was loaded directly onto a column packed with 1:9 K_2CO_3/SiO_2. Purification by flash chromatography (hexanes only to hexanes/ethyl acetate (95:5)) gave 2-(bicyclo[1.1.1]pentan-1-ylthio)-4,5-dihydrothiazole (**18**) (33 mg, 89%). Colorless oil. ^{1}H NMR (500 MHz, $CDCl_3$): δ 4.23 (t, J = 8.2 Hz, 2H), 3.37 (t, J = 8.2 Hz, 2H), 2.82 (s, 1H), 2.24 (s, 6H). $^{13}C\{^1H\}$ NMR (125 MHz, $CDCl_3$): δ {168.9}, 62.6, 55.0 (3C), 43.1, 34.3, 30.5; the resonance indicated in bracket is not visible but is inferred from HMBC. IR (neat): $\tilde{\nu}$ = 2980 (m), 2966 (m), 2913 (m), 2879 (m), 2849 (m), 1567 (s), 1504 (w), 1448 (m), 1433 (m), 1303 (m), 1259 (m), 1204 (s), 1125 (m), 1075 (m), 1020 (m), 992 (m), 961 (s), 916 (m), 889 (s), 800 (m), 775 (m), 745 (w), 711 (w), 692 (w), 658 (w), 636 (m), 621 (w), 608 (w). HRMS (ESI$^+$) *m/z* calculated for $C_8H_{11}NS_2$ [M+H]$^+$: 186.0406; found: 186.0405.

Methyl 3-(3-((4,5-dihydrothiazol-2-yl)thio)bicyclo[1.1.1]pentan-1-yl)propanoate (19). A Schlenk tube (standard borosilicate) was charged with 2-((3-iodobicyclo[1.1.1]pentan-1-yl)thio)-4,5-dihydrothiazole (**7g**) (187 mg, 0.6 mmol, 1.0 equiv), Na_2CO_3 (127 mg, 1.2 mmol, 2.0 equiv), and Ir[(dF(CF_3)ppy)$_2$(dtbbpy)]PF_6 (16.8 mg, 0.0015 mmol, 2.5 mol%). The tube was evacuated and refilled with argon three times. Then, MeOH (3.6 mL), water (0.4 mL), methyl acrylate (0.16 mL, 1.8 mmol, 3.0 equiv), and $(Me_3Si)_3SiH$ (0.37 mL, 1.2 mmol, 2.0 equiv) were added under argon. The mixture was degassed by bubbling argon through the suspension for 3 minutes. The reaction mixture was stirred under argon and irradiation by two Kessil® blue

LED lamps (Tuna-blue, A160WE, 456 nm, 40W, set at full intensity and positioned 4.5 cm away from the reaction vessel at 180° from each other with a desk fan-cooling from the side to keep the ambient temperature below 30 °C, see Figure S2) for 24 hours. The mixture was filtered through cotton wool and concentrated *in vacuo*. The crude material was diluted in water and extracted with CH_2Cl_2 (3 x 10 mL), and the combined organic layers were dried over $MgSO_4$, filtered and concentrated. A first purification by flash chromatography over silica (hexanes only then hexanes/ethyl acetate (95:5 to 90:10)) gave 82 mg of the desired material that was still contaminated by silicon-containing by-products. Hence, further purification of that material by preparative thin layer chromatography (hexanes/ethyl acetate (75:25), one elution) gave **18** in pure form (45 mg, 28%). Colorless oil. ^{1}H NMR (400 MHz, $CDCl_3$): δ 4.20 (t, J = 8.0 Hz, 2H), 3.66 (s, 3H), 3.36–3.28 (m, 2H), 2.28 (t, J = 7.5 Hz, 2H), 2.04 (s, 6H), 1.87 (t, J = 7.5 Hz, 2H). ^{13}C{^{1}H} NMR (100 MHz, $CDCl_3$): δ 173.5, {164.5}, 64.1, 54.7 (3C), 51.6, 42.2, 39.4, 34.5, 31.2; the resonance indicated in bracket is not visible but is inferred from HMBC. IR (neat): $\tilde{\nu}$ = 2950 (w), 2913 (w), 2876 (w), 1735 (s), 1568 (m), 1436 (m), 1356 (w), 1319 (w), 1305 (w), 1246 (m), 1189 (s), 1172 (s), 1056 (m), 992 (m), 989 (s), 918 (m), 886 (m), 835 (s). HRMS (ESI$^+$) *m/z* calculated for $C_{12}H_{17}NO_2S_2$ [M+H]$^+$: 272.0773; found: 272.0773.

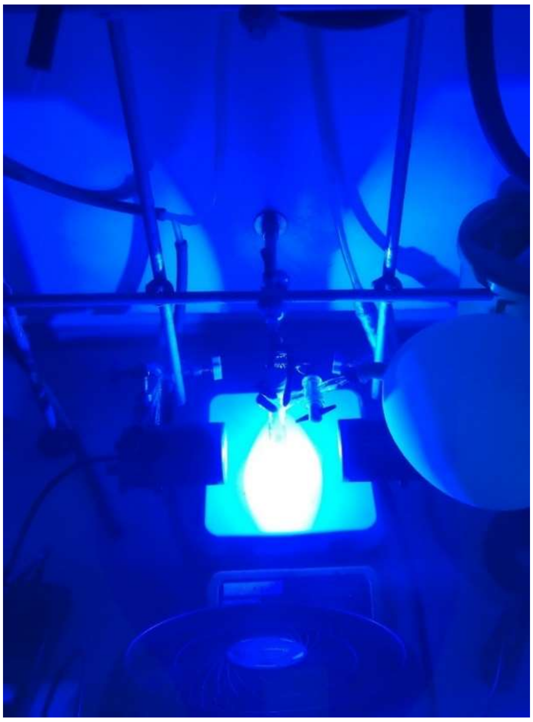

Figure S2. Set-up for the blue-LED mediated Giese reaction of **7k** to **19.**

References

1. J. M. Lopchuk, K. Fjelbye, Y. Kawamata, L. R. Malins, C. M. Pan, R. Gianatassio, J. Wang, L. Prieto, J. Bradow, T. A. Brandt, M. R. Collins, J. Elleraas, J. Ewanicki, W. Farrell, O. O. Fadeyi, G. M. Gallego, J. J. Mousseau, R. Oliver, N. W. Sach, J. K. Smith, J. E. Spangler, H. Zhu, J. Zhu and P. S. Baran, *J. Am. Chem. Soc.* **2017**, *139*, 3209.
2. Zarate, C.; Ardolino, M.; Morriello, G. J.; Logan, K. M.; Kaplan, W. P.; Torres, L.; Li, D.; Chen, M.; Li, H.; Su, J.; Fuller, P.; Maddess, M. L.; Song, Z. J. *Org. Process Res. Dev.* **2021**, *25*, 642.
3. Bär, R. M.; Kirschner, S.; Nieger, M.; Bräse, S. *Chem. – Eur. J.* **2018**, *24,* 1373.
4. Wiberg, K. B.; Waddell, S. T. *J. Am. Chem. Soc.* **1990**, *112*, 2194.
5. Wölfl, B.; Mata, G.; Fürstner, A. *Chem – Eur. J.* **2019**, *25*, 255.
6. Vandavasi, J. K.; Hu, W. P.; Chen, C. Y.; Wang, J. J. *Tetrahedron* **2011**, *67*, 8895

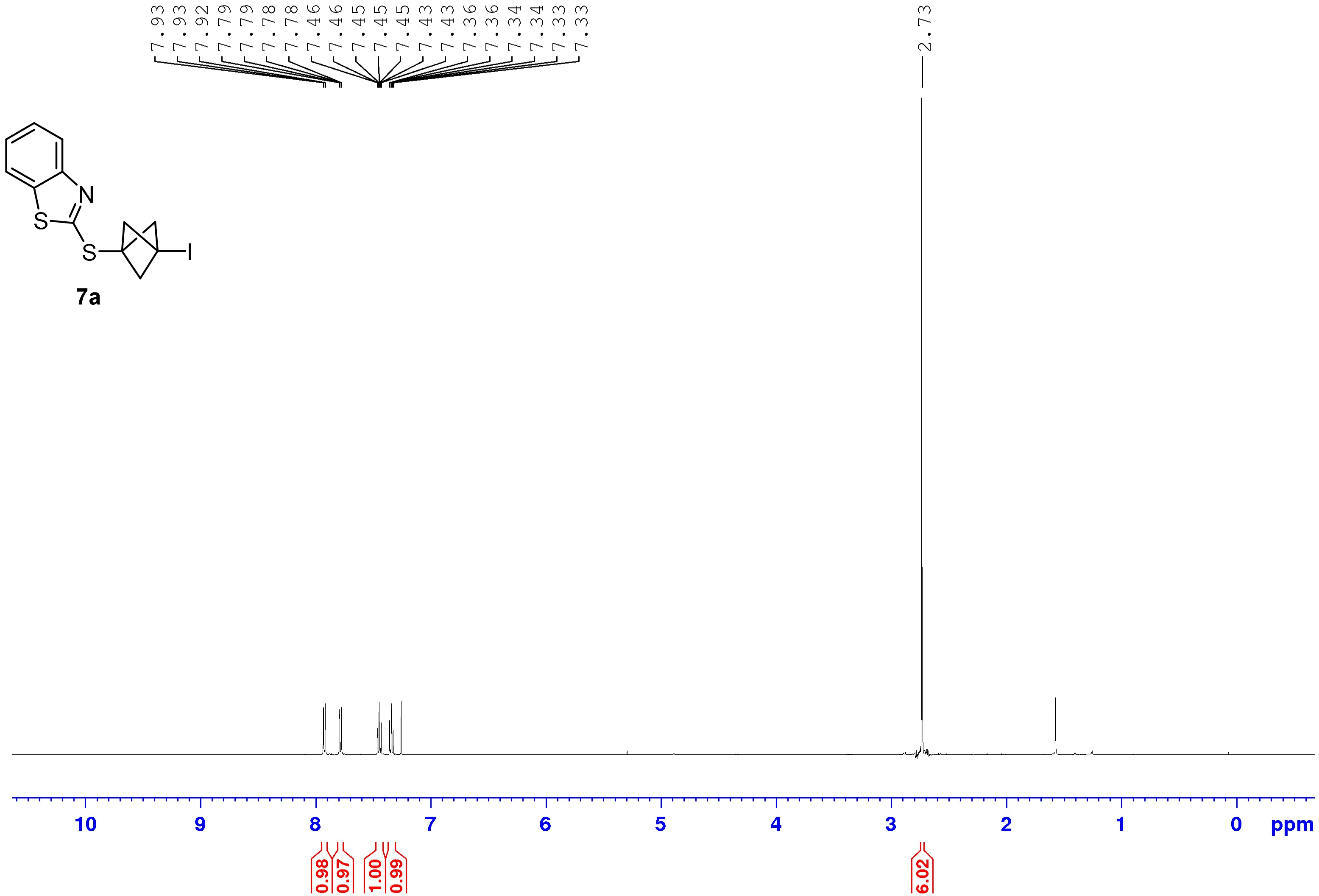

Figure S3. ^{1}H NMR (500 MHz, $CDCl_3$) of compound **7a**.

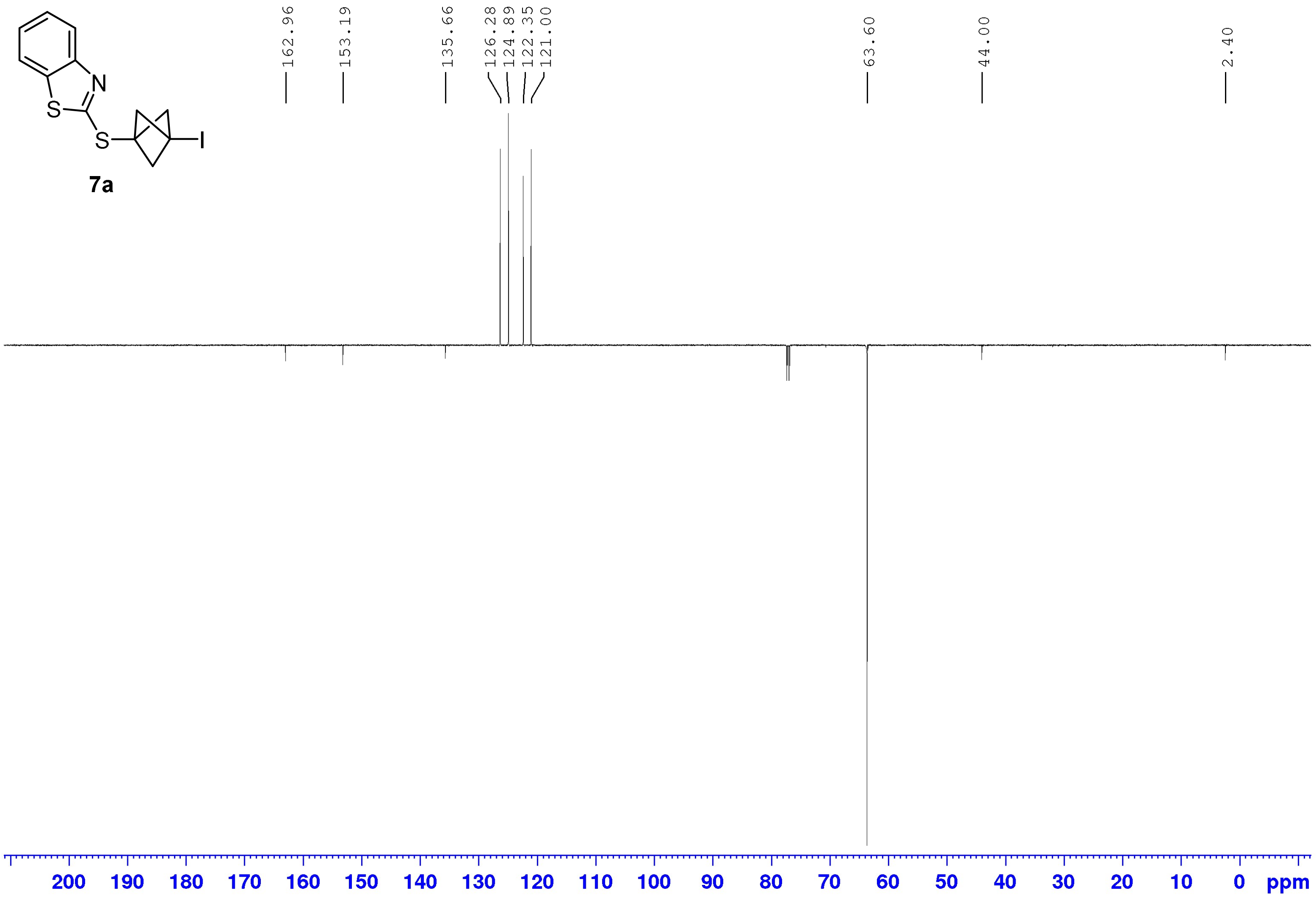

Figure S4. $^{13}C\{^{1}H\}$ NMR (126 MHz, $CDCl_3$) of compound **7a**.

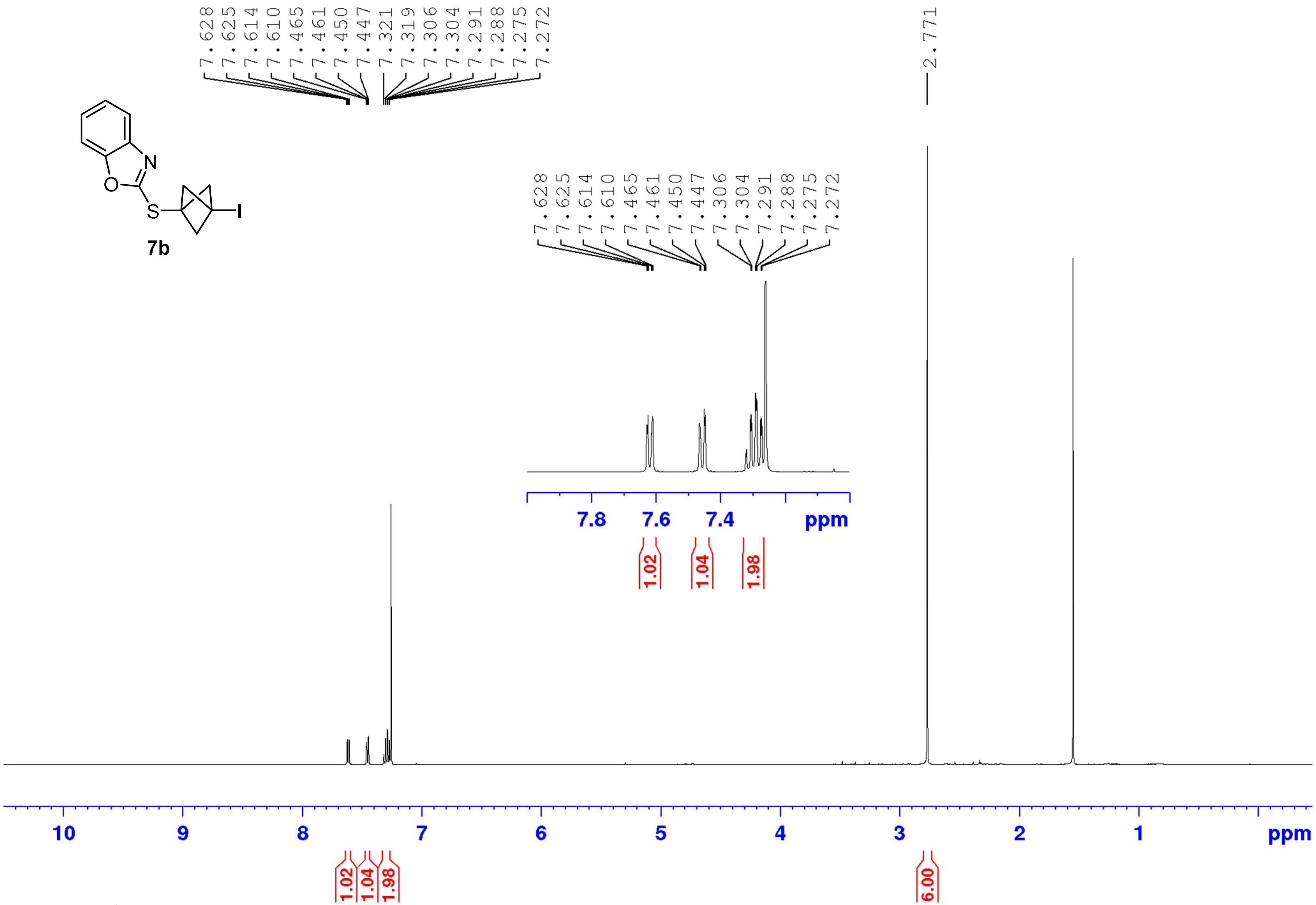

Figure S5. ^{1}H NMR (500 MHz, $CDCl_3$) of compound **7b**.

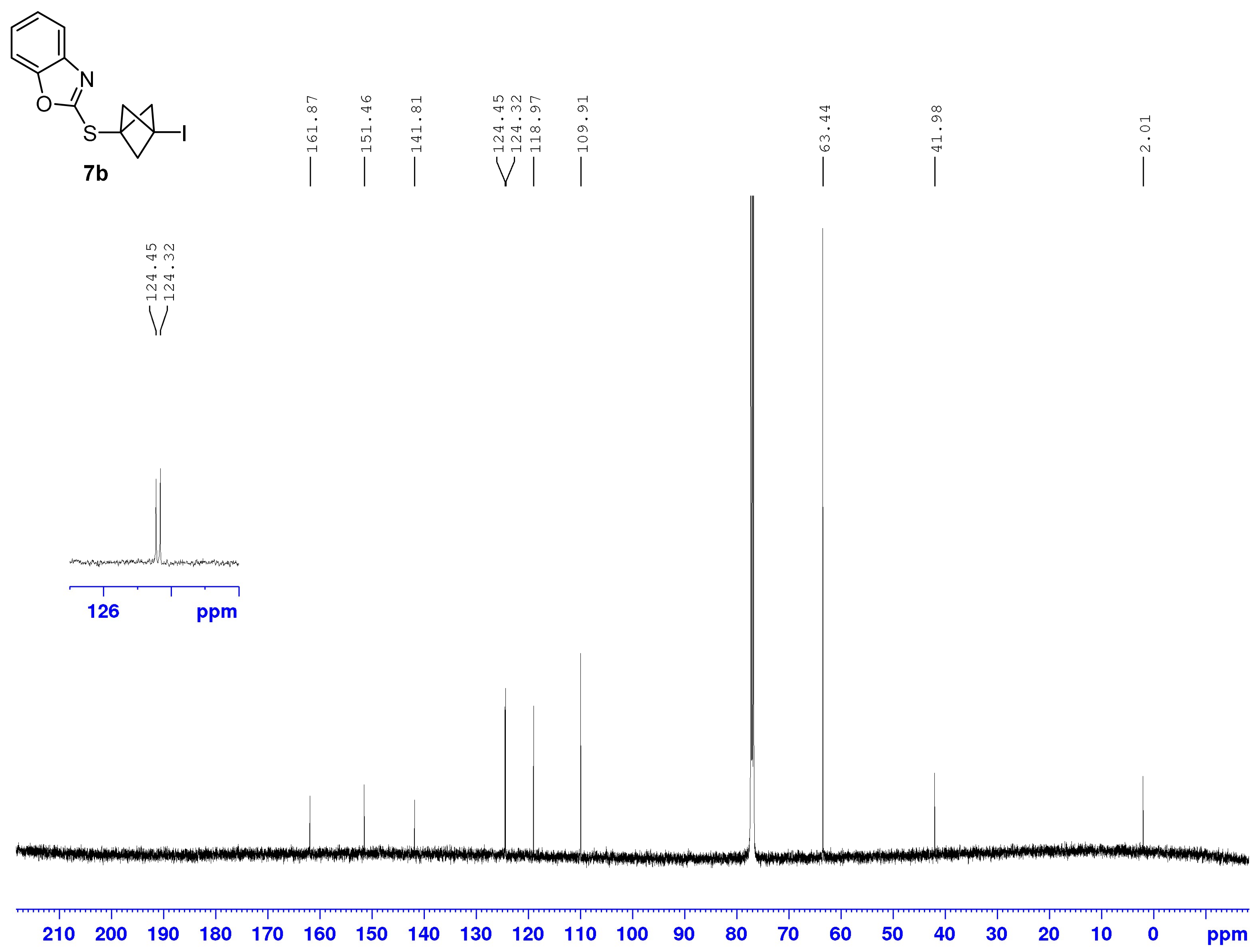

Figure S6. $^{13}C\{^{1}H\}$ NMR (126 MHz, $CDCl_3$) of compound **7b**.

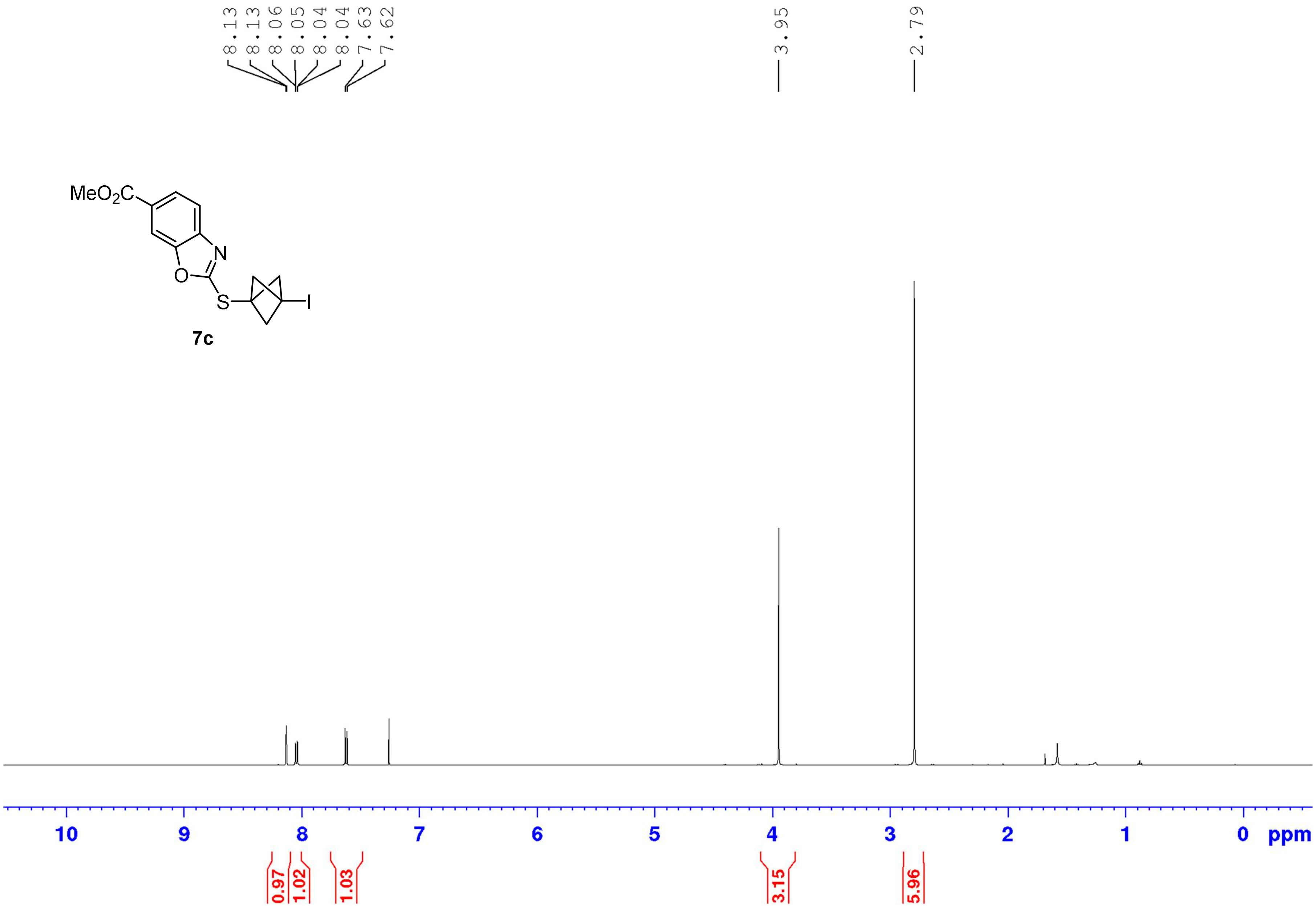

Figure S7. ^{1}H NMR (500 MHz, $CDCl_3$) of compound **7c**

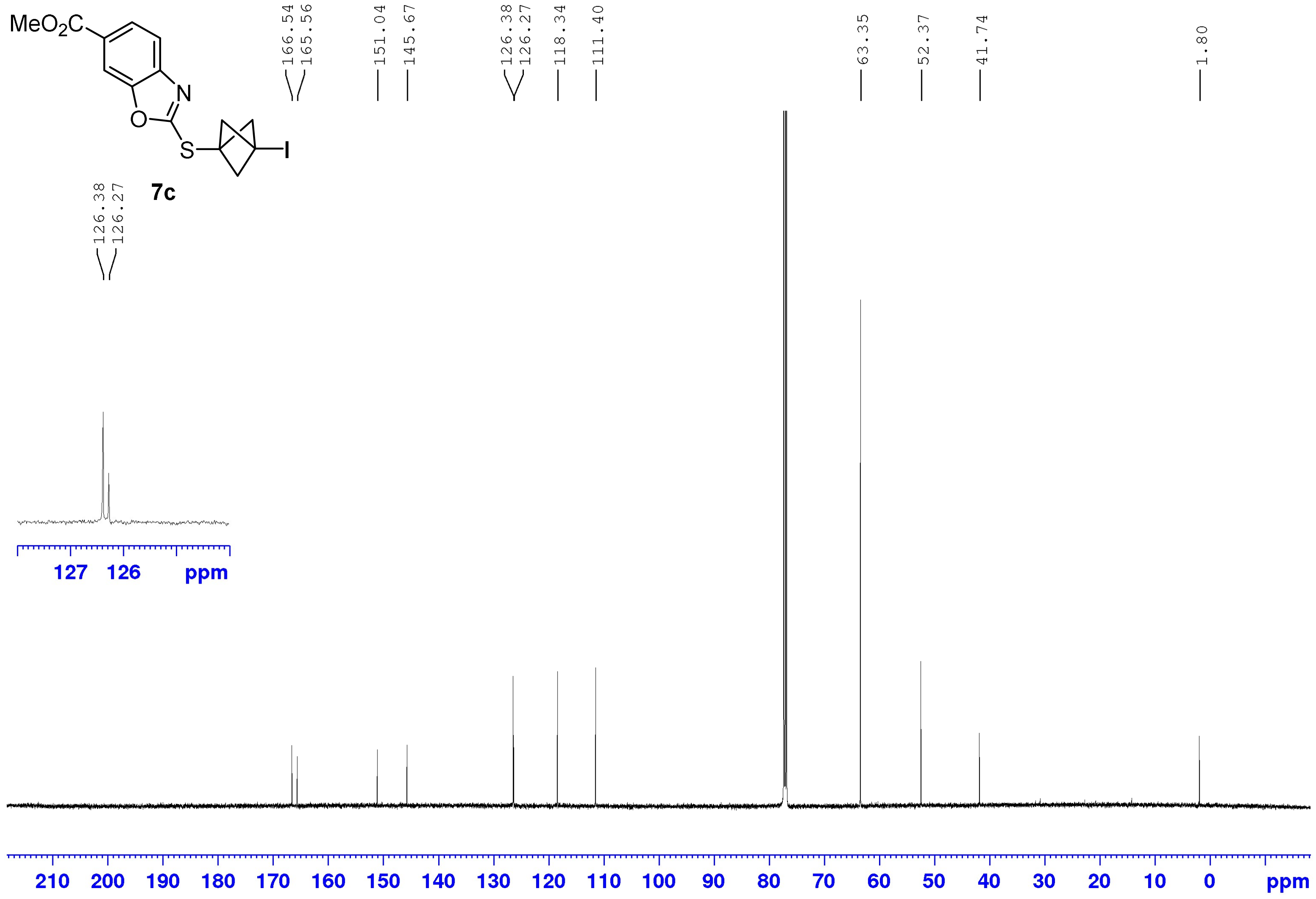

Figure S8. $^{13}C\{^{1}H\}$ NMR (126 MHz, $CDCl_3$) of compound **7c**

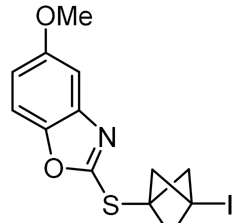

7d

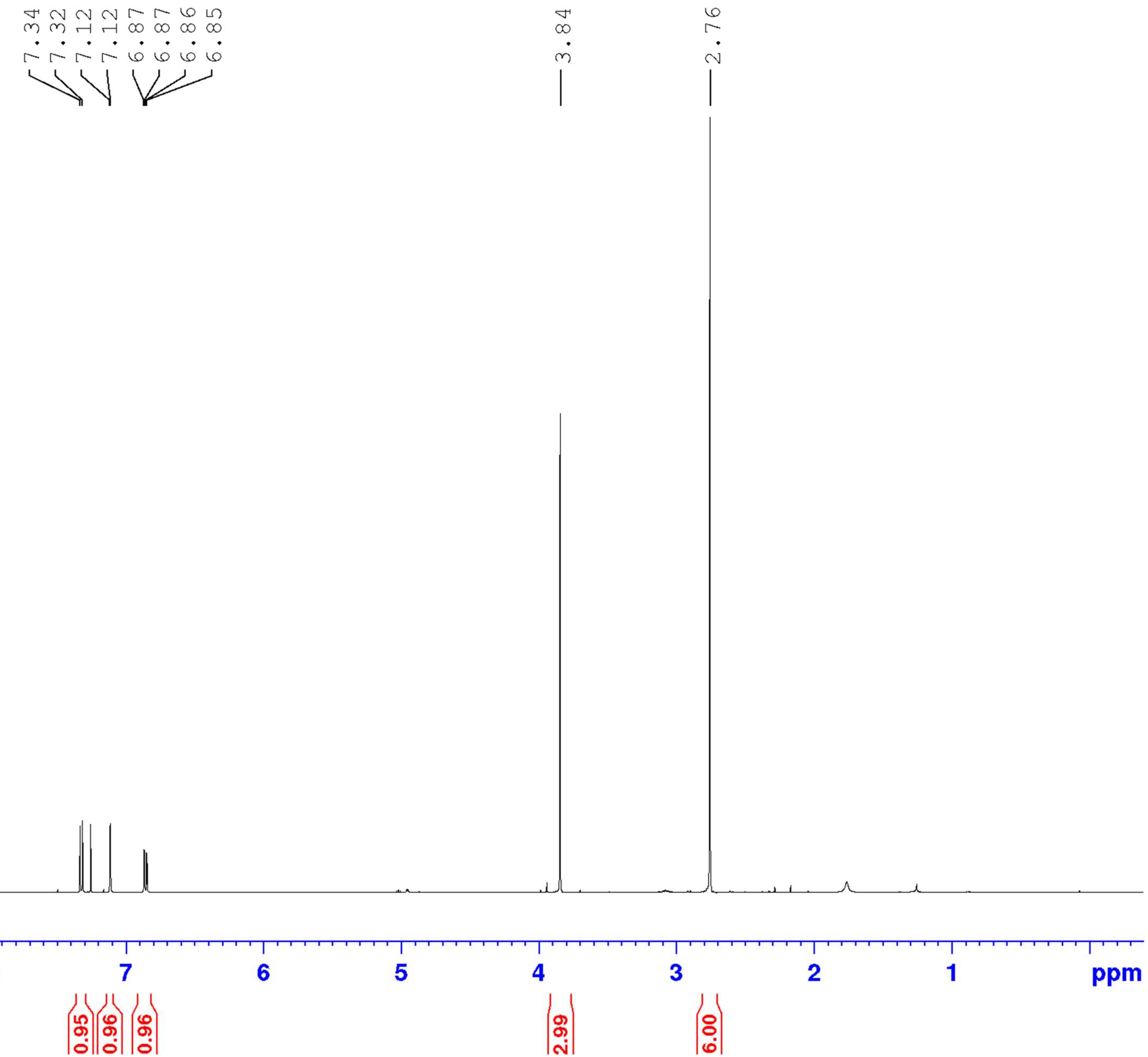

Figure S9. ^{1}H NMR (500 MHz, $CDCl_3$) of compound **7d**.

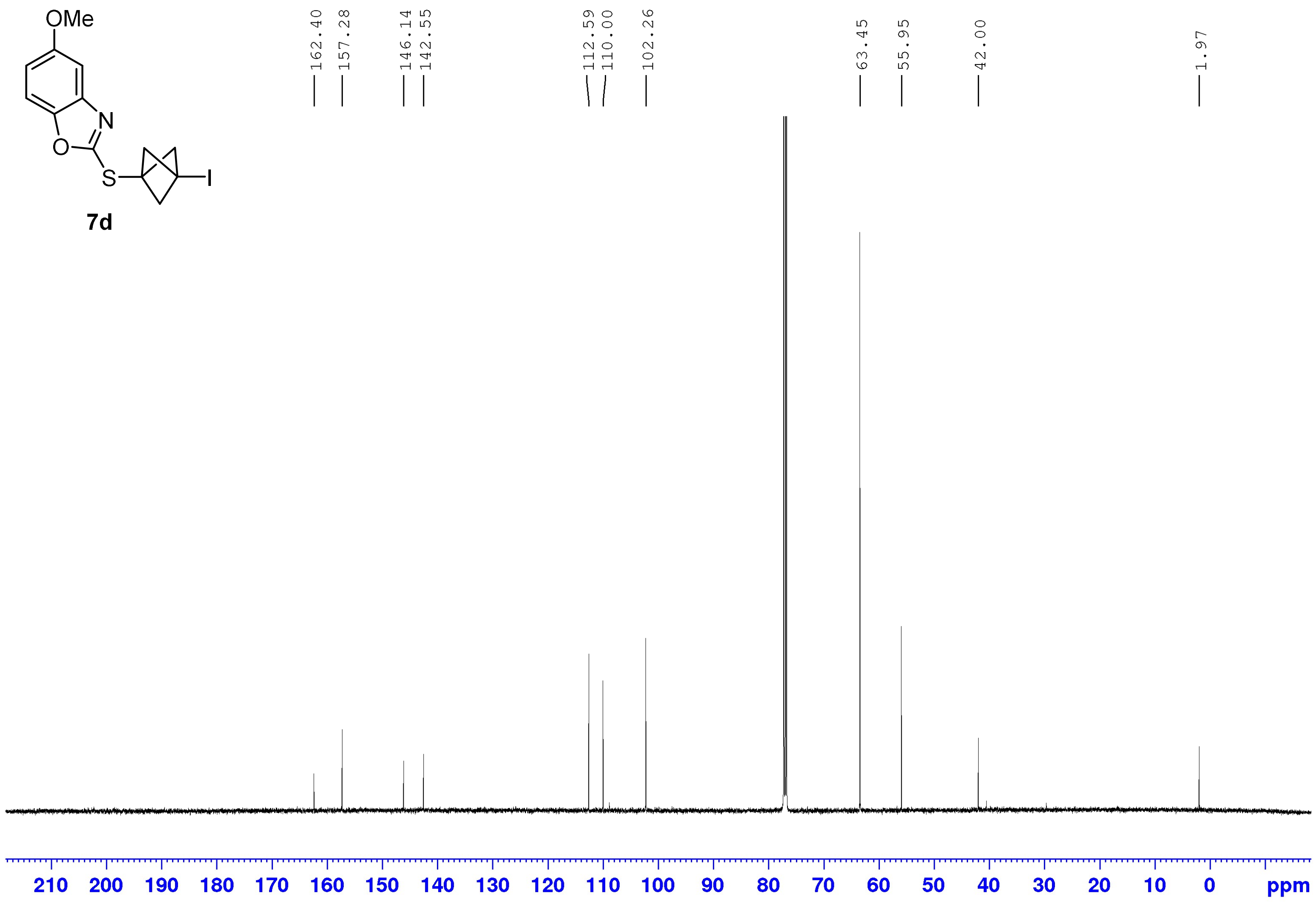

Figure S10. $^{13}C\{^{1}H\}$ NMR (126 MHz, $CDCl_3$) of compound **7d**

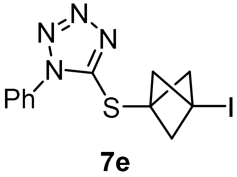

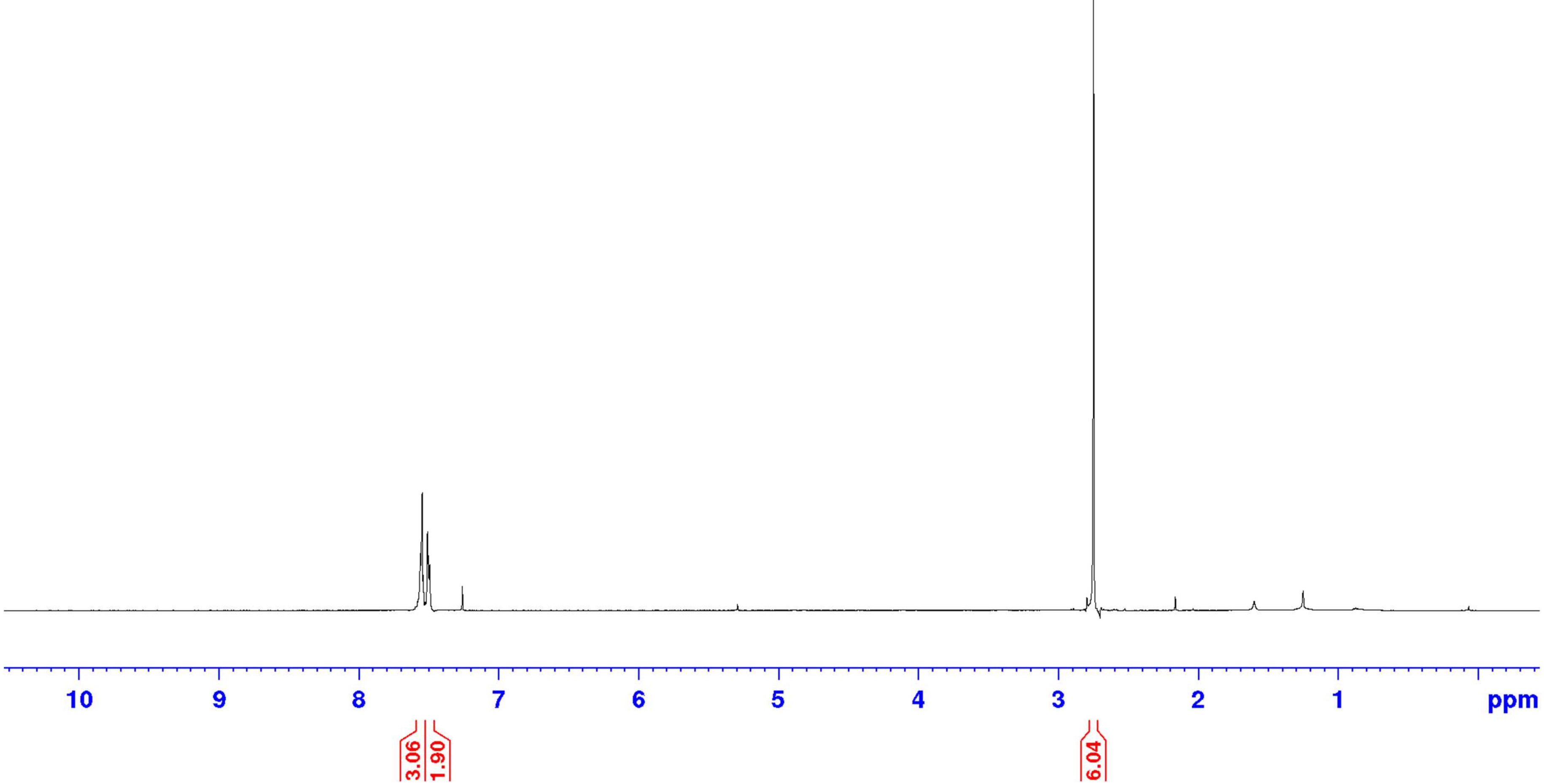

Figure S11. ^{1}H NMR (500 MHz, $CDCl_3$) of compound **7e**

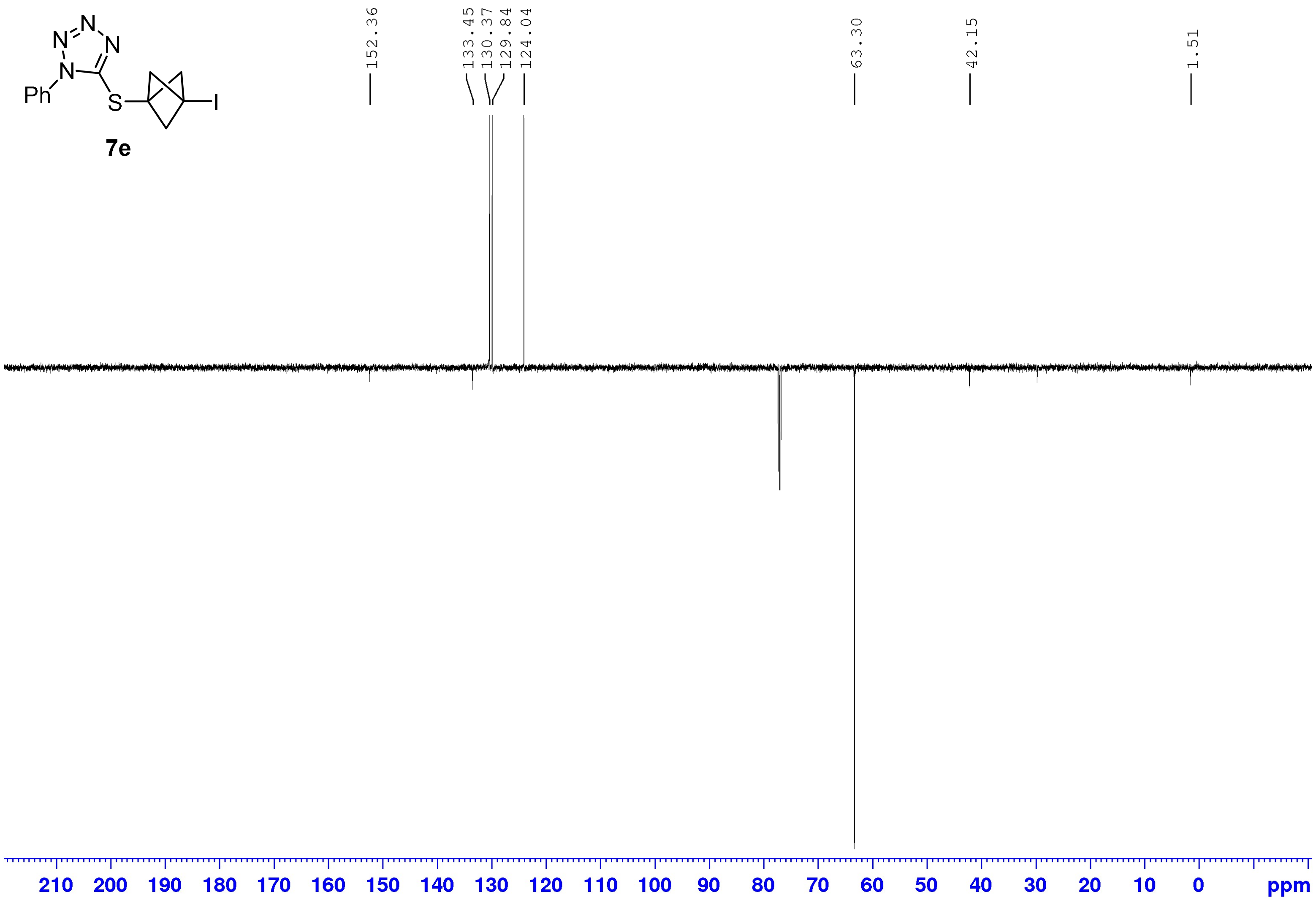

Figure S12. $^{13}C\{^{1}H\}$ NMR (126 MHz, $CDCl_3$) of compound **7e**

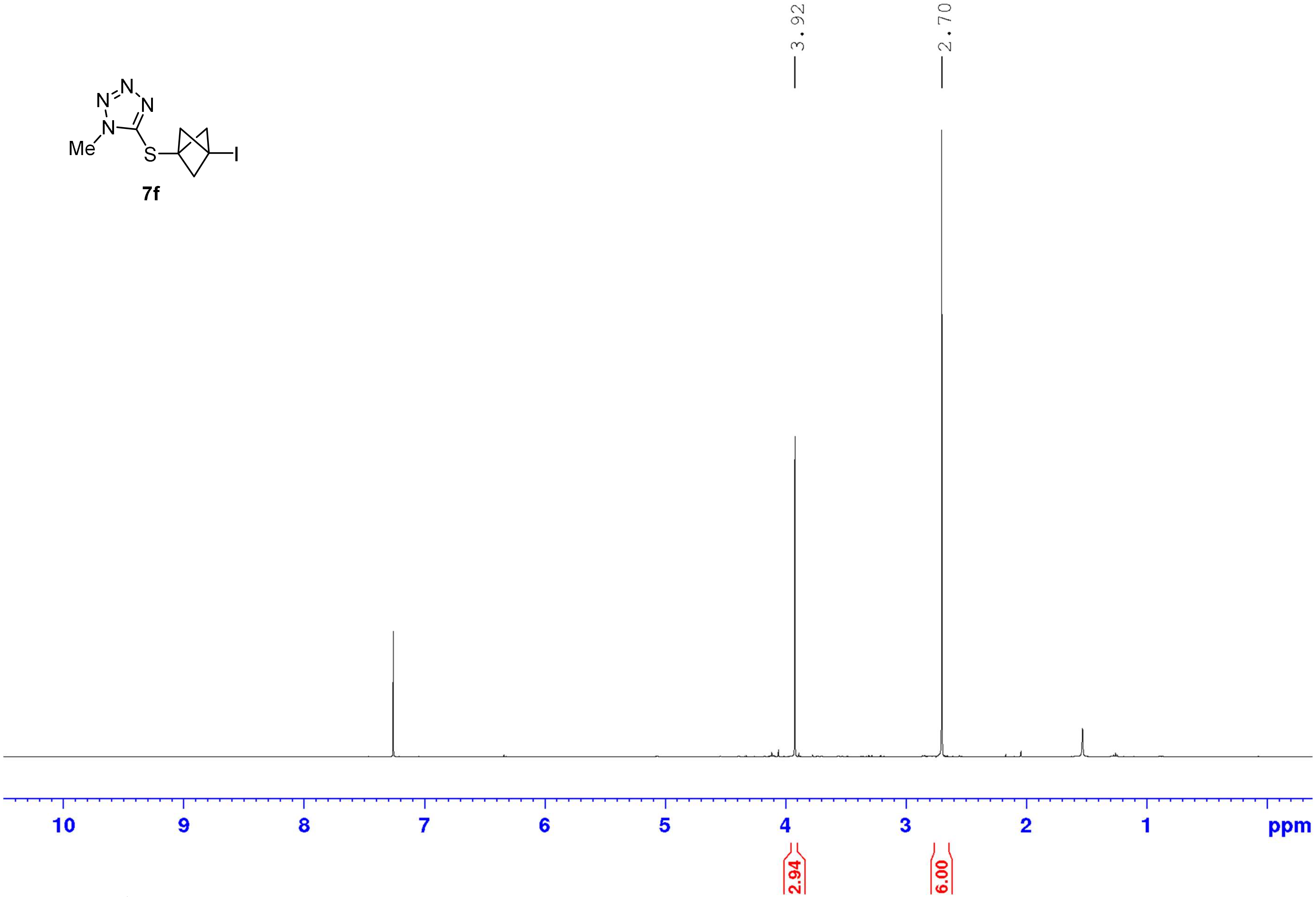

Figure S13. ^{1}H NMR (500 MHz, $CDCl_3$) of compound **7f**

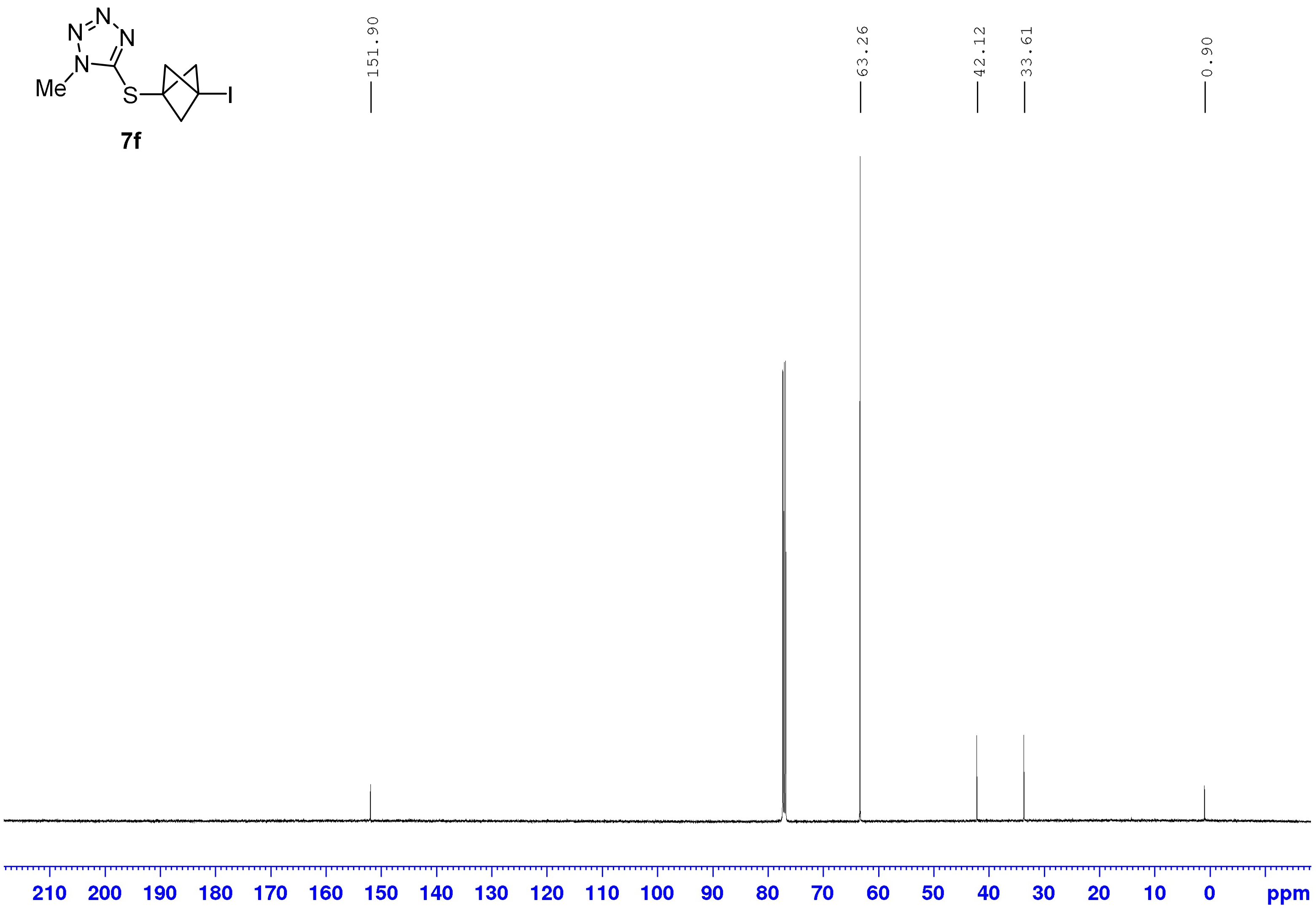

Figure S14. $^{13}C\{^1H\}$ NMR (126 MHz, $CDCl_3$) of compound **7f**

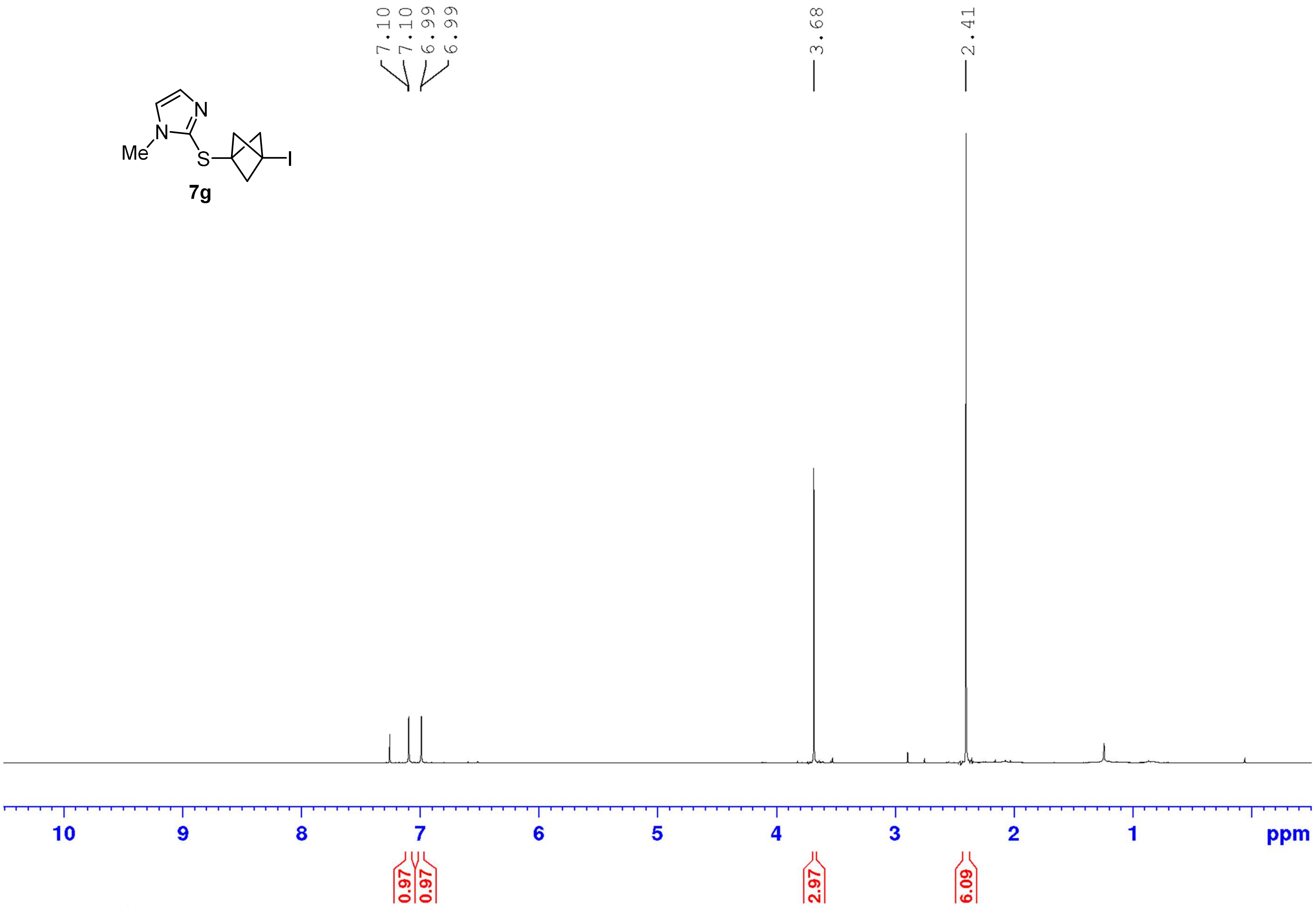

7g

Figure S15. ^{1}H NMR (500 MHz, $CDCl_3$) of compound **7g**

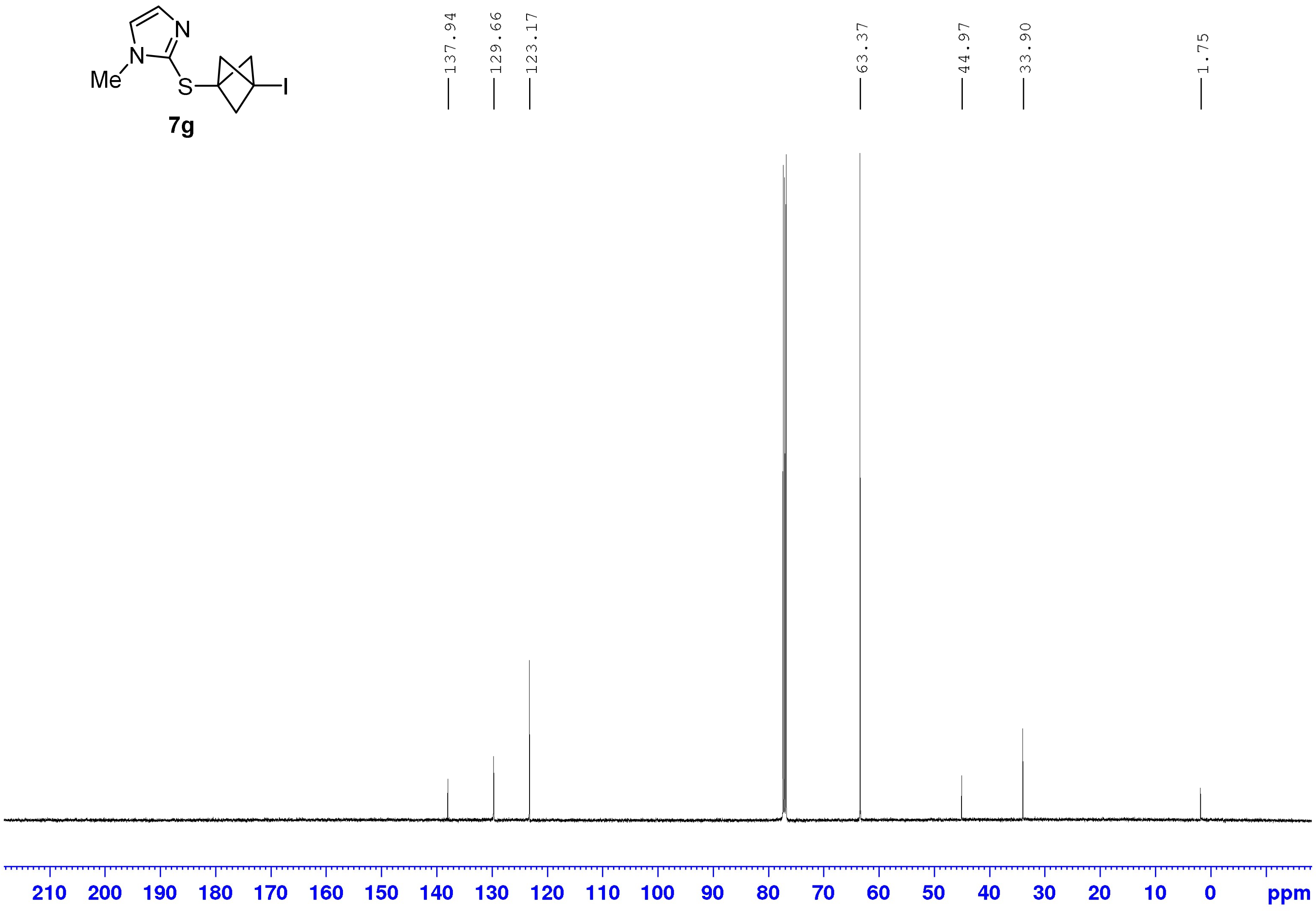

Figure S16. $^{13}C\{^{1}H\}$ NMR (126 MHz, $CDCl_3$) of compound **7g**

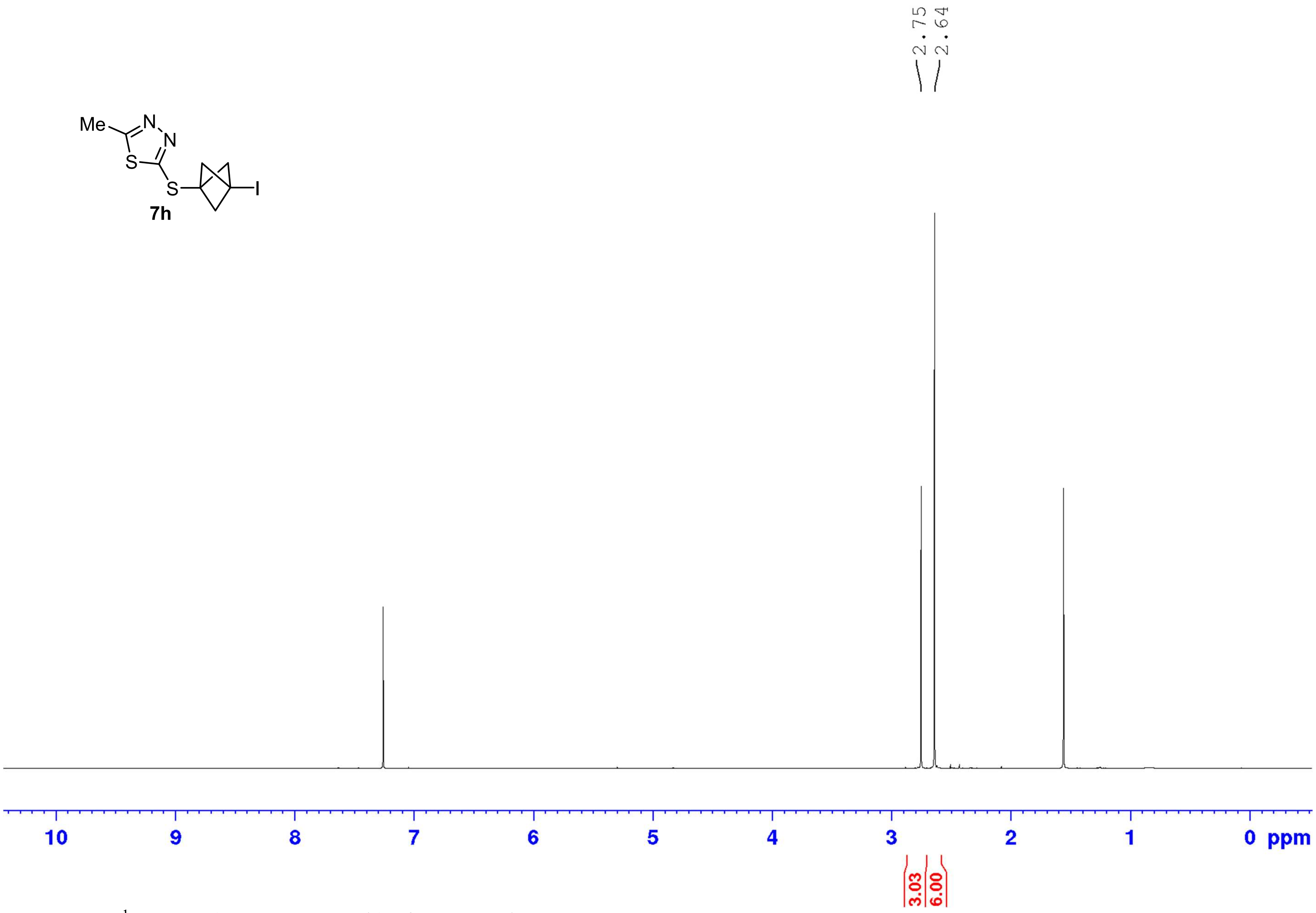

Figure S17. ^{1}H NMR (500 MHz, $CDCl_3$) of compound **7h**

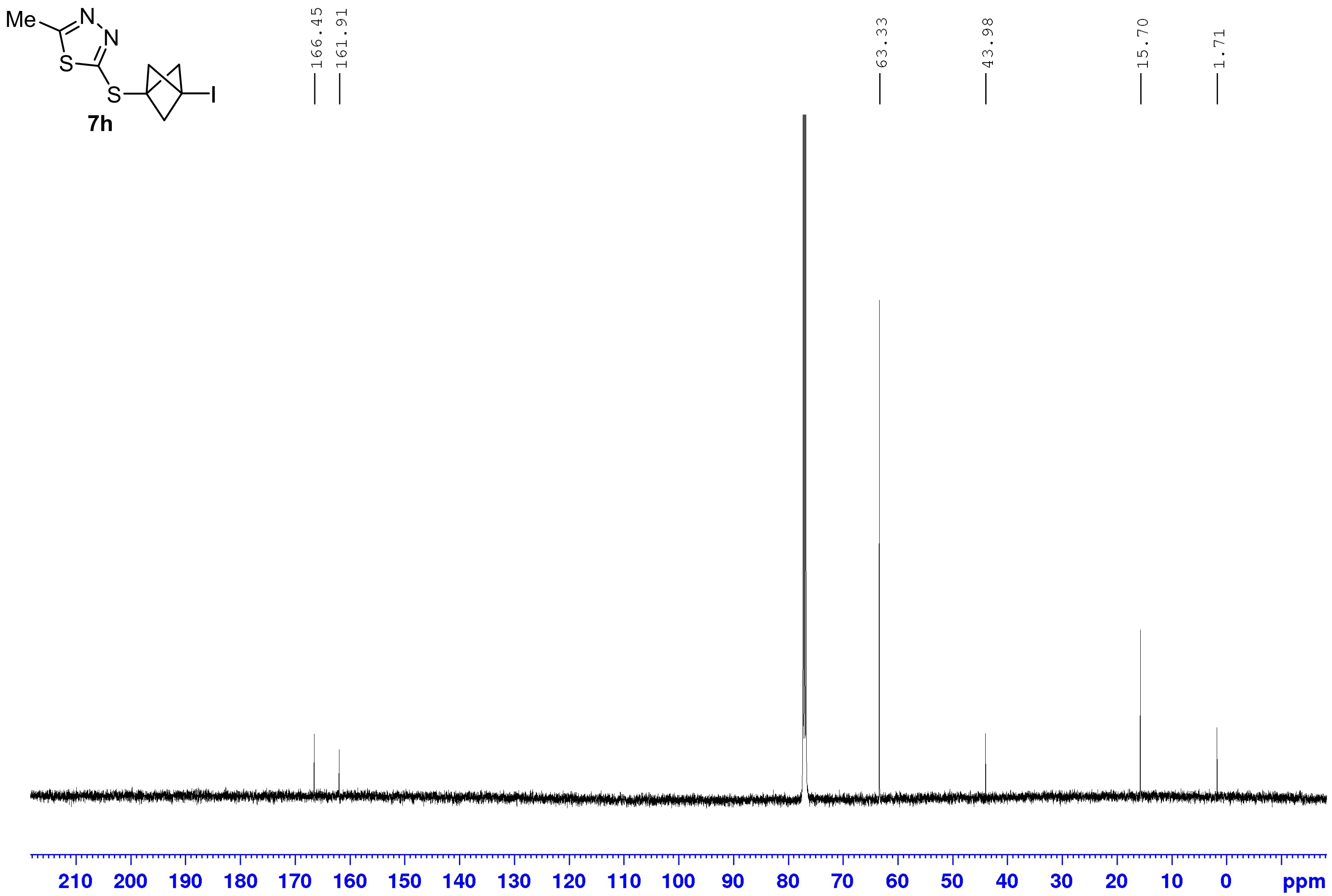

Figure S18. $^{13}C\{^1H\}$ NMR (126 MHz, $CDCl_3$) of compound **7h**

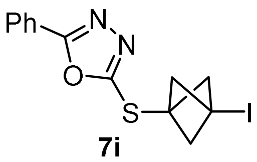

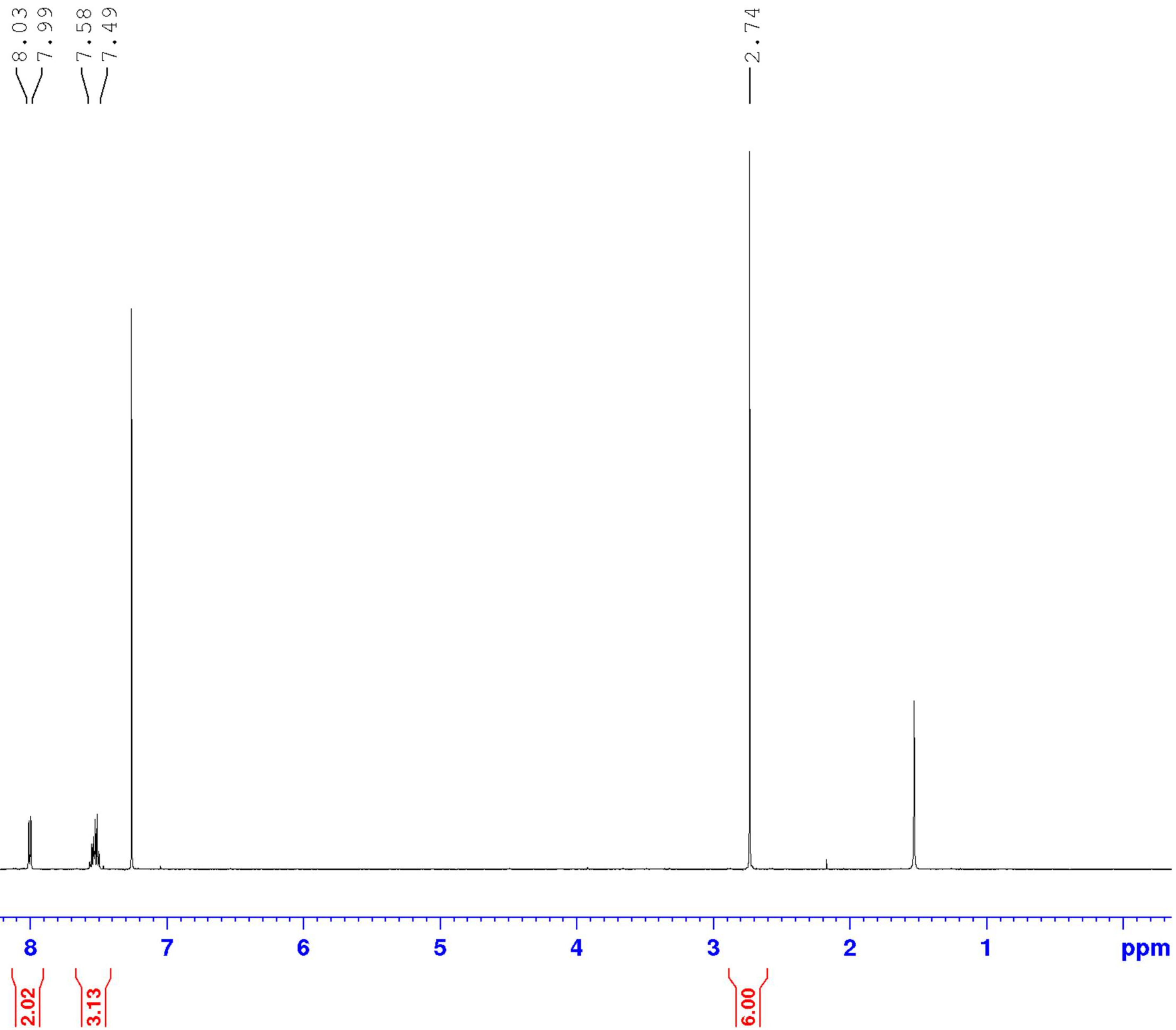

Figure S19. ^{1}H NMR (500 MHz, $CDCl_3$) of compound **7i**

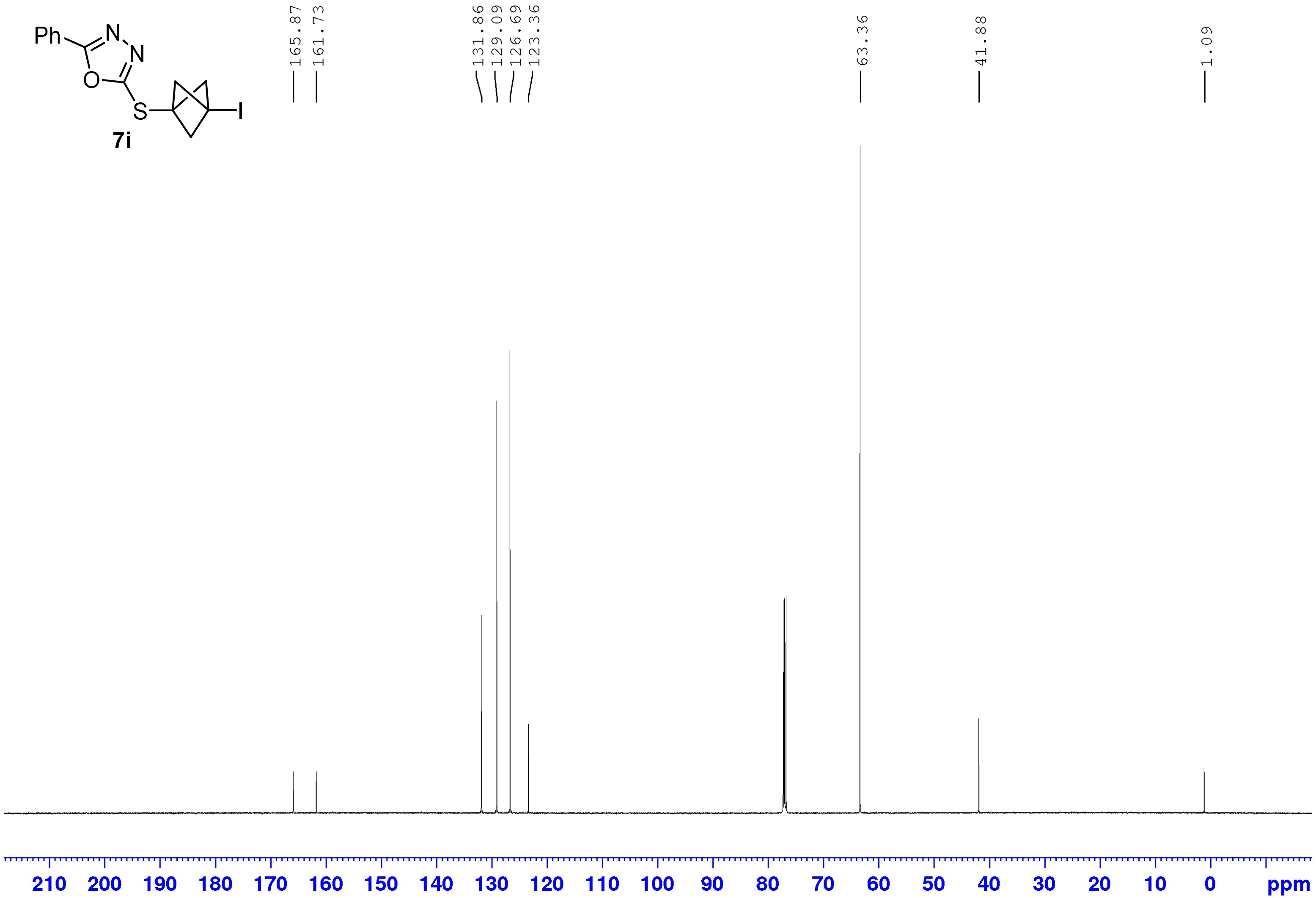

Figure S20. $^{13}C\{^{1}H\}$ NMR (126 MHz, $CDCl_3$) of compound **7i**

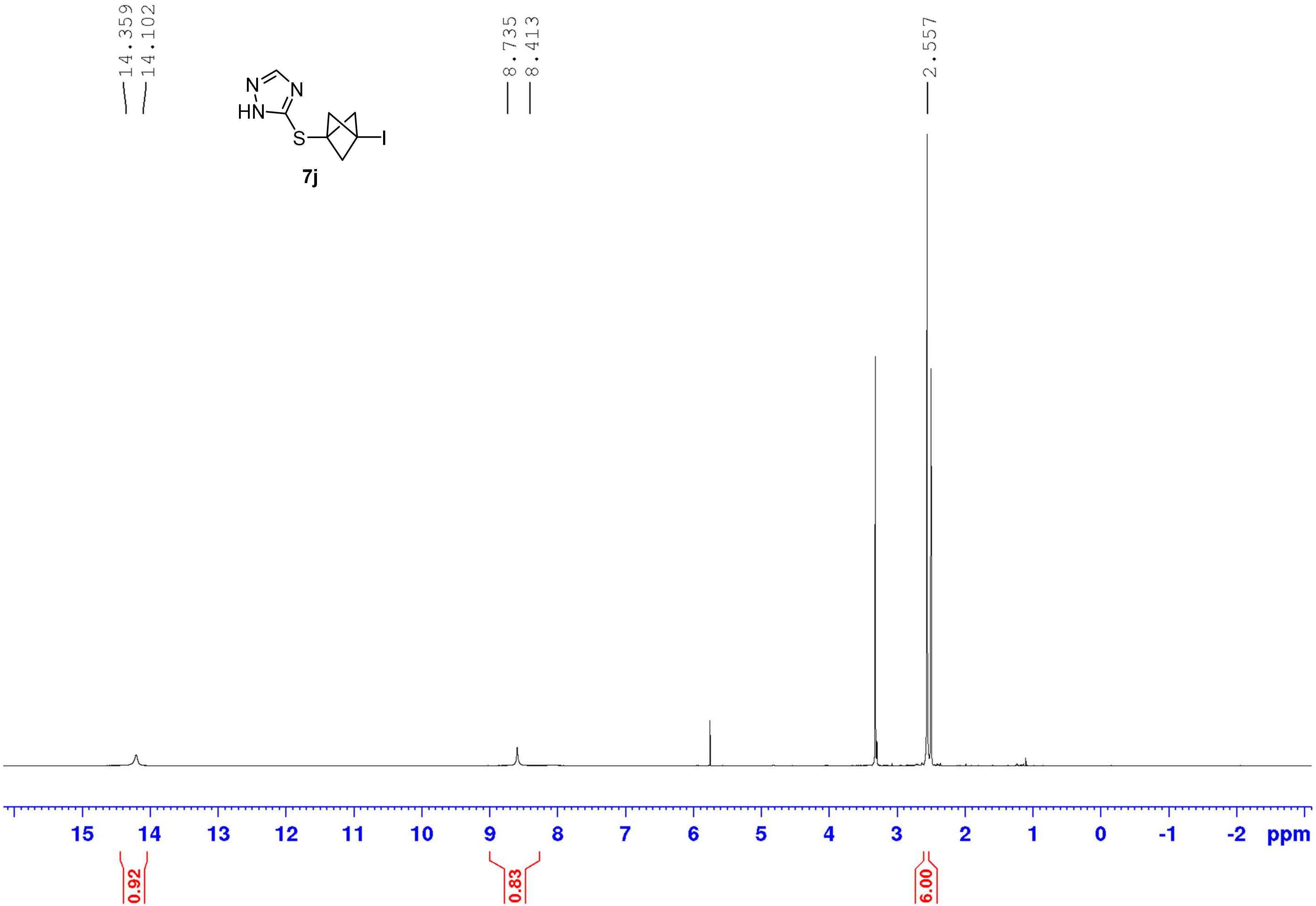

Figure S21. ^{1}H NMR (500 MHz, $CDCl_3$) of compound **7j**

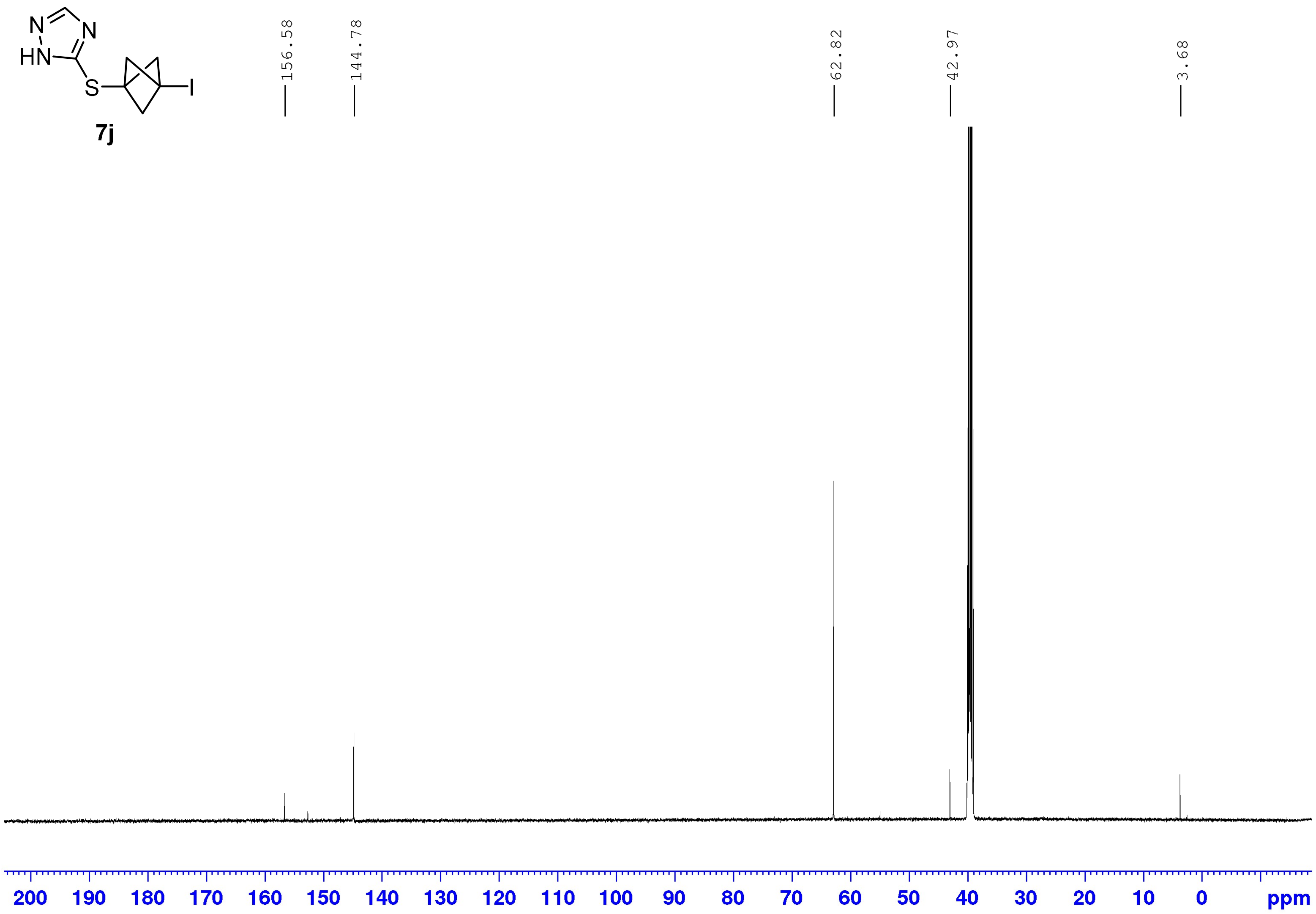

Figure S22. $^{13}C\{^1H\}$ NMR (126 MHz, $CDCl_3$) of compound **7j**

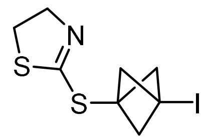

7k

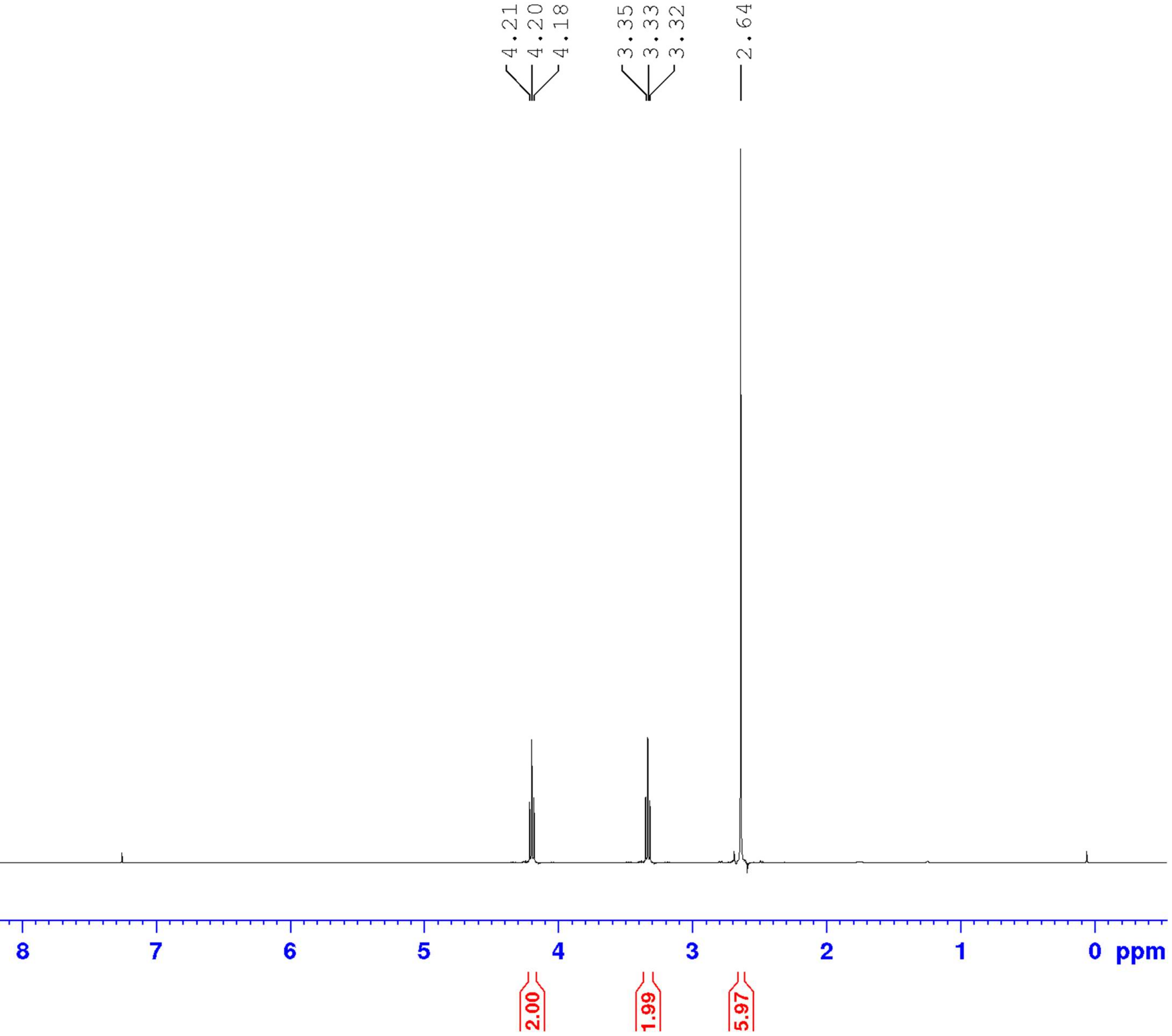

Figure S23. ^{1}H NMR (500 MHz, $CDCl_3$) of compound **7k**

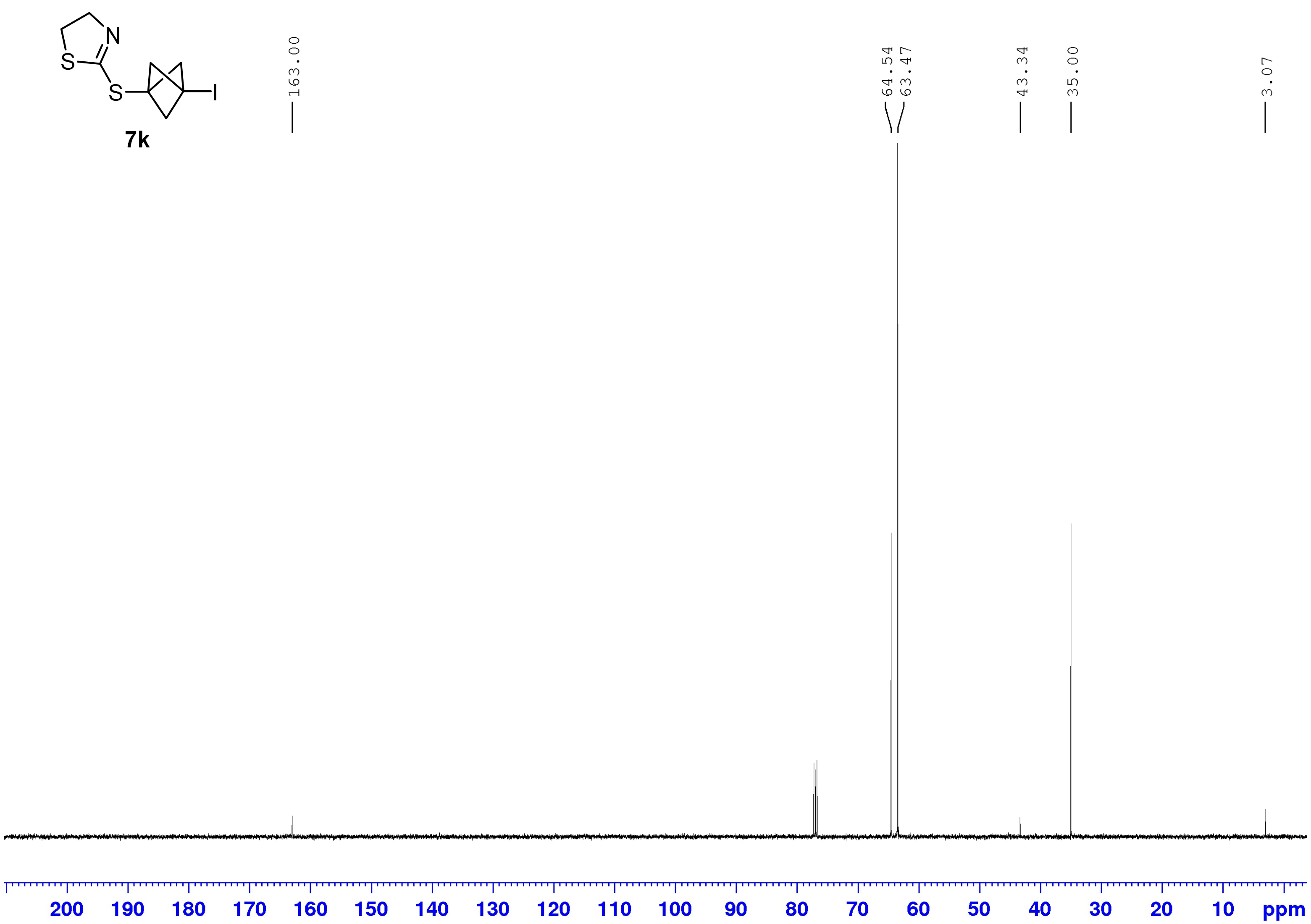

Figure S24. $^{13}C\{^{1}H\}$ NMR (126 MHz, $CDCl_3$) of compound **7k**

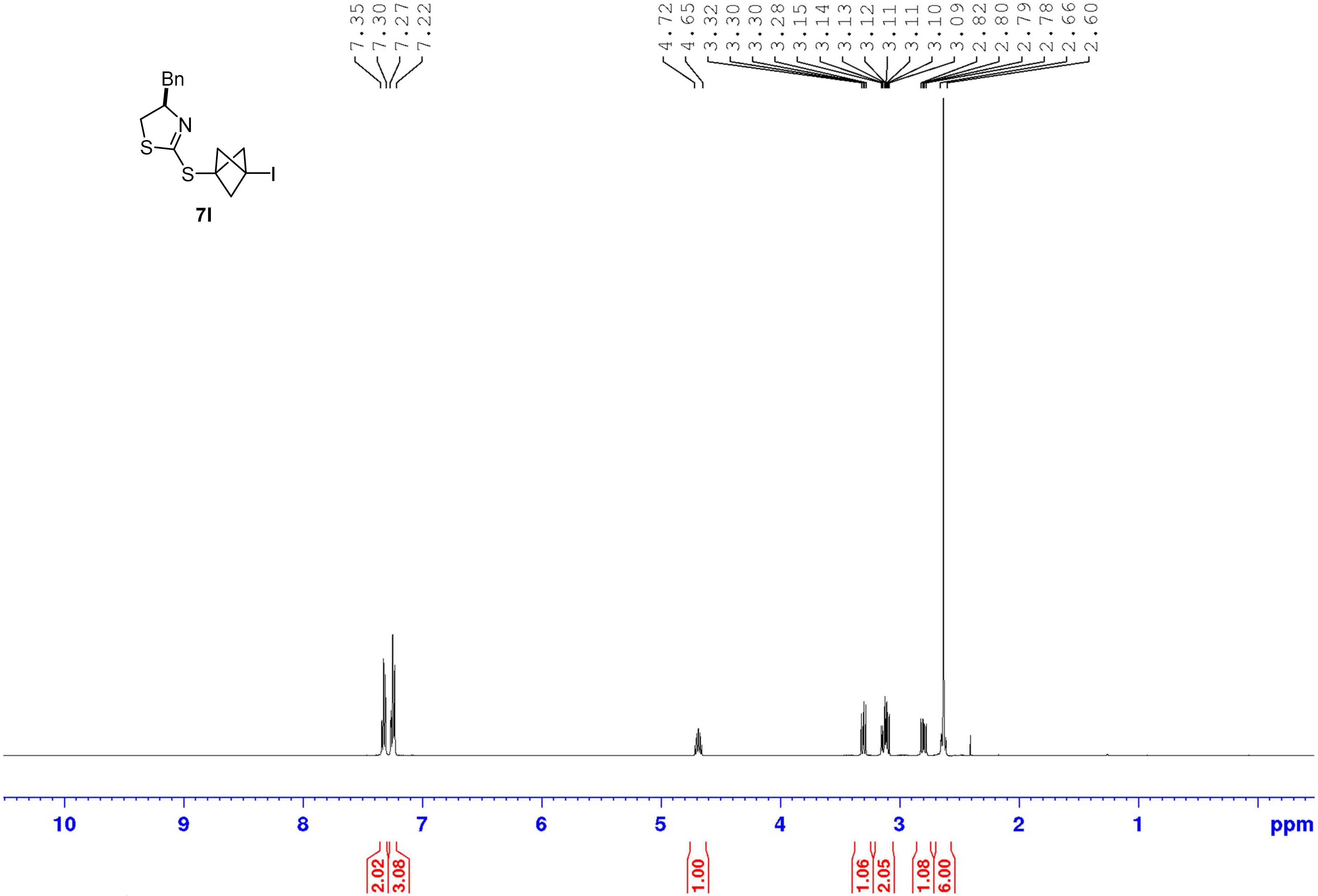

Figure S25. ^{1}H NMR (500 MHz, $CDCl_3$) of compound **7l**

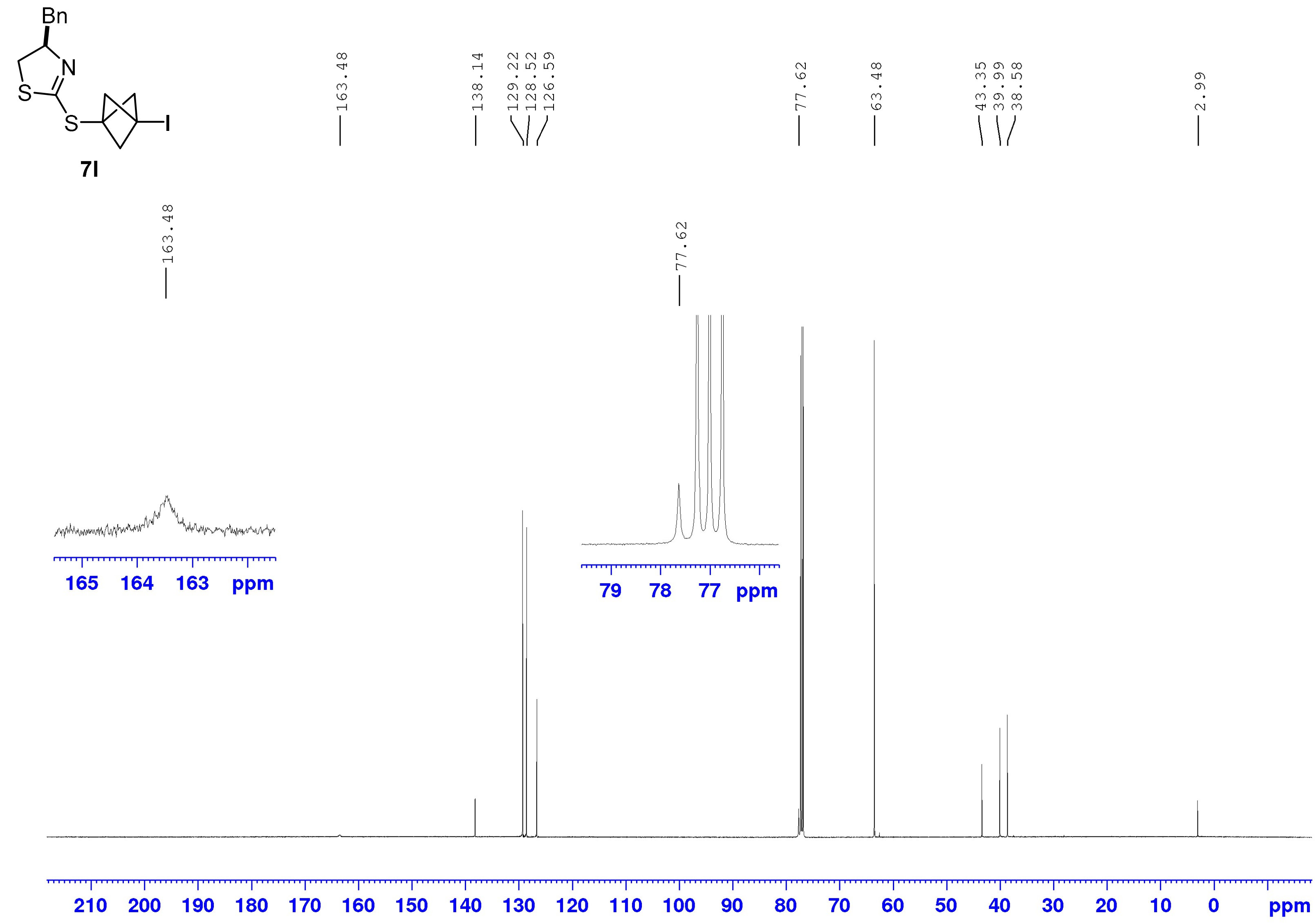

Figure S26. $^{13}C\{^1H\}$ NMR (126 MHz, $CDCl_3$) of compound **7lj**

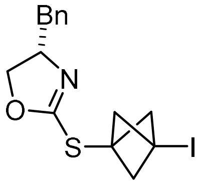

7m

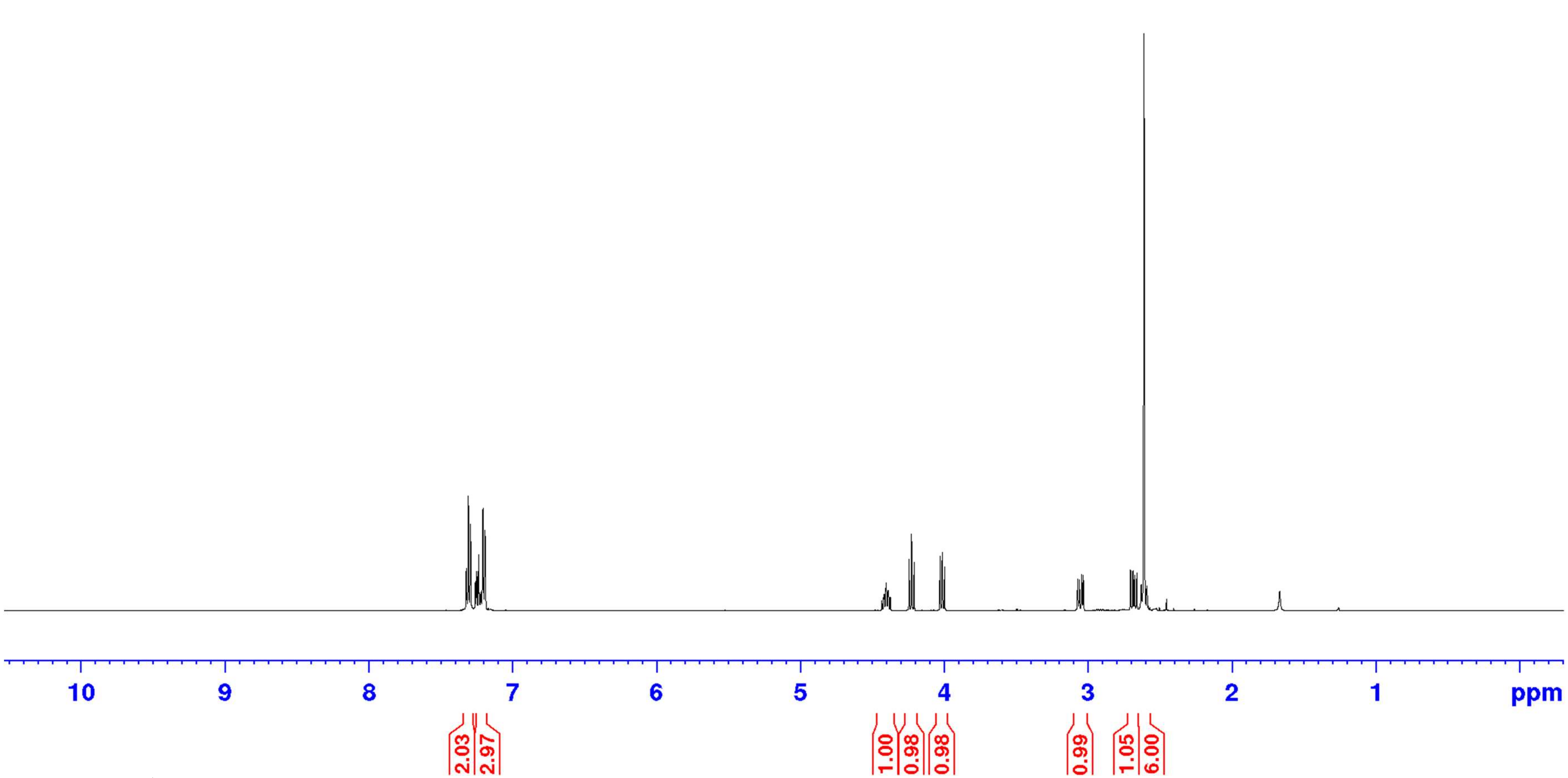

Figure S27. ^{1}H NMR (500 MHz, $CDCl_3$) of compound **7m**

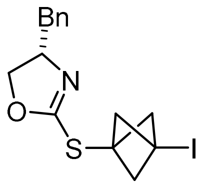

7m

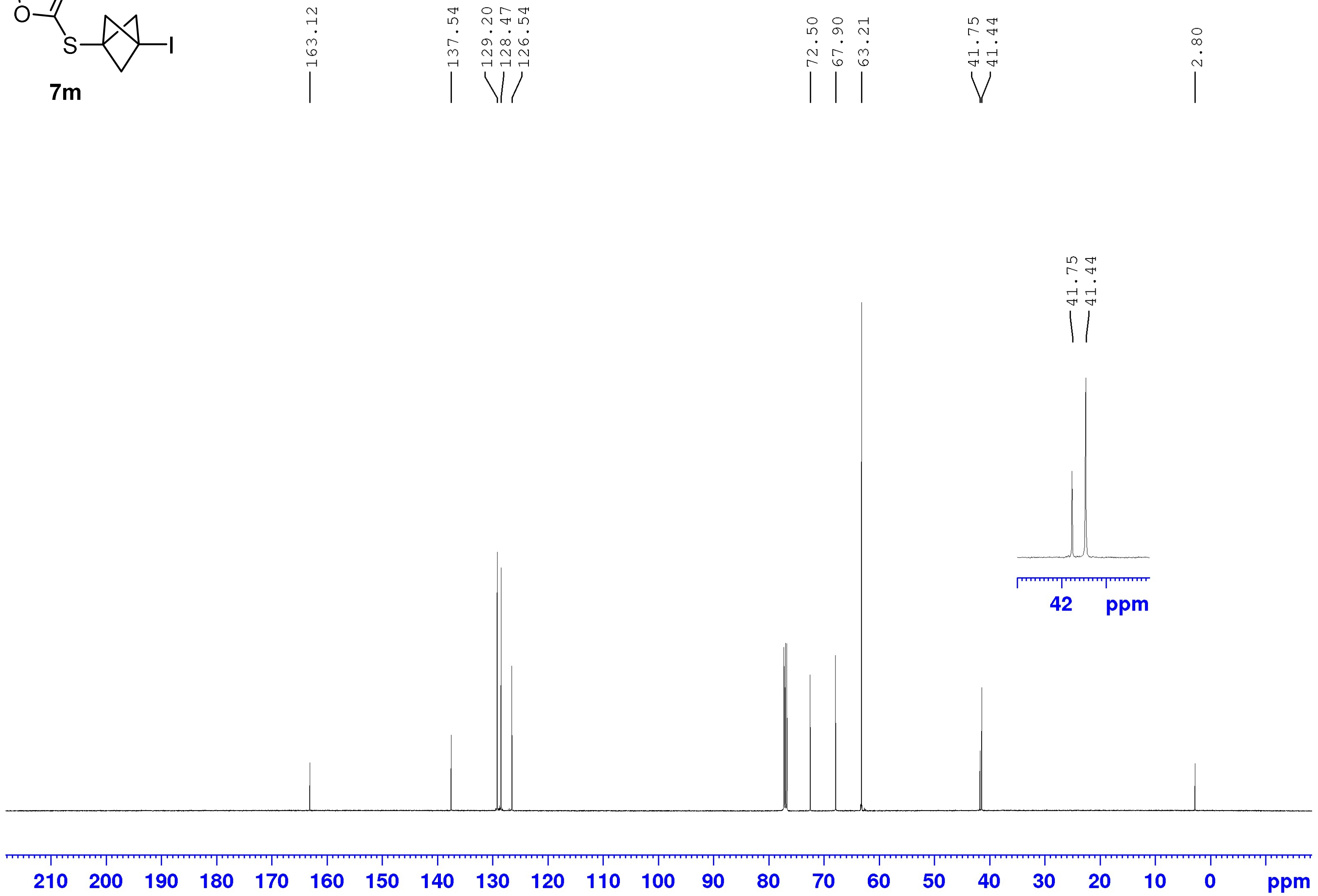

Figure S28. $^{13}C\{^{1}H\}$ NMR (126 MHz, $CDCl_3$) of compound **7m**

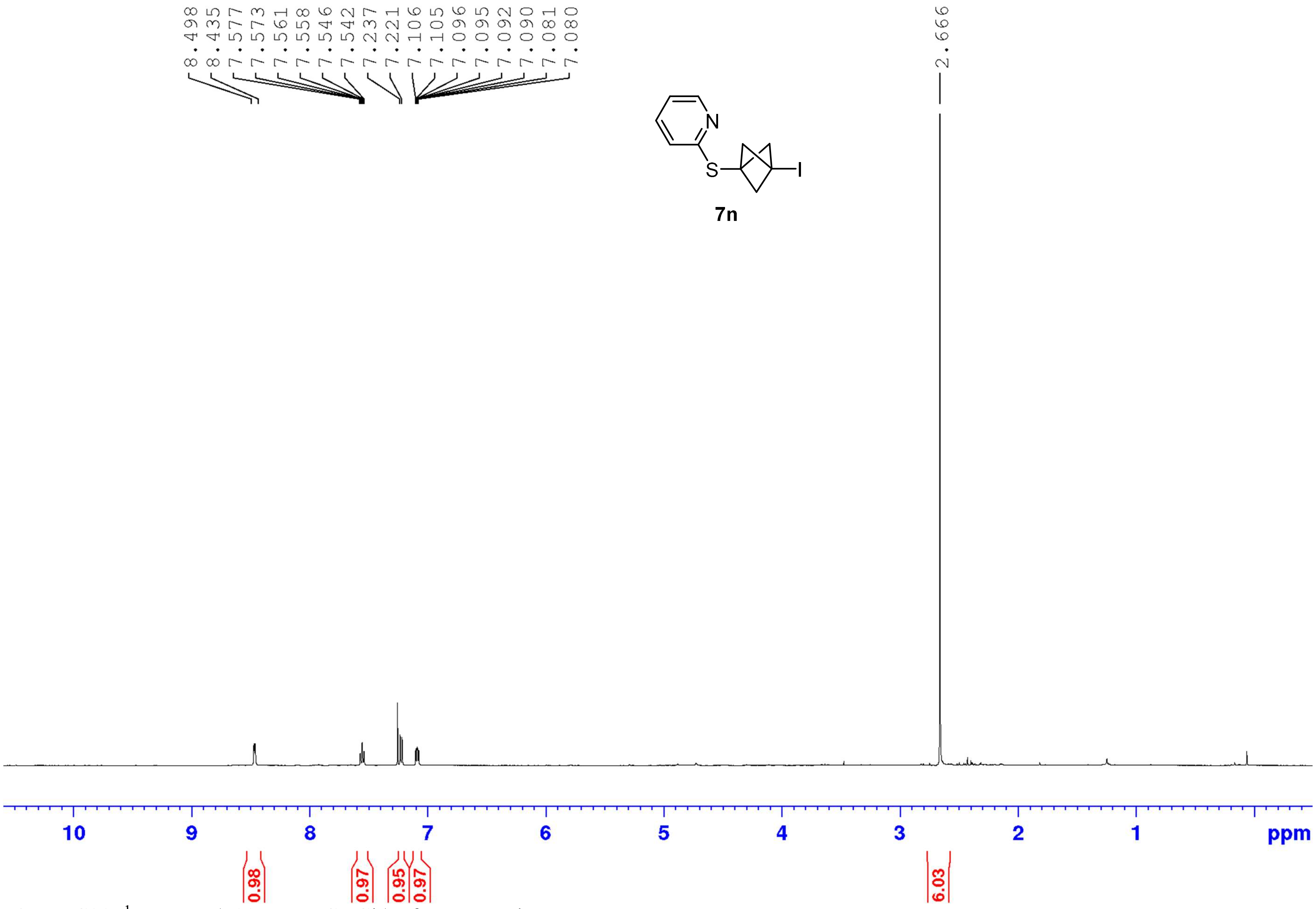

Figure S29. ^{1}H NMR (500 MHz, $CDCl_3$) of compound **7n**

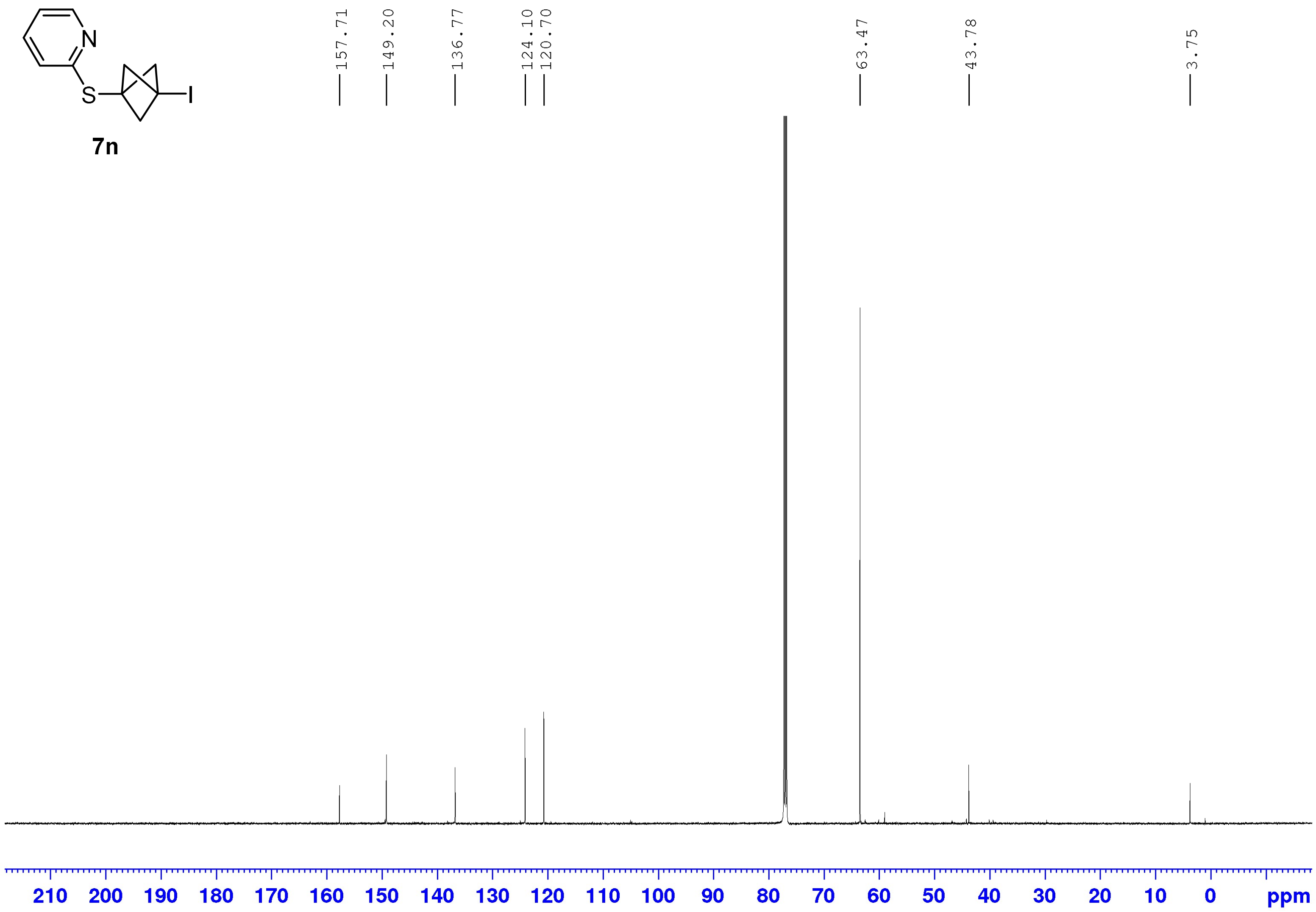

Figure S30. $^{13}C\{^{1}H\}$ NMR (126 MHz, $CDCl_3$) of compound **7n**

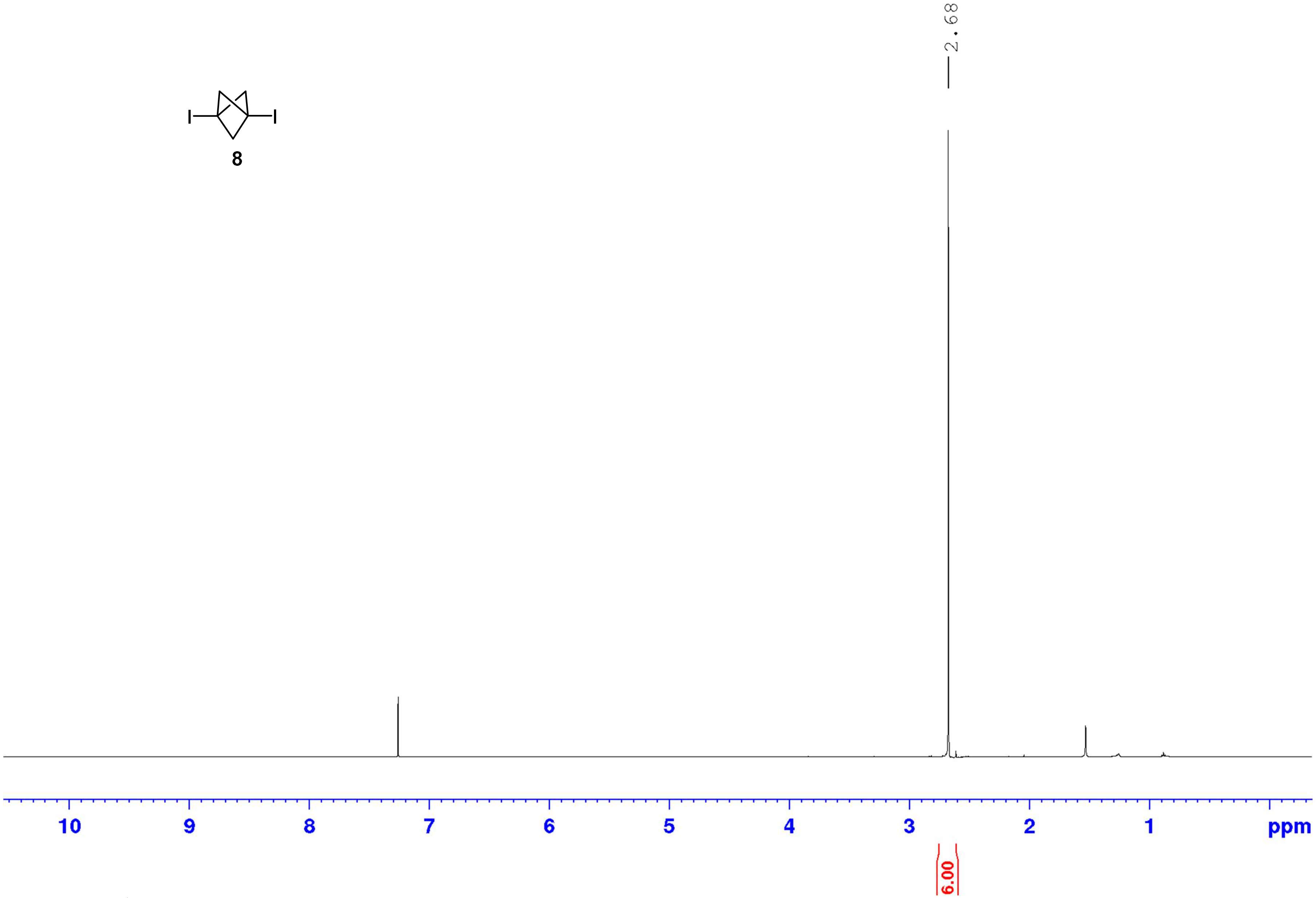

Figure S31. ^{1}H NMR (500 MHz, $CDCl_3$) of compound **8**

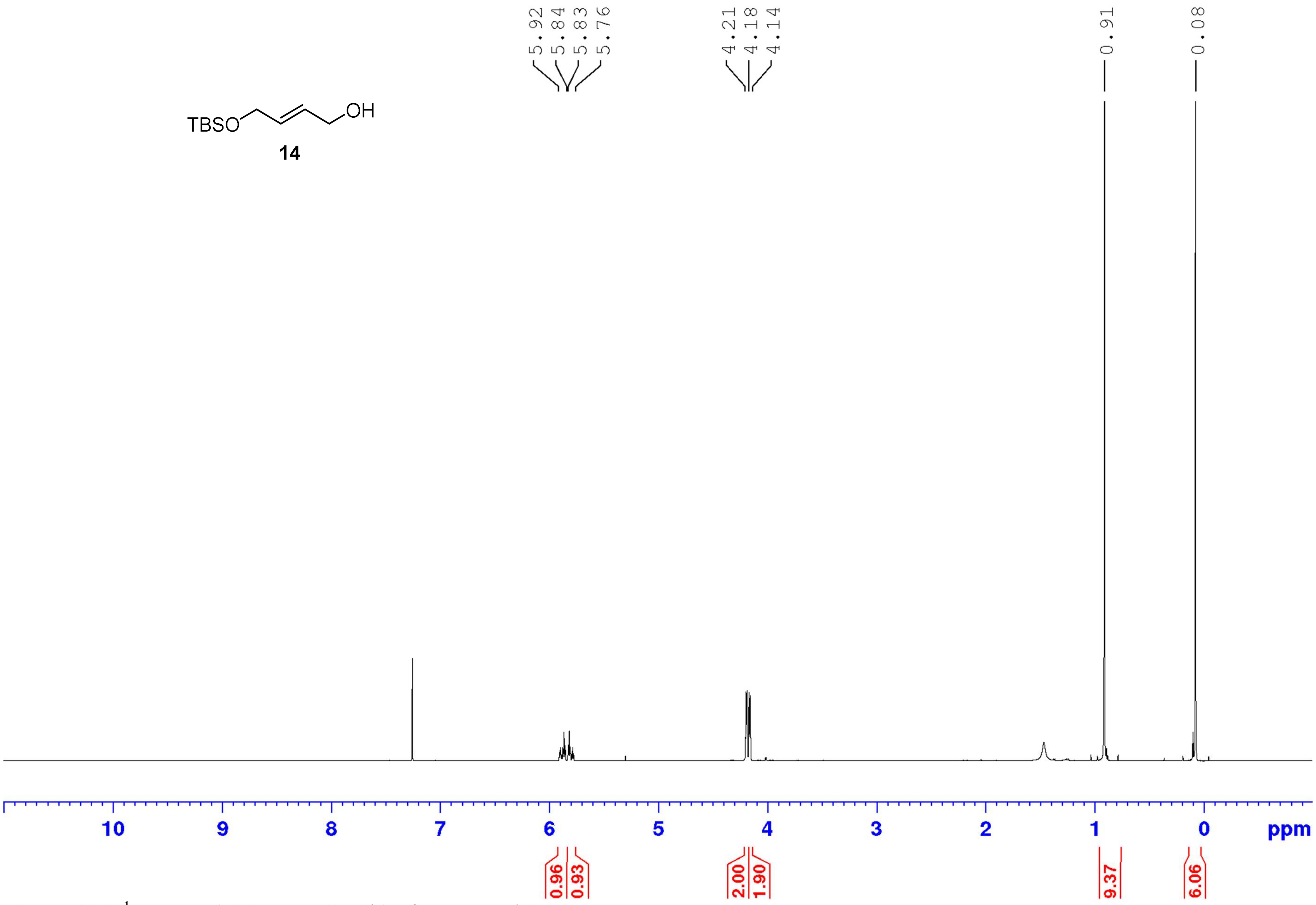

Figure S32. ^{1}H NMR (500 MHz, $CDCl_3$) of compound **14**

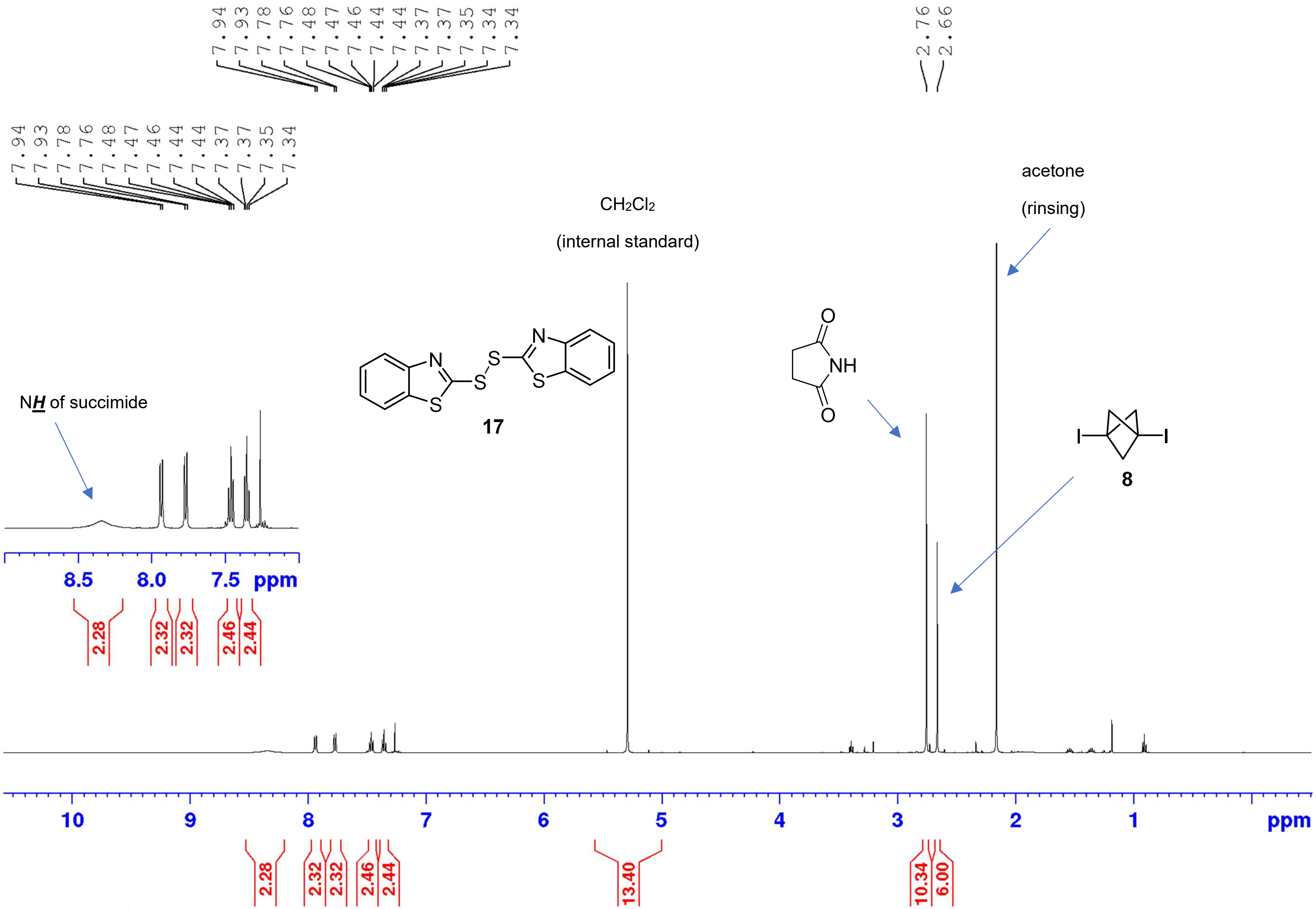

Figure S33. ^{1}H NMR (500 MHz, $CDCl_3$) of compound **17** (crude sample)

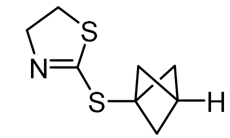

18

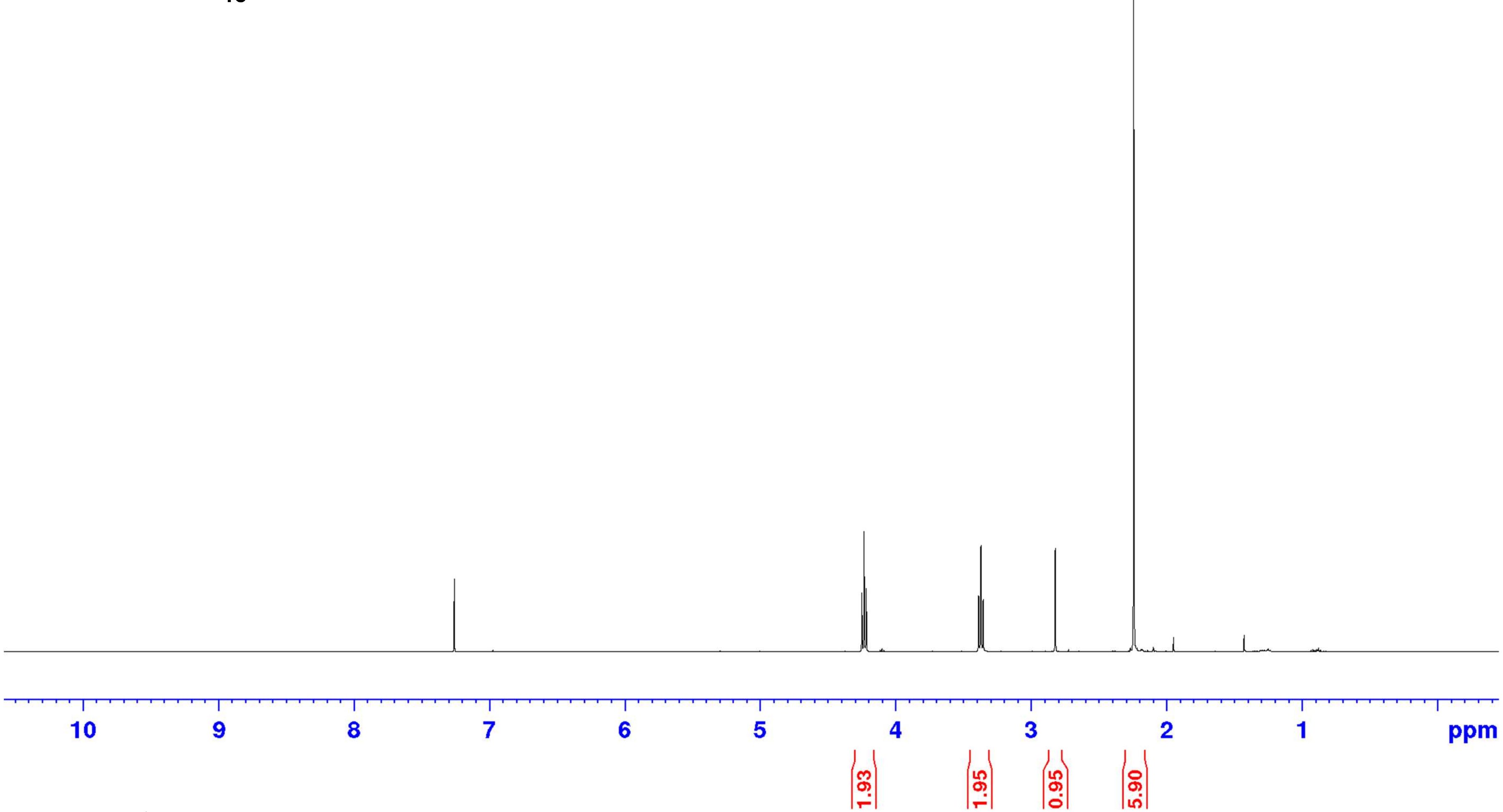

Figure S34. ^{1}H NMR (500 MHz, $CDCl_3$) of compound **18**

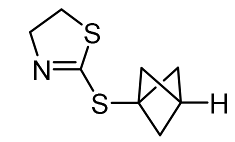

18

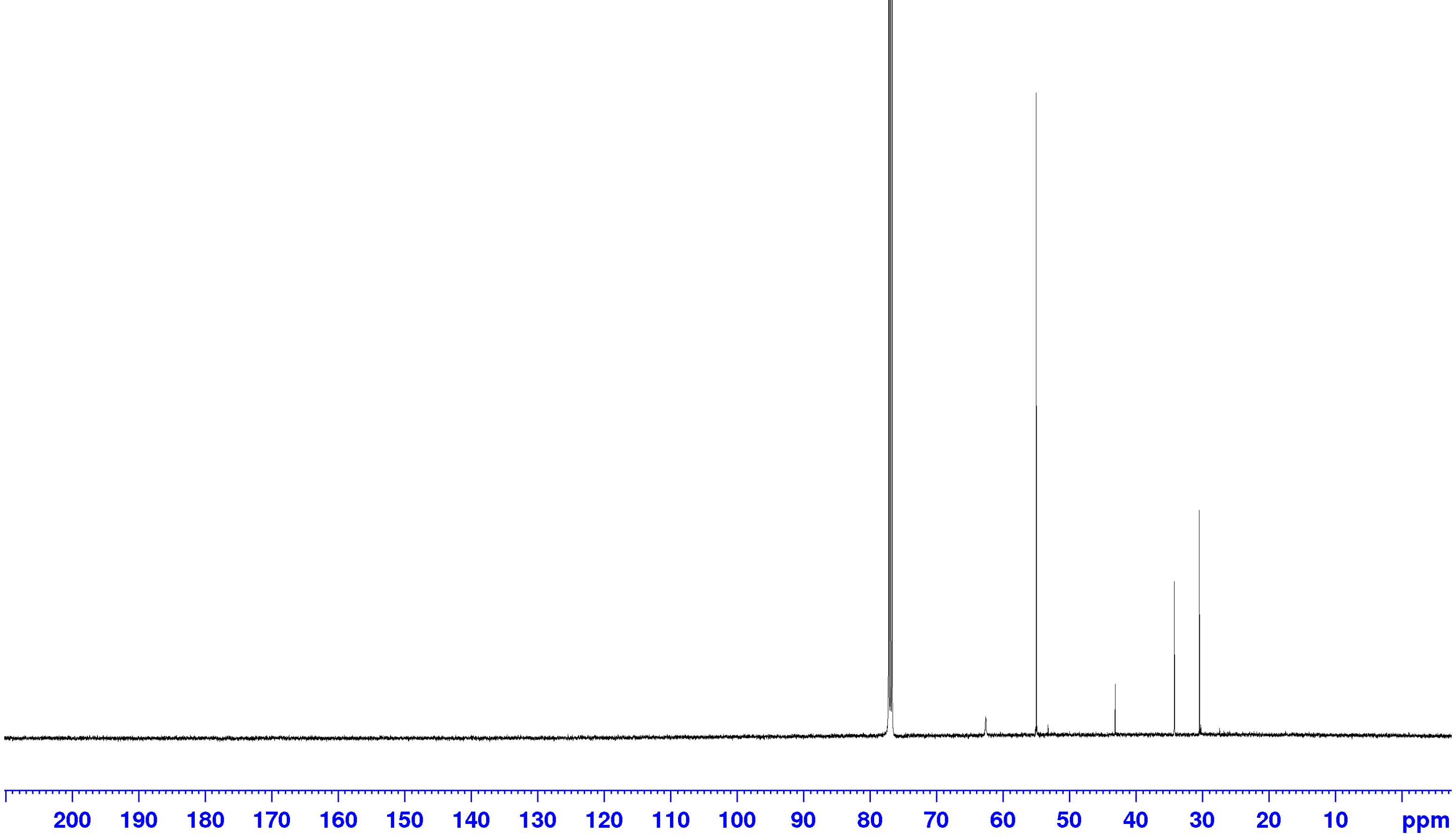

Figure S35. $^{13}C\{^{1}H\}$ NMR (126 MHz, $CDCl_3$) of compound **18**

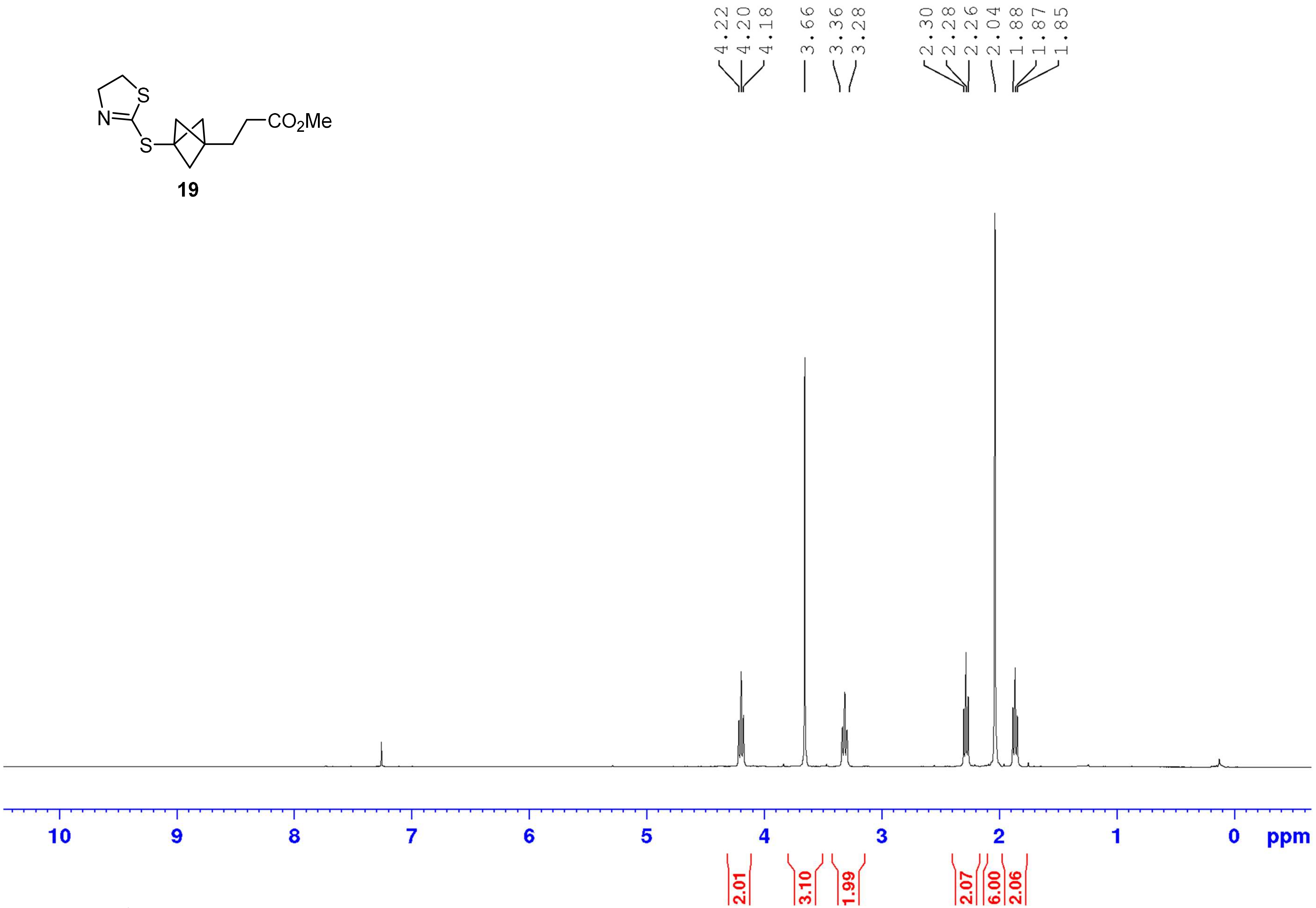

Figure S36. ^{1}H NMR (500 MHz, $CDCl_3$) of compound **19**

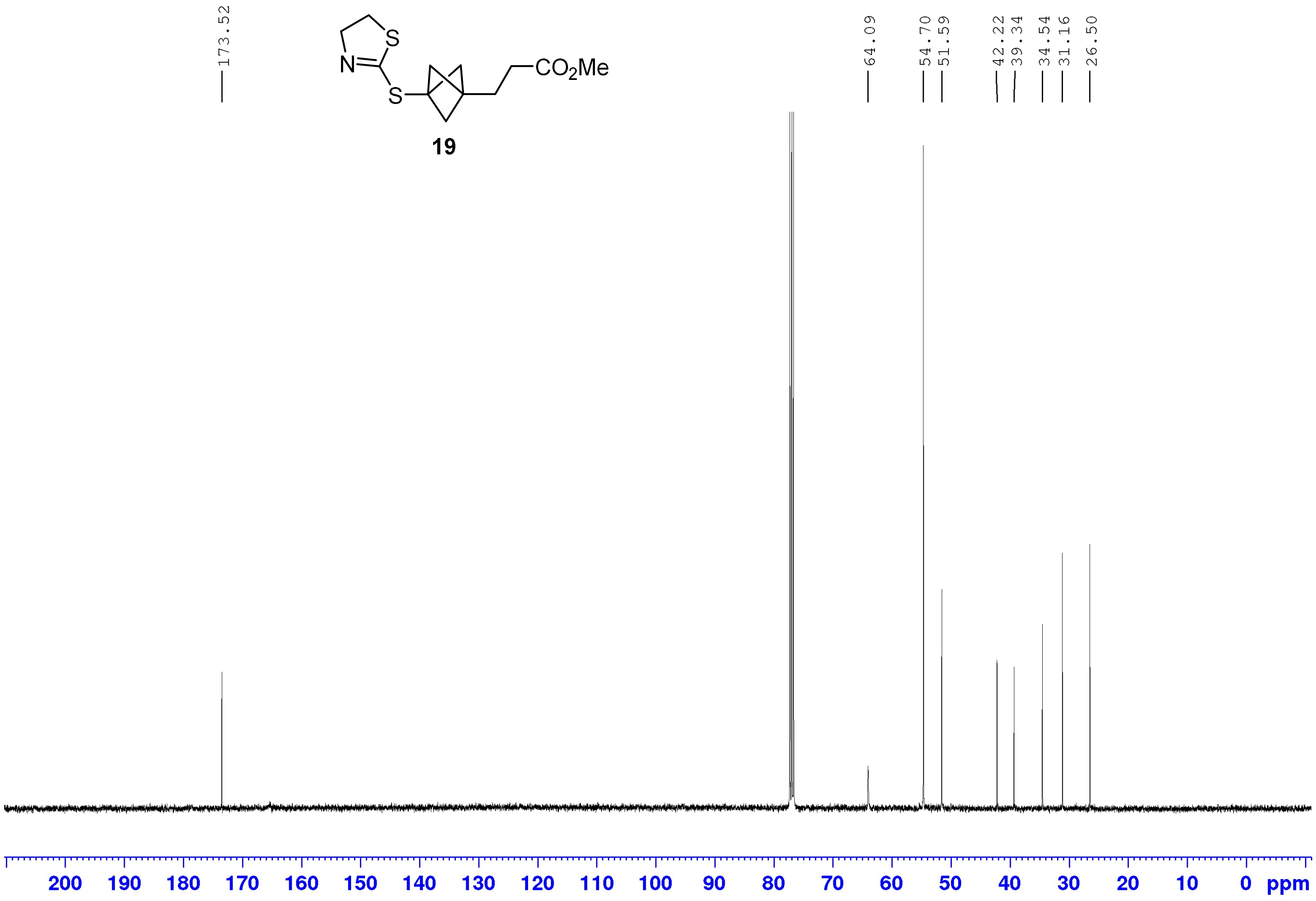

Figure S374. $^{13}C\{^{1}H\}$ NMR (126 MHz, $CDCl_3$) of compound **19**

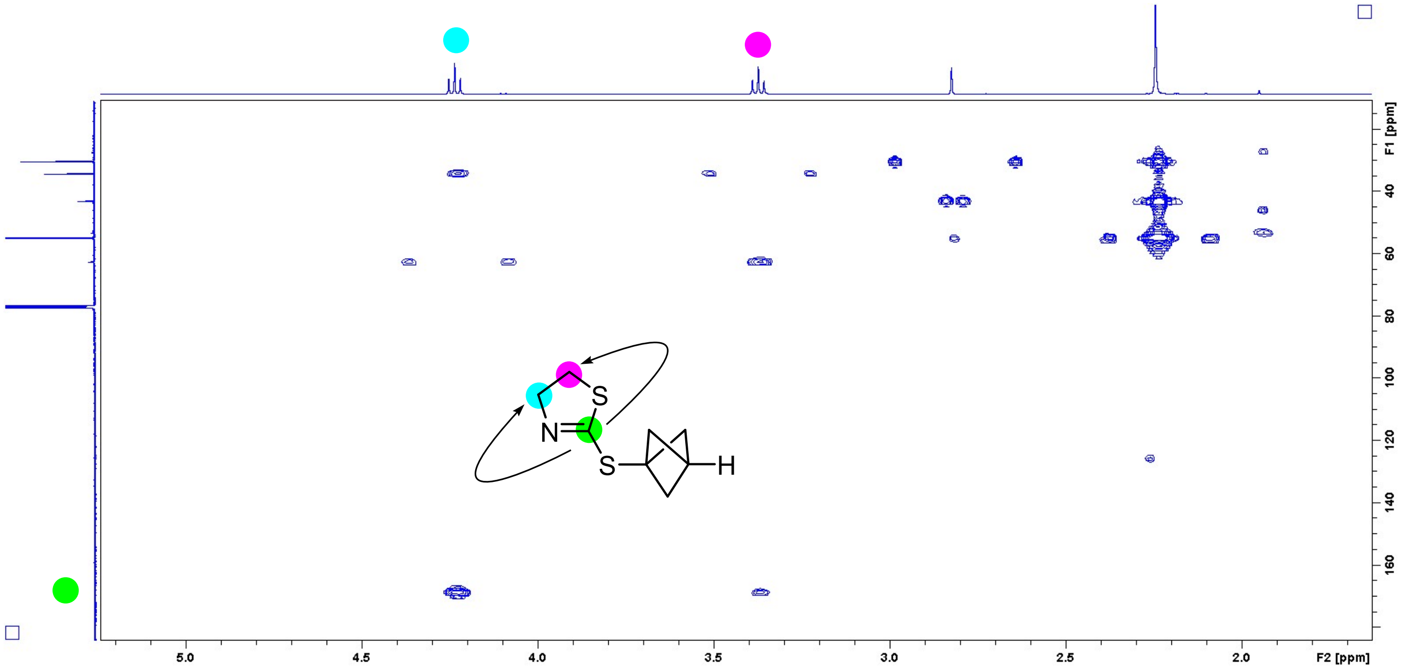

Figure S38. HMBC of compound **18**.

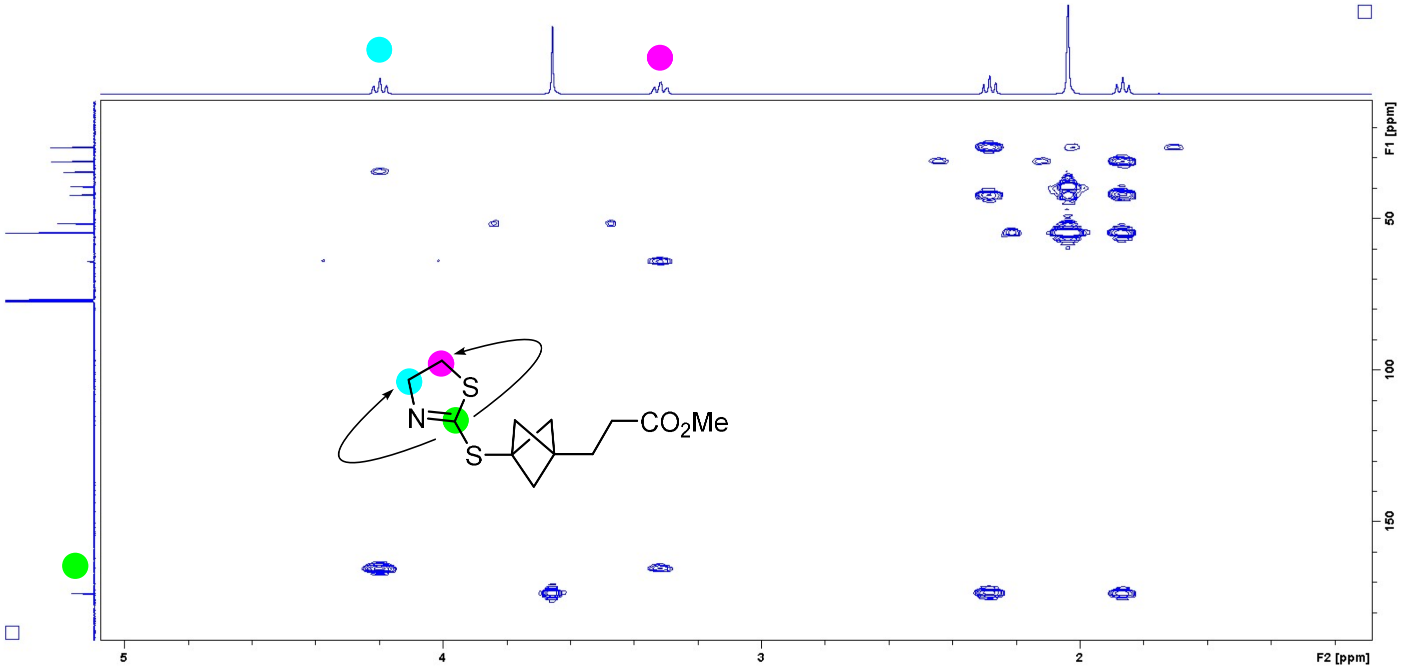

Figure S39. HMBC of compound **19**.

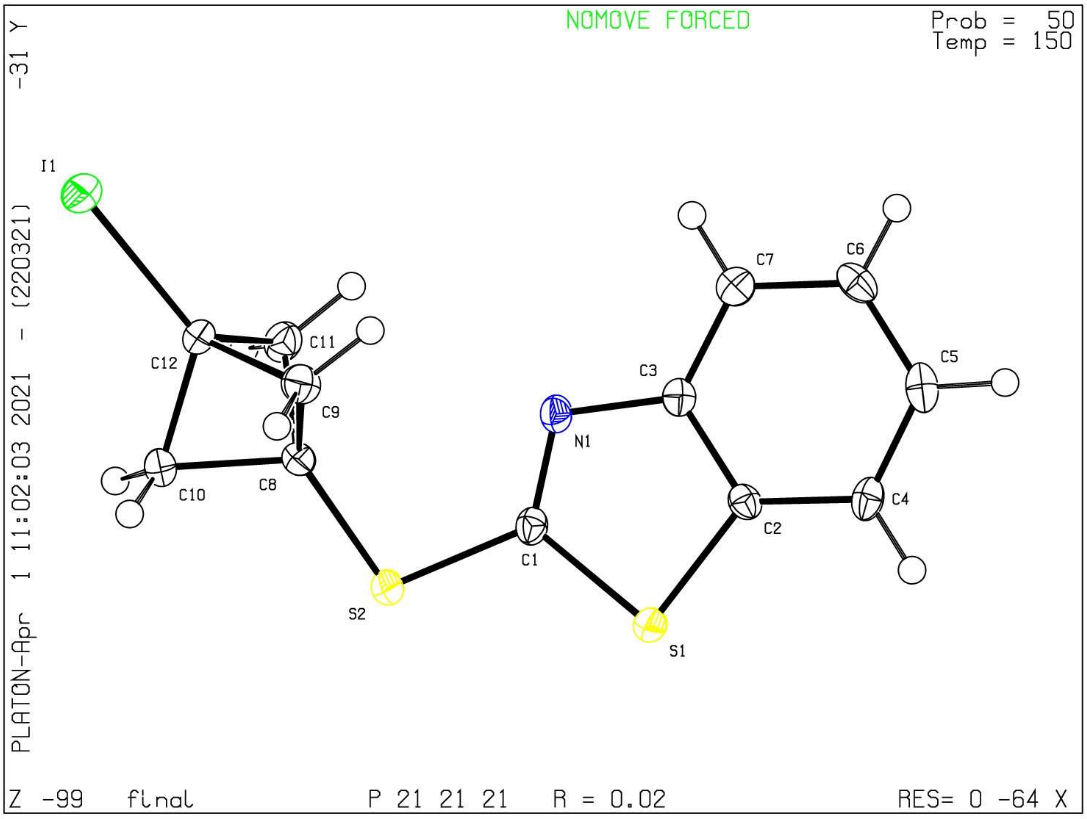

Figure S40. Solid state structure of **7a**. Displacement of ellipsoid plots are drawn at 50% probability. Single crystals were obtained by slow evaporation of a CH_2Cl_2 solution at room temperature.

Table S2 Crystal data and structure refinement for 7a.

Empirical formula	$C_{12}H_{10}INS_2$
Formula weight	359.23
Temperature/K	150
Space group	P 21 21 21
a/Å	6.1354 (5)
b/Å	7.0538 (6)
c/Å	29.270 (2)
α/°	90
β/°	90
γ/°	90
Volume/Å^3	1266.74 (17)
Z	4
ρ_{calc} g/cm^3	1.884
μ/mm^{-1}	2.829
F(000)	696.0
Radiation	MoKα (λ = 0.71073)
Index max (h, k, l)	7, 8, 36
Data/restraints/parameters	2609/0/145
Goodness-of-fit on F^2	1.201
Final R indexes [all data]	R_1 = 0.0161, wR_2 = 0.0389

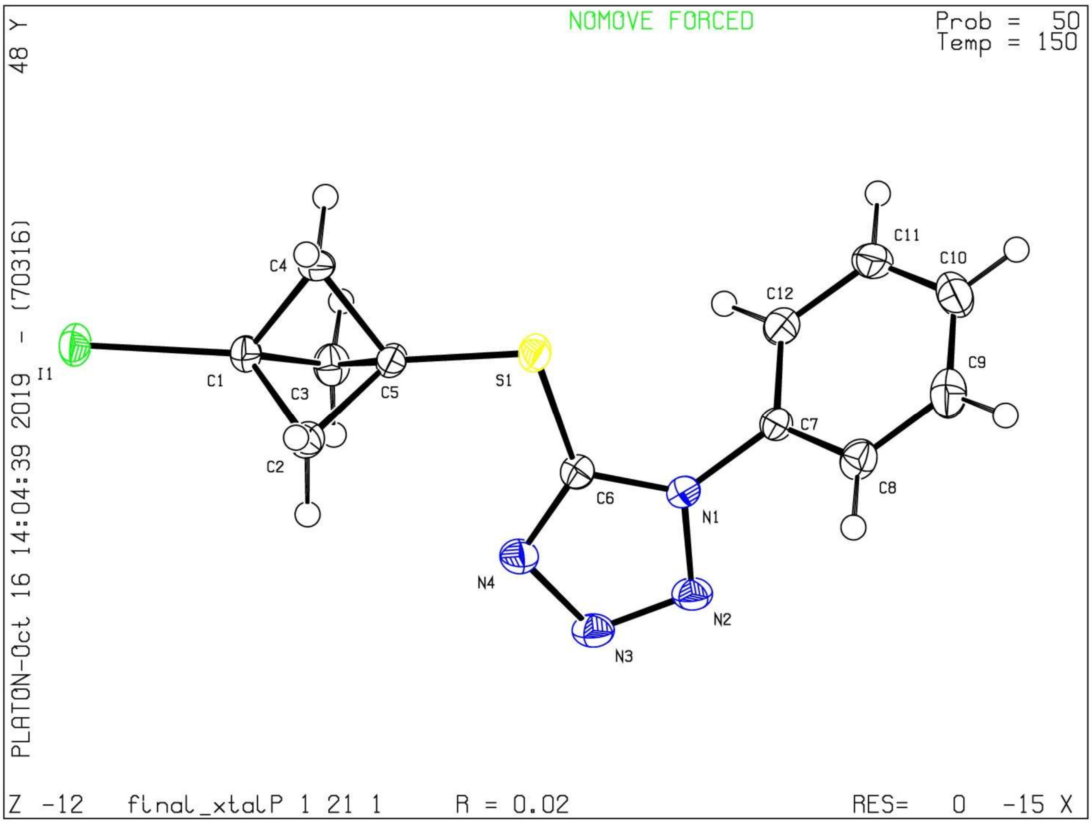

Figure S41. Solid state structure of **7e**. Displacement of ellipsoid plots are drawn at 50% probability. Single crystals were obtained by slow evaporation of a CH_2Cl_2 solution at room temperature.

Table S3 Crystal data and structure refinement for 7e.

Empirical formula	$C_{12}H_{11}IN_4S$
Formula weight	370.21
Temperature/K	150
Space group	P 21
a/Å	6.4571 (8)
b/Å	10.8018 (12)
c/Å	9.7976 (11)
α/°	90
β/°	92.727
γ/°	90
Volume/Å^3	682.59 (14)
Z	2
ρ_{calc} g/cm^3	1.801
μ/mm^{-1}	2.486
F(000)	360.0
Radiation	MoKα (λ = 0.71073)
Index max (h, k, l)	8, 13, 12
Data/restraints/parameters	2772/0/163
Goodness-of-fit on F^2	1.045
Final R indexes [all data]	R_1 = 0.0153, wR_2 = 0.0331

DSC for compound 7e

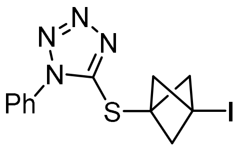

7e

DSC test (40µl HP Gold; 5 °C /min) on 2.4 mg of **7e** showed a sharp endotherm (65 J/g) from 128 °C to ~157°C directly followed by a large incomplete exotherm (>2100 J/g) from ~157 °C to the test end at ~500°C. A Yoshida plot of the DSC test data indicates that the material is a potential explosive and has a high risk of being shock sensitive.

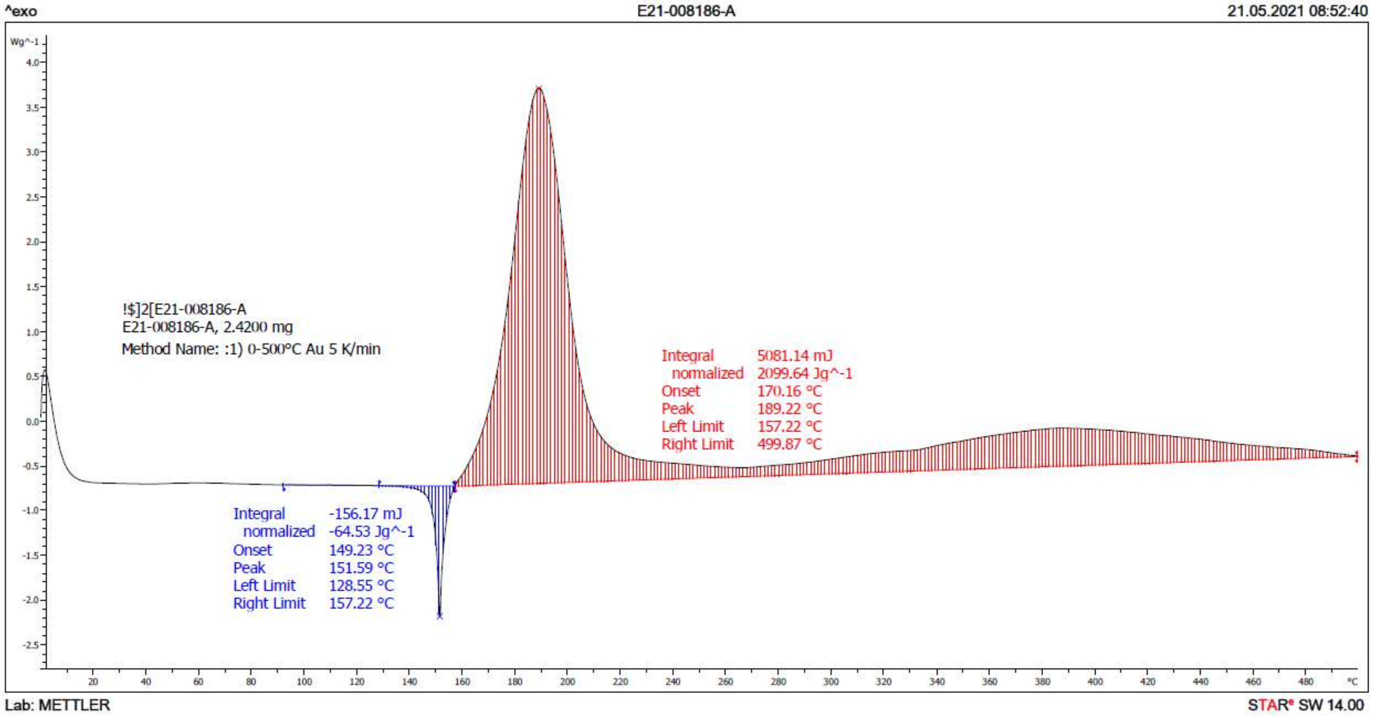

Figure S42. DSC plot of compound **7e**

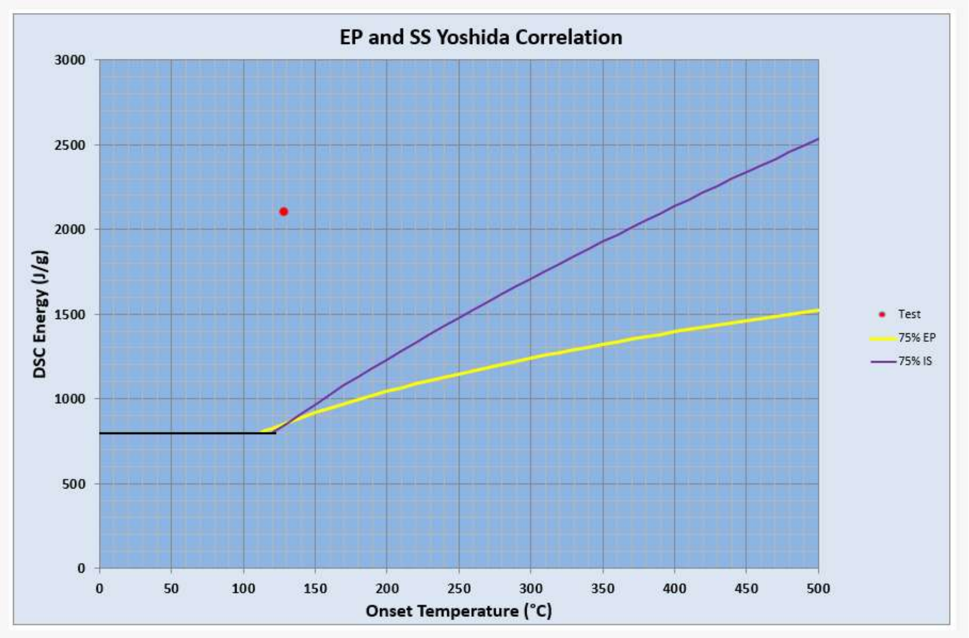

Figure S43. Yoshida plot of compound **7e**
